# Supplementary material for: The transcription factor HIF2α partakes in the differentiation block of acute myeloid leukemia
Source: EMBO Mol Med. 2023 Oct 9;15(11):e17810. doi: 10.15252/emmm.202317810 (PMC10630882; doi:10.15252/emmm.202317810)
Supplement: Supplementary file 1 — Appendix [file EMMM-15-e17810-s006.pdf]

Table of contents to the appendix file

Contents	Page
Appendix Figure S1	2
Appendix Figure S2	3
Appendix Figure S3	4
Appendix Figure S4	5
Appendix Figure Legends	6-8
Appendix Table S1	9-12
Appendix Table S2	13-15
Appendix Table S3	16
Appendix Table S4	17

Appendix Figure S1

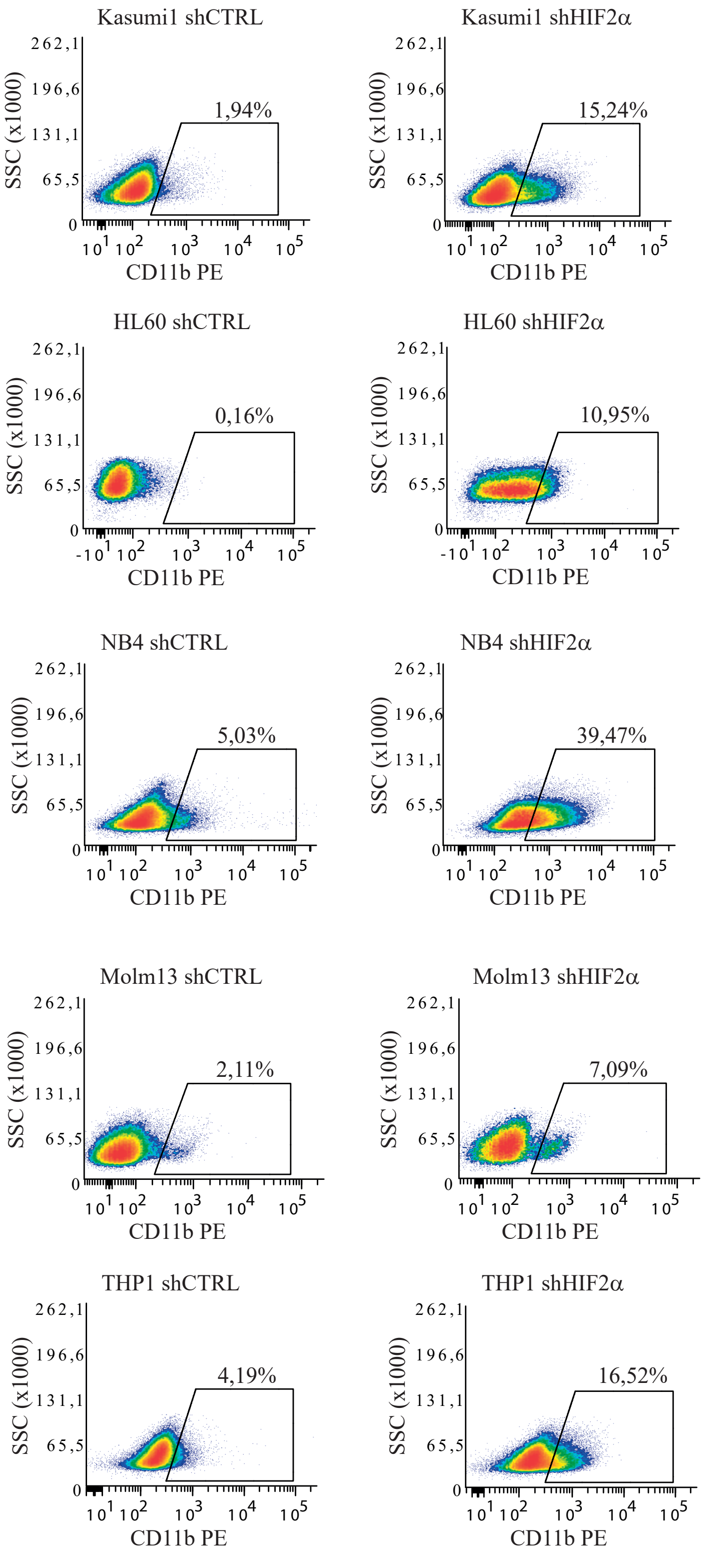

Appendix Figure S2

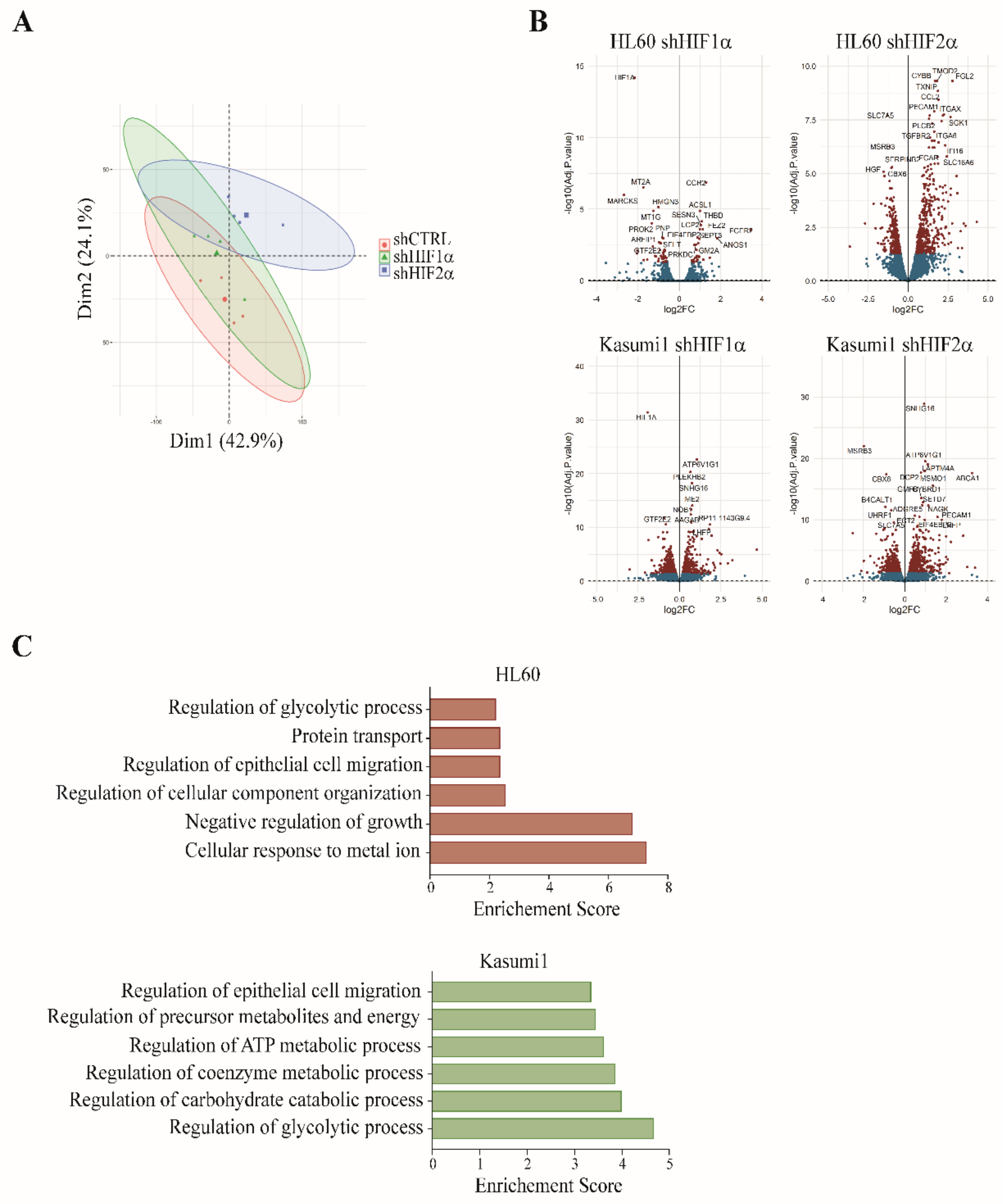

Appendix Figure S3

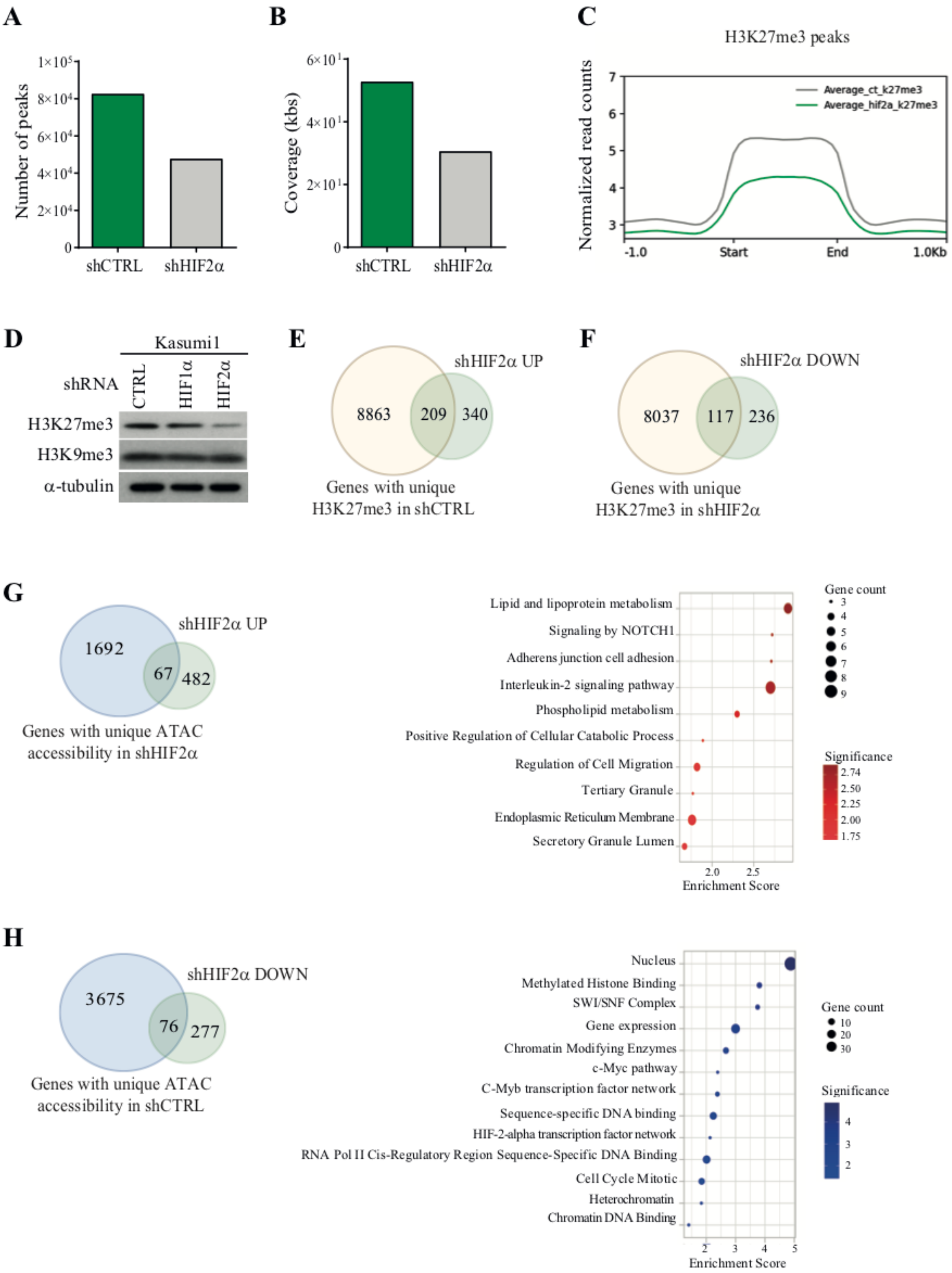

Appendix Figure S4

A

PDX	Chromosomal abnormality	Main genetic lesions	Immunophenotype
AML-01	Normal cytogenetic	NPM1, DNMT3A, FLT3-ITD, IDH1	CD4+/CD7+/CD11b+/CD11c+/CD13+/CD33+/CD38+/CD45RA+/CD117+
AML-02	Normal cytogenetic	NPM1, DNMT3A, FLT3-ITD	CD24+/CD7+/CD11b+/CD13+/CD33+/CD34+/CD38+/CD45RA+/CD117+

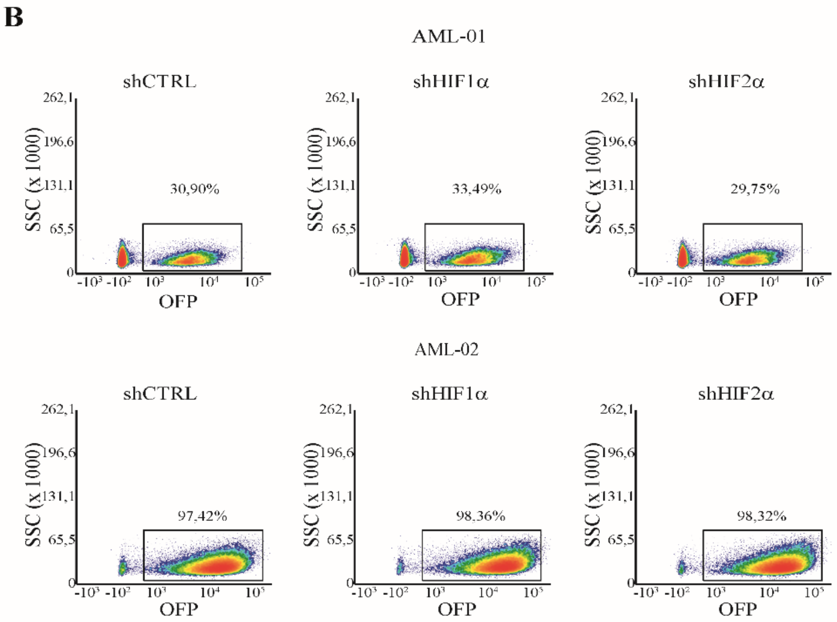

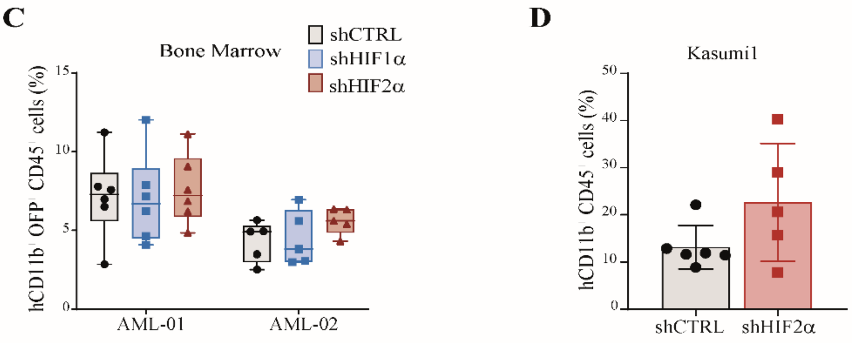

Appendix Figure Legends

Appendix Figure S1. Analysis of CD11b expression upon HIF2α silencing in AML cell lines. Representative flow cytometry profiles of CD11b and side scatter (SSC) in Kasumi1 and HL60 cell lines with control shRNA (shCTRL) or shRNA against HIF2α (shHIF2α). Gates were set over PE isotype control antibody.

Appendix Figure S2. Global changes in gene expression upon HIF1α or HIF2α silencing in AML cells. A. Principal component analysis (PCA) on differentially expressed genes (DEGs) in shHIF1α, shHIF2α, and shCTRL HL60 and Kasumi1 cells. Ellipses identify clusters, the centroid represents the barycenter of the points belonging to the same cluster. **B.** Volcano plots depicting DEGs in shHIF1α and shHIF2α HL60 and Kasumi1 cells with respect to shCTRL cells. Red and blue dots correspond respectively to significantly and non-significantly deregulated genes. A significant threshold of 0.05, adjusting the p-value by FDR (False Discovery Rates), was established to identify DEGs. **C** Representative Gene Ontology (GO) terms within the most significant families of downregulated genes in shHIF1α HL60 and Kasumi1 cells. Data are presented as Enrichment Score ($-\log_{10}$(p-value)) over shCTRL cells.

Appendix Figure S3. Global changes in H3K27me3 and chromatin accessibility via ATAC-seq upon HIF2α silencing in Kasumi1 cells. A, B. Number and coverage in kilobases (kbs) of H3K27me3 peaks in shCTRL and shHIF2α cells. **C.** H3K27me3 levels in peaks identified in shCTRL and shHIF2α cells **D.** Immunoblot analysis of H3K27me3 and H3K9me3 in Kasumi1 cells carrying a scrambled shRNA as control (CTRL) or shRNAs against HIF1α and HIF2α. α-tubulin was used as loading control. **E.** Overlap of genes linked to unique H3K27me3 peaks in shCTRL cells and genes upregulated (UP) upon HIF2α silencing

(shHIF2α). **F.** Overlap of genes mapping to unique H3K27me3 peaks in shHIF2α cells and genes downregulated (DOWN) upon HIF2α silencing (shHIF2α). **G.** Overlap of genes mapping to unique ATAC peaks in HIF2α silenced cells and genes upregulated (UP) upon HIF2α silencing (shHIF2α, left Venn diagram). Gene set enrichment analysis of genes regulated by HIF2α and showing changes in chromatin accessibility upon HIF2α silencing (right plot). **H.** Overlap of genes mapping to unique ATAC peaks in control cells and genes downregulated (DOWN) upon HIF2α silencing (shHIF2α, left Venn diagram). Gene set enrichment analysis of genes regulated by HIF2α and showing changes in chromatin accessibility upon HIF2α silencing (right plot). In **G** and **H**, the following libraries were used: gene ontology (GO) biological process, GO molecular function, GO cellular component, Bioplanet, Reactome, and Hallmarks of cancer. Dot sizes represent the number of genes in each term, and colors indicate Enrichment Scores expressed as $-\log_{10}$(p-value).

Appendix Figure S4. Silencing of HIFα factors in PDX-derived AML cells. A. Genetic and immunophenotypic features of the two AML PDX models utilized in this work. **B.** FACS analysis performed at 96 hours after infection of AML-01 and AML-02 cells with lentiviral vectors carrying specific shRNAs and orange fluorescent protein (OFP). Shown is the transduction efficiency in AML-01 and AML-02 cells. Data are representative of one out of three experiments with similar results. **C.** Percentage of leukemic cells expressing the myeloid differentiation marker CD11b ($hCD11b^{+}OFP^{+}CD45^{+}$) in the bone marrow of AML-01 (n=6) and AML-02 (n=5) mice carrying shCTRL, shHIF1α, or shHIF2α. Data are shown in box and whisker plots where the central band denotes the median value, box contains interquartile ranges, while whiskers mark minimum and maximum values. All biological replicates are shown (n=5/6). **D.** Percentage of leukemic cells expressing the myeloid differentiation marker

CD11b (hCD11b$^+$CD45$^+$) in Kasumi1 cells carrying shCTRL (n=6) or shHIF2α (n=5) injected subcutaneously.

Appendix Table S1. List of 118 genes commonly upregulated upon HIF2α silencing in HL60 and Kasumi1 cells.

Gene Symbol	HL60 Log_2FC	HL60 padj	Kasumi1 Log_2FC	Kasumi1 padj	Description
ACSL4	0,73	1,29E-02	0,52	1,62E-05	acyl-CoA synthetase long chain family member 4
ADAP2	1,71	8,41E-03	1,4	5,32E-03	ArfGAP with dual PH domains 2
ADGRE2	1,13	1,92E-04	0,57	2,24E-02	adhesion G protein-coupled receptor E2
AIF1	0,56	4,90E-02	0,66	7,32E-04	allograft inflammatory factor 1
ALDH3A2	0,7	1,10E-02	0,73	4,38E-09	aldehyde dehydrogenase 3 family member A2
ANOS1	1,62	1,35E-03	2,24	4,10E-07	anosmin 1
ANXA5	0,65	1,29E-02	0,38	2,77E-02	annexin A5
APLP2	0,59	7,18E-03	0,61	4,17E-03	amyloid beta precursor like protein 2
APOL1	1,32	1,64E-02	1	2,54E-02	apolipoprotein L1
ASPH	1,04	3,25E-05	0,99	7,38E-06	aspartate beta-hydroxylase
ATG9A	0,92	4,19E-02	1,02	3,43E-06	autophagy related 9A
ATHL1	1,25	5,83E-03	0,68	3,51E-02	protein-glucosylgalactosylhydroxylysine glucosidase
ATP6V1G1	0,84	1,42E-03	0,98	3,13E-20	ATPase H+ transporting V1 subunit G1
CCR1	1,42	1,31E-04	0,88	2,28E-04	C-C motif chemokine receptor 1
CCSER2	0,96	2,17E-02	0,52	3,95E-02	coiled-coil serine rich protein 2
CD300C	0,99	2,68E-02	0,61	1,25E-02	CD300c molecule
CD4	0,65	1,48E-02	0,35	2,25E-02	CD4 molecule
CD53	1,04	4,76E-05	0,78	4,17E-03	CD53 molecule
CREB3L2	0,73	3,73E-03	0,69	8,09E-07	cAMP responsive element binding protein 3 like 2
CTSB	0,6	2,89E-02	0,59	4,32E-05	cathepsin B
CTSZ	1,02	6,14E-04	0,83	1,21E-04	cathepsin Z
CYB5R1	0,61	4,92E-02	0,48	1,50E-02	cytochrome b5 reductase 1
CYBB	1,67	4,90E-10	0,93	4,57E-04	cytochrome b-245, beta polypeptide
CYBRD1	0,79	5,58E-03	1,37	2,63E-16	cytochrome b reductase 1
CYTH4	0,8	3,74E-02	0,76	2,17E-03	cytohesin 4
DCP2	0,75	3,47E-03	0,79	1,94E-18	decapping mRNA 2
DEF8	0,73	2,80E-02	0,58	2,25E-05	differentially expressed in FDCP 8 homolog
EID1	0,57	1,59E-02	0,32	1,63E-02	EP300 interacting inhibitor of differentiation 1

EIF4EBP2	1,02	5,20E-06	0,98	9,74E-11	eukaryotic translation initiation factor 4E binding protein 2
ENTPD5	0,83	3,84E-02	0,56	4,64E-02	ectonucleoside triphosphate diphosphohydrolase 5
EVI2A	1,51	1,00E-05	0,62	4,85E-02	ecotropic viral integration site 2A
FADS1	0,63	8,16E-03	0,85	3,48E-04	fatty acid desaturase 1
FADS3	1,15	4,84E-02	1,11	3,77E-03	fatty acid desaturase 3
FAM135A	0,71	2,93E-02	0,38	3,87E-03	family with sequence similarity 135 member A
FCAR	1,84	1,67E-06	1,25	4,94E-02	Fc fragment of IgA receptor
FEZ2	0,82	7,71E-03	1,03	4,50E-09	fasciculation and elongation protein zeta 2
FGL2	2,78	4,90E-10	0,97	4,72E-02	fibrinogen-like protein 2
FNDC3A	0,81	4,08E-03	0,5	4,28E-03	fibronectin type III domain containing 3A
FNDC3B	0,73	5,71E-03	0,5	1,82E-03	fibronectin type III domain containing 3B
GAA	1,25	2,74E-03	0,78	7,33E-05	alpha glucosidase
GMFB	0,63	1,42E-02	0,81	3,16E-14	glia maturation factor beta
GNS	0,74	2,26E-03	0,54	1,24E-02	glucosamine (N-acetyl)-6-sulfatase
GOLIM4	0,7	1,49E-02	0,44	9,84E-06	golgi integral membrane protein 4
GPCPD1	0,91	4,92E-04	0,48	2,15E-02	glycerophosphocholine phosphodiesterase 1
GPR137B	1,15	9,22E-03	1,09	5,36E-04	G protein-coupled receptor 137B
H6PD	1,88	3,35E-06	1,64	1,97E-05	hexose-6-phosphate dehydrogenase/glucose 1-dehydrogenase
HEXB	0,65	1,85E-02	0,56	1,62E-02	hexosaminidase subunit beta
HLA-E	0,75	1,03E-02	0,29	3,38E-02	major histocompatibility complex, class I, E
HNMT	0,77	1,93E-02	0,48	1,63E-02	histamine N-methyltransferase
ICAM1	3,11	2,90E-02	2,83	4,23E-08	intercellular adhesion molecule 1
IDH1	0,57	9,15E-03	0,41	3,87E-03	isocitrate dehydrogenase (NADP(+)) 1
IFI16	2,33	4,81E-07	1,01	2,33E-02	interferon gamma inducible protein 16
IFNGR2	0,74	6,81E-03	0,58	2,55E-03	interferon gamma receptor 2
IGF2BP3	0,62	1,65E-02	0,41	4,13E-02	insulin like growth factor 2 mRNA binding protein 3
IL6ST	1,55	6,19E-07	0,74	6,07E-03	interleukin 6 cytokine family signal transduce
ITGAV	1,28	5,60E-05	0,75	2,78E-03	integrin subunit alpha V
ITGB2	0,73	1,56E-02	0,88	4,73E-02	integrin subunit beta 2
KIAA1191	0,58	3,45E-02	0,69	1,29E-05	KIAA1191
KRCC1	1,15	7,21E-04	0,51	8,44E-03	lysine rich coiled-coil 1

LAIR1	0,67	1,68E-02	0,7	6,76E-08	leukocyte associated immunoglobulin like receptor 1
LAPTM4A	1,01	3,15E-04	1,12	9,91E-20	lysosomal protein transmembrane 4 alpha
LCP2	0,77	3,03E-03	0,98	1,97E-05	lymphocyte cytosolic protein 2
LEPROT	1,07	9,59E-04	0,55	6,82E-03	leptin receptor overlapping transcript
LRP1	2,68	5,09E-05	1,64	2,62E-02	LDL receptor related protein 1
MAGEF1	1,01	3,90E-02	0,92	2,79E-02	MAGE family member F1
MANBA	1,07	3,46E-04	0,54	8,99E-04	mannosidase beta
MFSD6	1,44	8,60E-04	0,79	3,21E-02	major facilitator superfamily domain containing 6
MILR1	1,5	2,43E-02	1,08	5,84E-03	mast cell immunoglobulin like receptor 1
MR1	1,06	4,46E-04	0,73	2,81E-04	major histocompatibility complex, class I-related
MYD88	0,73	6,19E-03	0,56	3,87E-02	MYD88 innate immune signal transduction adaptor
MYO1C	0,87	3,02E-02	0,78	5,09E-04	myosin IC
NAGK	0,98	7,35E-03	1,14	5,41E-13	N-acetylglucosamine kinase
NCF1B	1,76	3,37E-03	1,58	2,13E-03	neutrophil cytosolic factor 1B pseudogene
NHLRC3	1	5,07E-04	0,71	2,33E-04	NHL repeat containing 3
NRP1	1,37	3,15E-05	0,69	1,87E-02	neuropilin 1
PAFAH2	0,83	3,32E-02	0,66	4,40E-02	platelet activating factor acetylhydrolase 2
PECAM1	1,64	1,28E-08	1,59	3,85E-11	platelet and endothelial cell adhesion molecule 1
PKN2	0,76	2,34E-02	0,44	4,27E-02	protein kinase N2
POLR3GL	0,73	1,80E-02	0,51	8,89E-03	RNA polymerase III subunit GL
PON2	0,6	1,88E-02	0,36	4,97E-02	paraoxonase 2
PPIC	1,14	1,64E-04	0,71	2,53E-05	peptidylprolyl isomerase C
PRKCA	0,98	1,20E-05	0,52	4,28E-03	protein kinase C alpha
PTTG1IP	0,53	4,49E-02	0,66	2,93E-04	PTTG1 interacting protein
RAB31	1,01	6,42E-04	0,65	3,06E-02	RAB31, member RAS oncogene family
RASSF2	0,65	5,03E-03	0,45	7,52E-03	Ras association domain family member 2
RDX	0,6	4,00E-02	0,53	3,10E-04	radixin
RHOU	1,01	4,92E-04	0,61	7,06E-03	ras homolog family member U
RRAGD	0,54	4,42E-02	0,63	1,57E-03	Ras related GTP binding D
SCARB2	0,62	1,48E-02	0,67	3,40E-02	scavenger receptor class B member 2
SEC23A	0,66	2,67E-02	0,44	2,21E-02	SEC23 homolog A, COPII coat complex component
SERINC3	0,55	2,94E-02	0,42	6,60E-05	serine incorporator 3

SETD7	0,91	1,62E-04	0,89	1,52E-13	SET domain containing 7, histone lysine methyltransferase
SGK3	1,04	6,88E-04	0,9	6,76E-08	serum/glucocorticoid regulated kinase family member 3
SGMS2	1,22	1,37E-02	0,95	4,71E-02	sphingomyelin synthase 2
SIGLEC14	1,35	9,71E-05	0,71	3,38E-02	sialic acid binding Ig like lectin 14
SIGLEC7	0,8	1,85E-02	0,63	1,02E-03	sialic acid binding Ig like lectin 7
SIGLEC9	1,64	3,80E-02	1,68	1,97E-03	sialic acid binding Ig like lectin 9
SKAP2	1,1	3,05E-05	0,57	4,18E-08	src kinase associated phosphoprotein 2
SLC2A6	1,32	3,41E-02	0,87	2,73E-03	solute carrier family 2 member 6
SLC30A1	0,63	3,53E-02	0,55	4,91E-02	solute carrier family 30 member 1
SMPD1	1,24	4,71E-03	1,04	5,92E-04	sphingomyelin phosphodiesterase 1
SNN	0,91	8,84E-03	0,57	2,45E-02	stannin
SNX30	0,83	3,34E-02	0,55	2,33E-03	sorting nexin family member 30
SORT1	1,62	4,92E-04	0,89	1,37E-02	sortilin 1
TGFBR2	1,38	2,07E-07	0,9	2,95E-06	transforming growth factor beta receptor 2
THBD	0,82	2,37E-03	0,95	1,42E-03	thrombomodulin
THEMIS2	0,99	9,04E-04	0,61	2,63E-02	thymocyte selection associated family member 2
TM6SF1	2,07	6,53E-03	1,99	9,11E-04	transmembrane 6 superfamily member 1
TMEM30A	0,55	3,90E-02	0,31	3,40E-02	transmembrane protein 30A
TNFRSF10B	0,92	6,67E-04	0,96	2,39E-06	TNF receptor superfamily member 10b
TWF1	0,78	2,34E-03	0,96	1,06E-07	twinfilin actin binding protein 1
UBA7	1,21	7,43E-03	0,61	2,88E-02	ubiquitin like modifier activating enzyme 7
UBASH3B	1,04	1,34E-04	0,43	3,28E-02	ubiquitin associated and SH3 domain containing B
USP3	0,62	2,70E-02	0,54	5,32E-03	ubiquitin specific peptidase 3
VSIR	0,86	6,62E-03	0,8	4,52E-03	V-set immunoregulatory receptor
WDR11	0,65	2,94E-02	0,27	4,38E-02	WD repeat domain 11
YPEL5	1,4	8,52E-04	0,92	1,20E-02	yippee like 5
ZNF367	0,63	1,37E-02	0,87	1,36E-08	zinc finger protein 367

Appendix Table S2. List of 74 genes commonly downregulated upon HIF2α silencing in HL60 and Kasumi1 cells.

Gene Symbol	HL60 Log_2FC	HL60 padj	Kasumi1 Log_2FC	Kasumi1 padj	Description
ABT1	-0,71	8,97E-03	-0,43	1,26E-02	activator of basal transcription 1
APBA2	-0,86	7,39E-03	-0,51	1,68E-02	amyloid beta precursor protein binding family A member 2
ASB6	-0,62	3,34E-02	-0,35	1,26E-02	ankyrin repeat and SOCS box containing 6
ASIC1	-0,74	4,16E-02	-0,53	4,91E-02	acid sensing ion channel subunit 1
BCL11A	-0,81	4,99E-02	-1,35	2,91E-03	BAF chromatin remodeling complex subunit BCL11A
CALR	-0,68	4,39E-03	-0,23	2,54E-02	calreticulin
CBX6	-1,04	5,47E-06	-0,88	3,89E-18	chromobox 6
CDK6	-0,69	2,70E-02	-0,66	1,78E-03	cyclin dependent kinase 6
CHERP	-0,58	2,93E-02	-0,36	8,19E-04	calcium homeostasis endoplasmic reticulum protein
CLUH	-0,65	6,86E-03	-0,28	9,22E-04	clustered mitochondria homolog
COL27A1	-1,05	1,67E-02	-1,37	1,91E-08	collagen type XXVII alpha 1 chain
CSK	-0,81	1,58E-03	-0,36	4,38E-02	C-terminal Src kinase
DDX54	-0,74	3,52E-03	-0,33	1,31E-02	DEAD-box helicase 54
DHX37	-0,89	3,47E-04	-0,25	2,44E-02	DEAD-box helicase 37
DLX2	-0,94	3,75E-02	-0,85	1,23E-03	distal-less homeobox 2
EIF1AX	-0,55	3,34E-02	-0,5	2,91E-05	eukaryotic translation initiation factor 1A X-linked
FAM216A	-0,66	1,34E-02	-0,6	6,15E-05	family with sequence similarity 216 member A
FBRSL1	-0,89	4,42E-04	-0,57	1,59E-04	fibrosin like 1
FHOD1	-0,55	3,93E-02	-0,36	2,78E-03	formin homology 2 domain containing 1
FLT3	-0,74	3,80E-02	-0,84	1,62E-03	fms related receptor tyrosine kinase 3
GEMIN4	-0,81	6,39E-04	-0,51	1,58E-04	gem nuclear organelle associated protein 4
HDLBP	-0,49	3,78E-02	-0,42	4,50E-09	high density lipoprotein binding protein
HIVEP2	-1,07	3,25E-02	-1	1,78E-02	HIVEP zinc finger 2
HSPB1	-0,93	1,33E-03	-0,32	4,43E-02	heat shock protein family B (small) member 1
IRF2BP1	-0,7	2,86E-02	-0,39	4,47E-02	interferon regulatory factor 2 binding protein 1
KIF21B	-0,66	1,12E-02	-0,49	3,51E-02	kinesin family member 21B

LGR4	-0,83	3,26E-02	-0,75	1,25E-04	leucine rich repeat containing G protein-coupled receptor 4
LMNB2	-0,81	1,22E-03	-0,28	2,91E-03	lamin B2
LPCAT1	-0,59	7,42E-03	-0,36	2,85E-04	lysophosphatidylcholine acyltransferase 1
LRP3	-1,02	1,08E-03	-0,71	4,51E-02	LDL receptor related protein 3
LRRC47	-0,57	3,56E-02	-0,35	2,32E-02	leucine rich repeat containing 47
LZTS2	-0,6	3,80E-02	-0,71	6,44E-06	leucine zipper tumor suppressor 2
MED24	-0,58	2,13E-02	-0,27	4,51E-02	mediator complex subunit 24
MPO	-1,69	1,85E-02	-1,47	9,60E-04	myeloperoxidase
MRM1	-0,79	1,57E-02	-0,51	7,38E-03	mitochondrial rRNA methyltransferase 1
MSRB3	-1,16	5,42E-07	-1,97	9,89E-23	methionine sulfoxide reductase B3
MYBBP1A	-0,66	4,46E-03	-0,24	3,76E-02	MYB binding protein 1a
NFKBIB	-0,71	1,33E-02	-0,36	4,81E-02	NFKB inhibitor beta
NUDT15	-0,75	1,80E-02	-0,82	5,17E-08	nudix hydrolase 15
P3H3	-0,85	2,91E-02	-0,68	3,67E-02	prolyl 3-hydroxylase 3
PPP2R2D	-0,64	2,76E-02	-0,55	2,85E-04	protein phosphatase 2 regulatory subunit Bdelta
PRR22	-1,51	3,67E-02	-0,76	2,02E-02	proline rich 22
PRRT4	-0,98	5,15E-04	-0,89	1,81E-02	proline rich transmembrane protein 4
PSMD3	-0,78	9,36E-04	-0,51	4,11E-07	proteasome 26S subunit, non-ATPase 3
PUS1	-0,74	6,10E-03	-0,38	5,35E-03	pseudouridine synthase 1
RAC3	-1,33	1,44E-04	-0,88	1,36E-07	Rac family small GTPase 3
RANGAP1	-0,65	9,09E-03	-0,2	3,40E-02	Ran GTPase activating protein 1
RASGRP2	-0,67	2,57E-02	-0,52	9,56E-03	RAS guanyl releasing protein 2
REXO4	-0,69	4,02E-03	-0,58	2,70E-08	REX4 homolog, 3'-5' exonuclease
RGS18	-0,76	1,01E-02	-0,79	1,93E-02	regulator of G protein signaling 18
RHOT2	-0,76	3,59E-03	-0,22	4,05E-02	ras homolog family member T2
RNF216P1	-0,86	2,09E-03	-0,74	3,44E-03	ring finger protein 216 pseudogene 1
RP11-498C9.15	-0,89	2,02E-03	-1,18	6,39E-03	pre-mRNA processing factor 31
RPIA	-0,51	3,85E-02	-0,26	4,16E-02	ribose 5-phosphate isomerase A
RUNX2	-0,79	4,90E-01	-1,25	3,52E-06	Runt-Related Transcription Factor 2
SART1	-0,63	2,06E-02	-0,33	3,09E-02	spliceosome associated factor 1, recruiter of U4/U6.U5 tri-snRNP
SERPINB10	-1,14	4,76E-05	-1,66	7,97E-05	serpin family B member 10
SERPINB2	-1,01	5,09E-06	-2,51	1,51E-08	serpin family B member 2

SIRT7	-0,57	3,15E-02	-0,41	6,07E-03	sirtuin 7
SLC7A5	-1,29	1,94E-08	-0,52	2,63E-10	solute carrier family 7 member 5
SMPD4	-0,7	6,42E-03	-0,27	4,65E-02	sphingomyelin phosphodiesterase 4
TBC1D2B	-0,54	4,50E-02	-0,55	2,80E-05	TBC1 domain family member 2B
TCOF1	-0,73	2,43E-03	-0,25	1,58E-02	treacle ribosome biogenesis factor 1
TELO2	-1,07	1,56E-04	-0,4	8,69E-06	telomere maintenance 2
TMTC2	-0,73	3,98E-02	-0,75	1,26E-03	transmembrane O-mannosyltransferase targeting cadherins 2
TNKS1BP1	-0,75	2,89E-02	-0,68	2,67E-04	tankyrase 1 binding protein 1
TONSL	-0,93	1,79E-03	-0,4	4,01E-03	tonsoku like, DNA repair protein
TRIM28	-0,72	2,43E-03	-0,27	5,65E-03	tripartite motif containing 28
TUSC2	-0,54	4,74E-02	-0,46	1,17E-02	tumor suppressor 2, mitochondrial calcium regulator
UHRF1	-0,86	4,77E-03	-0,64	3,04E-12	ubiquitin like with PHD and ring finger domains 1
VIT	-0,65	3,32E-02	-0,77	2,80E-02	vitrin
WDR18	-1,08	4,11E-04	-0,29	1,57E-02	WD repeat domain 18
WDR4	-0,83	5,55E-03	-0,52	2,54E-02	WD repeat domain 4
ZFP37	-1,49	3,56E-02	-1,25	2,96E-02	ZFP37 zinc finger protein

Appendix Table S3. Probes for qPCR.

Gene	Assay ID
EPAS1	Hs01026142_m1
HIF1A	Hs00936371_m1
FLT3	Hs00174690_m1
CDK6	Hs01026371_m1
TRIM28	Hs00232212_m1
BCL11A	Hs01093197_m1
RUNX2	Hs01047973_m1
UHRF1	Hs01086727_m1
PGK1	Hs00943178_g1
PDK1	Hs01561847_m1
ITGAM	Hs00167304_m1
ITGB2	Hs00164957_m1
CD53	Hs00174065_m1
IFI16	Hs00986757_m1
18s	Hs03003631_g1

Appendix Table S4. Sequences of primers for ChIP experiments.

Primers	Sequence (5’-3’)
BCL11A forward	AAAGGTGCGTGCTGTCTCAA
BCL11A reverse	AACCCTCATCCCATCTGGAAC
HIF2α forward	CCCCAACCCTTTCTGTGTAC
HIF2α reverse	GGACCCATGAGTTTCACAGG
RARβ forward	TCCTGGGAGTTGGTGATGTCAG
RARβ reverse	AAACCCTGCTCGGATCGCTC
RUNX2 forward	TCTTTATTGTAAAGGCCGCCA
RUNX2 reverse	CATGAAAGTGTTAGTCTGGGCA
SLC7A5 forward	TGGCATGACAGTAGGGTTGT
SLC7A5 reverse	GAATTTCCAGTACACGCTGCC
