# Supplementary material for: The transcription factor HIF2α partakes in the differentiation block of acute myeloid leukemia
Source: EMBO Mol Med. 2023 Oct 9;15(11):e17810. doi: 10.15252/emmm.202317810 (PMC10630882; doi:10.15252/emmm.202317810)
Supplement: Supplementary file 2 — Expanded View Figures PDF [file EMMM-15-e17810-s005.pdf]

# Expanded View Figures

**Figure EV1. Expression and silencing of HIF1α and HIF2α in AML cell lines.**

A qPCR analysis of HIF1α and HIF2α in the indicated AML cell lines stably expressing shRNAs against HIF1α or HIF2α or a scrambled shRNA as control (shCTRL). Data are expressed as fold change in HIFα-silenced cells compared to shCTRL cells. Data represent mean ± SD of three biological replicates (Student's *t*-test).
B Cell proliferation of the indicated AML cell lines carrying shHIF1α, shHIF2α, or shCTRL. Values represent cell numbers normalized over day 0. Data represent mean ± SD of three biological replicates (Student's *t*-test).
C qPCR analysis of HIF1α and HIF2α in HL60 and Kasumi1 cells stably expressing shCTRL, shHIF1α or shHIF2α. Data are expressed as fold change in HIFα-silenced cells compared to shCTRL cells. Data represent mean ± SD of three biological replicates of cells at passages 4–10 (< P10) or passages 11–16 (> P10) (Student's *t*-test).
D Analysis of *HIF1A* and *EPAS1* (encoding HIF1α and HIF2α respectively) basal expression in the human AML cell lines utilized in this study. Data represent mean ± SD of three biological replicates.
E mRNA expression of *HIF1A* and *EPAS1* in 451 AML patients from the Oregon Health & Science University (OSHU) dataset (Tyner *et al*, 2018). Data are expressed as normalized RPKM (Reads Per Kilobase Million), and were obtained from the cBioportal database (Cerami *et al*, 2012). Data represent mean ± SD of 451 biological replicates.
F Immunoblot analysis showing silencing efficiency of two independent shRNAs against HIF1α (shHIF1α#1 and shHIF1α#2) and HIF2α (shHIF2α#1 and shHIF2α#2), or a scrambled shRNA as control (shCTRL) in Kasumi1 cells. shHIF1α#1 and shHIF2α#2 are shRNAs utilized in the main figures. α-tubulin was used as loading control.
G qPCR analysis of HIF1α and HIF2α upon HIFα-specific silencing and compared to shCTRL in Kasumi1 (left graph) and HL60 (right graph) cells. Data represent mean ± SD of three biological replicate (Student's *t*-test).
H Colony forming capacity of Kasumi1 (left graph) and HL60 (right graph) cells expressing shHIF1α#1, shHIF1α#2, shHIF2α#1, shHIF2α#2, or shCTRL. Shown is the average number of colonies/field in 20 fields (10x objective). Data represent mean ± SD of three biological replicates (Student's *t*-test).
I Percentages of $CD11b^+$ in cells described in (H). Data represent mean ± SD of three biological replicates (Student's *t*-test).

 © 2023 The Authors

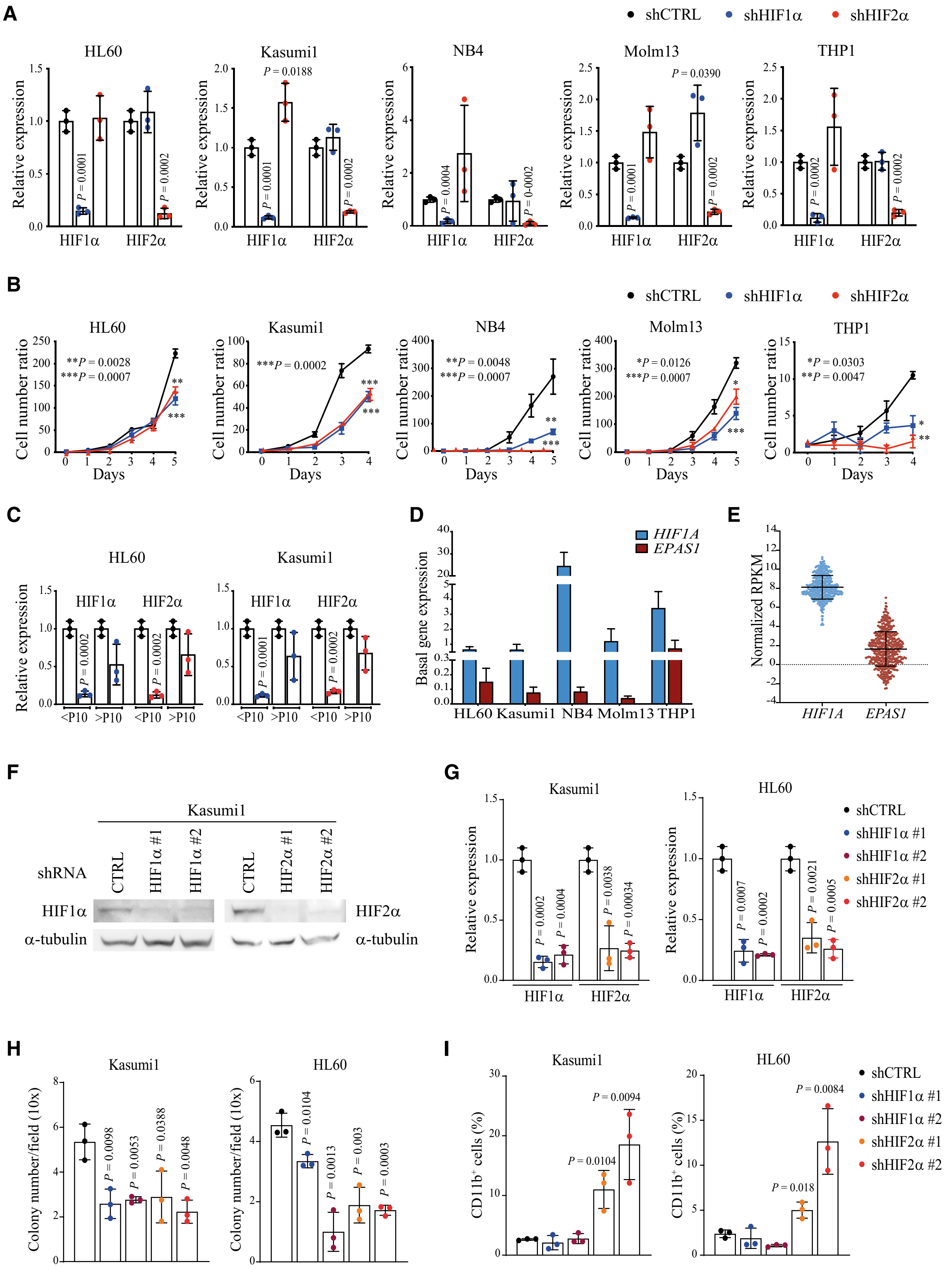
A
shCTRL
shHIF1α
shHIF2α
HL60
Kasumi1
NB4
Molm13
THP1
Relative expression
HIF1α
HIF2α
B
Cell number ratio
Days
C
<P10
>P10
D
HIF1A
EPAS1
Basal gene expression
E
Normalized RPKM
F
Kasumi1
shRNA
CTRL
HIF1α #1
HIF1α #2
HIF2α #1
HIF2α #2
HIF1α
HIF2α
α-tubulin
G
shHIF1α #1
shHIF1α #2
shHIF2α #1
shHIF2α #2
H
Colony number/field (10x)
I
CD11b+ cells (%)

**Figure EV1.**

**Figure EV2. Global changes in gene expression, H3K27me3 and chromatin accessibility upon HIF1α or HIF2α silencing in AML cells.**

A Unsupervised hierarchical clustering of differentially expressed genes (DEGs) in HL60 and Kasumi1 cells following HIF1α or HIF2α silencing. A red-blue color scale was used to reflect normalized RPKM, with red indicating genes with higher expression and blue indicating genes with lower expression. Each column represents the average of two independent experiments.

B Venn diagrams showing the number of DEGs commonly upregulated and downregulated in HL60 and Kasumi1 cells upon shHIF1α (top) or shHIF2α (bottom). Common/total deregulated genes in each cell line are indicated as percentages.

C qPCR analysis of the indicated HIF2α-regulated genes in NB4 (left graph), Molm13 (middle graph) and THP1 (right graph) cells upon HIF2α silencing. Values indicate fold change in gene expression compared to shCTRL cells. Data represent mean ± SD of three biological replicates (Student's *t*-test).

D, E Gene set enrichment analysis of genes upregulated upon HIF2α silencing and showing unique H3K27me3 peaks in shCTRL (D) or downregulated upon HIF2α silencing and showing unique H3K27me3 peaks in shHIF2α (E) Kasumi1 cells. Indicated are the terms most significantly enriched in the following libraries: gene ontology (GO) biological process, GO molecular function, GO cellular component, Bioplanet, Reactome, and Hallmarks of cancer. Dot sizes represent the number of genes in each term, and colors indicate Enrichment Scores expressed as $-\log_{10}$ (*P*-value).

F Genome browser view of normalized counts of ATAC-seq profiles at the regulatory regions of the myeloid differentiation genes *ITGB2*, *CD53*, and *IFI16*. Open chromatin peaks of Kasumi1 cells are indicated in red for shCTRL and green for shHIF2α. Triplicate experiments and a representative alignment (normalized CPM values) of each condition are shown. Open chromatin peaks of six primary AML cells (obtained from GSE197416; Data ref: Gambacorta *et al*, 2022b) are indicated in orange and a representative alignment is shown. Lower panels represent the corresponding gene annotation from GrCh38 human reference.

 © 2023 The Authors

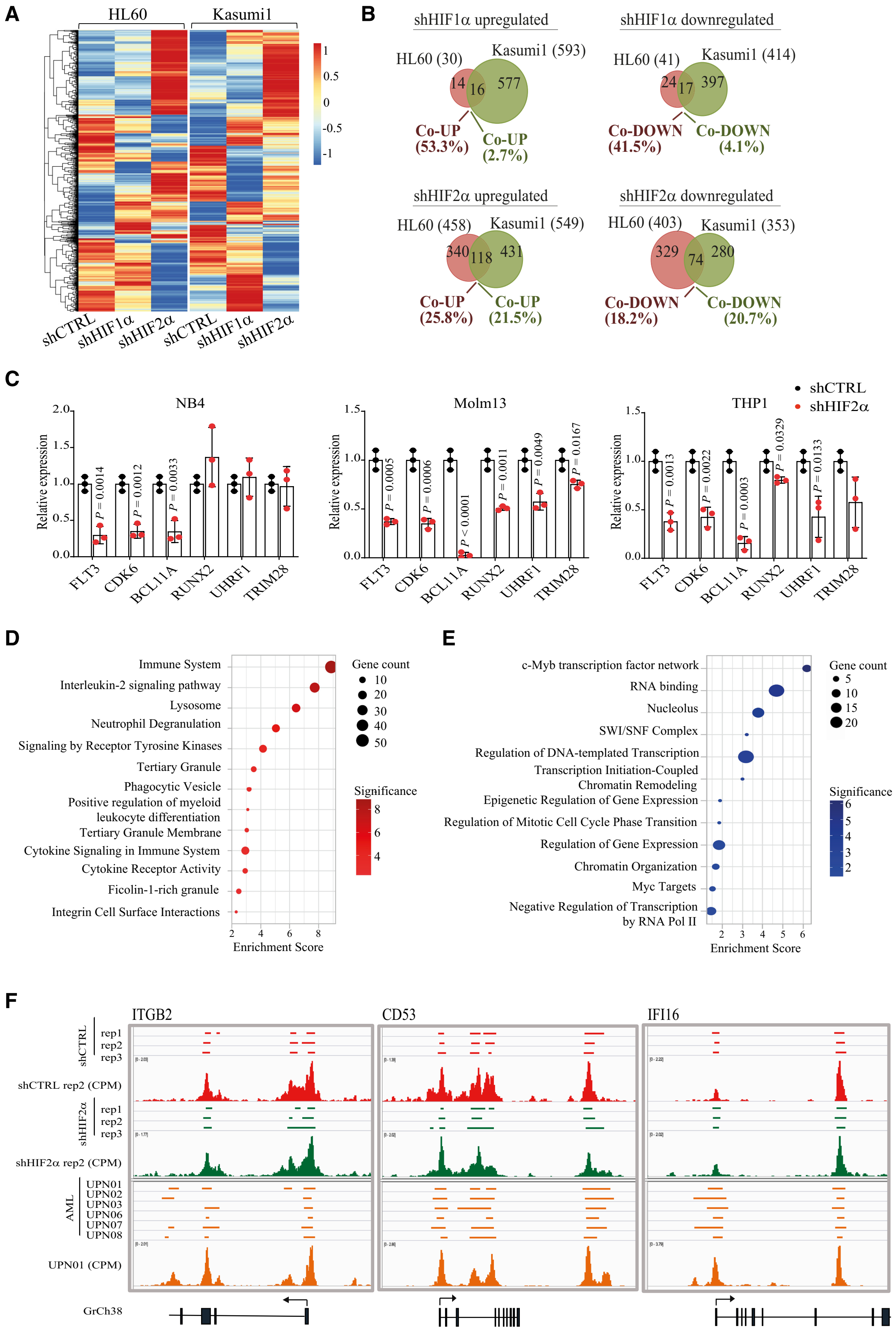
A
HL60
Kasumi1
shCTRL
shHIF1α
shHIF2α
shCTRL
shHIF1α
shHIF2α
B
shHIF1α upregulated
HL60 (30)
Kasumi1 (593)
Co-UP (53.3%)
Co-UP (2.7%)
shHIF1α downregulated
HL60 (41)
Kasumi1 (414)
Co-DOWN (41.5%)
Co-DOWN (4.1%)
shHIF2α upregulated
HL60 (458)
Kasumi1 (549)
Co-UP (25.8%)
Co-UP (21.5%)
shHIF2α downregulated
HL60 (403)
Kasumi1 (353)
Co-DOWN (18.2%)
Co-DOWN (20.7%)
C
NB4
Molm13
THP1
shCTRL
shHIF2α
Relative expression
FLT3
CDK6
BCL11A
RUNX2
UHRF1
TRIM28
D
Immune System
Interleukin-2 signaling pathway
Lysosome
Neutrophil Degranulation
Signaling by Receptor Tyrosine Kinases
Tertiary Granule
Phagocytic Vesicle
Positive regulation of myeloid leukocyte differentiation
Tertiary Granule Membrane
Cytokine Signaling in Immune System
Cytokine Receptor Activity
Ficolin-1-rich granule
Integrin Cell Surface Interactions
Gene count
Significance
Enrichment Score
E
c-Myb transcription factor network
RNA binding
Nucleolus
SWI/SNF Complex
Regulation of DNA-templated Transcription
Transcription Initiation-Coupled Chromatin Remodeling
Epigenetic Regulation of Gene Expression
Regulation of Mitotic Cell Cycle Phase Transition
Regulation of Gene Expression
Chromatin Organization
Myc Targets
Negative Regulation of Transcription by RNA Pol II
Gene count
Significance
Enrichment Score
F
ITGB2
CD53
IFI16
shCTRL
rep1
rep2
rep3
shCTRL rep2 (CPM)
shHIF2α
shHIF2α rep2 (CPM)
AML
UPN01
UPN02
UPN03
UPN06
UPN07
UPN08
UPN01 (CPM)
GrCh38

**Figure EV2.**

**Figure EV3. *In vitro* and *ex vivo* treatment of AML cell lines and PDX-derived cells with the specific HIF2α inhibitor PT2385.**

A Cell numbers of NB4, Molm13 and THP1 cells 2 days after treatment with PT2385 at the indicated doses and compared to vehicle treated cells. Data represent mean ± SD of three biological replicates (one-way ANOVA followed by Tukey's multiple comparison test).

B Percentages of $CD11b^+$ NB4, Molm13 and THP1 cells 2 days after treatment with PT2385 at the indicated doses and compared to vehicle treated cells. Data represent mean ± SD of three biological replicates (one-way ANOVA followed by Tukey's multiple comparison test).

C Relative proportion of live ($AnnexinV^-$) and apoptotic ($AnnexinV^+$) cells after treatment with PT2385 at the indicated doses for 2 and 6 days in HL60 (upper graphs) and Kasumi1 (lower graphs) cells. Data represent mean ± SD of three biological replicates (Student's *t*-test).

D qPCR analysis of the indicated genes in NB4 (upper graph), Molm13 (middle graph) and THP1 (lower graph) cells 2 days after treatment with 200 μM PT2385. Values are represented as fold change in gene expression compared to vehicle treated cells. Data represent mean ± SD of three biological replicates (Student's *t*-test).

E Percentages of $CD11b^+$ NB4 (upper graph), Molm13 (middle graph) and THP1 (lower graph) cells at the indicated days (D2, D4, D6) after treatment with 50 μM PT2385 and compared to vehicle treated cells. Data represent mean ± SD of three biological replicates (Student's *t*-test).

F Percentages of $CD11b^+$ AML-01 cells isolated from the bone marrow of PDX mice and treated *ex vivo* with 100 μM PT2385 for 4 days. Data represent mean ± SD of three biological replicates (Student's *t*-test).

G qPCR analysis of the indicated genes in AML-01 cells upon *ex vivo* treatment with 100 μM PT2385. Values are represented as fold changes in gene expression compared to control vehicle treated cells. Data represent mean ± SD of three biological replicates (Student's *t*-test).

 © 2023 The Authors

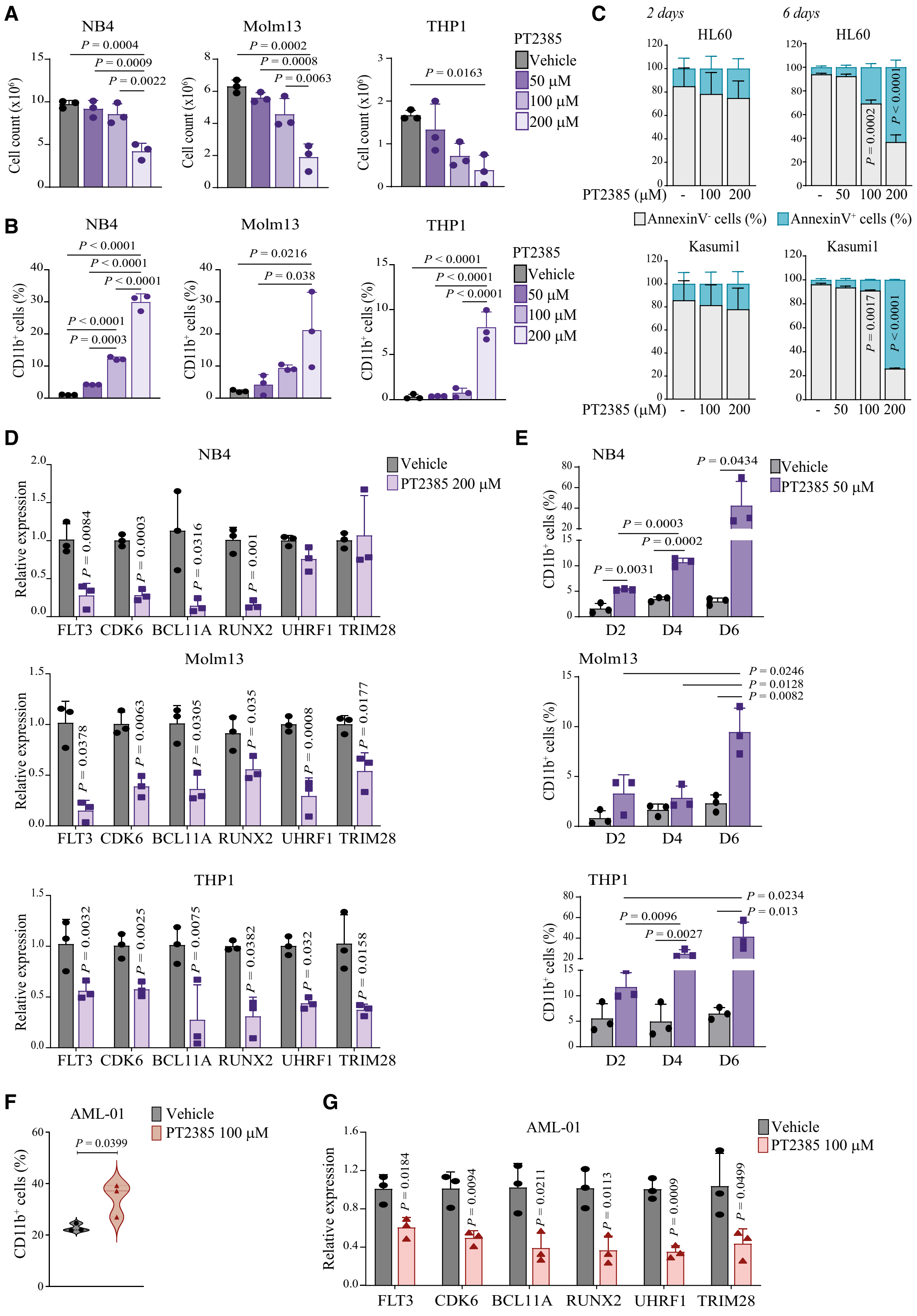
A
NB4
Molm13
THP1
Cell count (x10⁶)
PT2385
Vehicle
50 µM
100 µM
200 µM
B
CD11b⁺ cells (%)
C
2 days
6 days
HL60
Kasumi1
PT2385 (µM)
AnnexinV⁻ cells (%)
AnnexinV⁺ cells (%)
D
Relative expression
FLT3 CDK6 BCL11A RUNX2 UHRF1 TRIM28
PT2385 200 µM
E
PT2385 50 µM
D2 D4 D6
F
AML-01
PT2385 100 µM
G

**Figure EV3.**

**Figure EV4. *In vivo* treatment of PDX AML models with compounds inhibiting HIF2α.**

A Percentages of leukemic cells (hCD45$^+$) expressing AnnexinV in bone marrow (BM, left graph), spleen (SP, middle graph) and peripheral blood (PB, right graph) of mice transplanted with AML-01 cells and treated with 100 mg/kg PT2385 or vehicle. Data represent mean ± SD of four biological replicates.

B qPCR analysis of HIF2α and HIF2α-regulated genes in AML-01 cells isolated from the bone marrow of leukemic mice treated with 100 mg/kg PT2385 ($n$ = 4) or vehicle ($n$ = 3). Values indicate fold change in gene expression compared to control vehicle treated cells and represent mean ± SD of 3/4 biological replicates (Student's $t$-test).

C Percentages of apoptotic leukemic cells (AnnexinV$^+$hCD45$^+$) in bone marrow (BM), spleen (SP) and peripheral blood (PB) of mice transplanted with AML-01 (left graph) or AML-02 (right graph) and treated with EZN-2208. Data are represented in box and whisker plots where the central band denotes the median value, box contains interquartile ranges, while whiskers mark minimum and maximum values. All biological replicates are shown ($n$ = 7 in AML-01, $n$ = 4 in AML-02).

D Spleen weight (left graph) and percentages of leukemic cells (hCD45$^+$, right graph) in BM, SP, and PB of mice transplanted with AML-01 and treated with EZN-2208 as in (C). Data are represented in box and whisker plots where the central band denotes the median value, box contains interquartile ranges, while whiskers mark minimum and maximum values. All biological replicates are shown ($n$ = 7, Student's $t$-test).

E Spleen weight (left graph) and percentages of leukemic cells (hCD45$^+$, right graph) in BM, SP, and PB of mice transplanted with AML-02 and treated with EZN-2208 as in (C). Data are represented in box and whisker plots where the central band denotes the median value, box contains interquartile ranges, while whiskers mark minimum and maximum values. All biological replicates are shown ($n$ = 7, Student's $t$-test).

F Percentages of leukemic AML-01 and AML-02 cells expressing CD11b (hCD11b$^+$hCD45$^+$) in BM, SP, and PB of mice transplanted with AML-01 (left graph) and AML-02 (right graph) and treated with EZN-2208. Data are represented in box and whisker plots where the central band denotes the median value, box contains interquartile ranges, while whiskers mark minimum and maximum values. All biological replicates are shown ($n$ = 7 in AML-01, $n$ = 4 in AML-02, Student's $t$-test).

G qPCR of the indicated genes in cells isolated from BM of vehicle ($n$ = 3) and EZN-2208 treated ($n$ = 4) AML-01 transplanted mice. Values indicate fold changes in gene expression of EZN-2208 treated cells compared to vehicle treated control cells. Data represent mean ± SD of 3/4 biological replicates (Student's $t$-test).


© 2023 The Authors

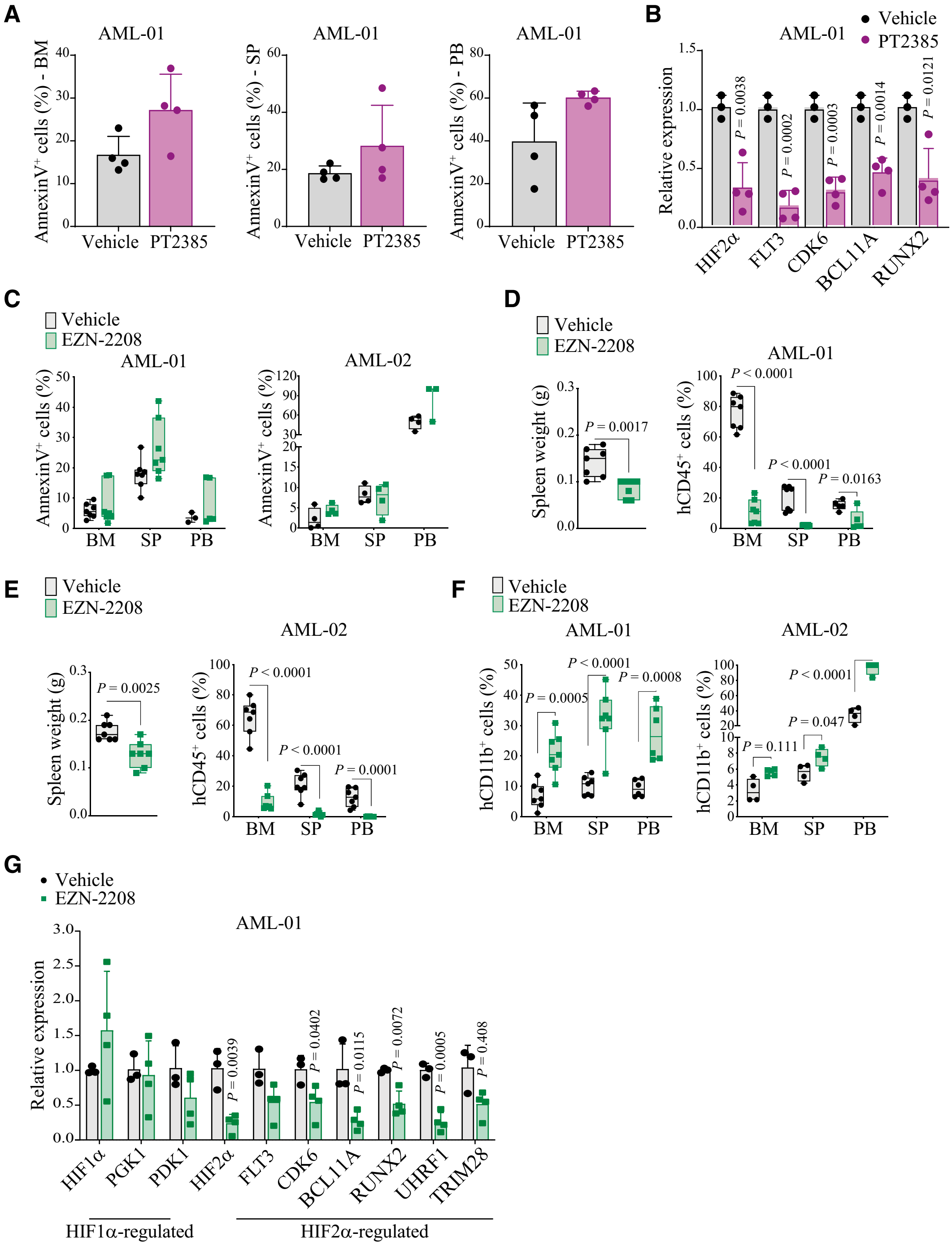

A
AML-01
AnnexinV+ cells (%) - BM
AnnexinV+ cells (%) - SP
AnnexinV+ cells (%) - PB
Vehicle
PT2385
B
AML-01
Relative expression
HIF2α
FLT3
CDK6
BCL11A
RUNX2
P = 0.0038
P = 0.0002
P = 0.0003
P = 0.0014
P = 0.0121
C
Vehicle
EZN-2208
AML-02
BM
SP
PB
D
Spleen weight (g)
P = 0.0017
hCD45+ cells (%)
P < 0.0001
P = 0.0163
E
P = 0.0025
P = 0.0001
F
hCD11b+ cells (%)
P = 0.0005
P = 0.0008
P = 0.047
P = 0.111
G
HIF1α
PGK1
PDK1
UHRF1
TRIM28
P = 0.0039
P = 0.0402
P = 0.0115
P = 0.0072
P = 0.0005
P = 0.408
HIF1α-regulated
HIF2α-regulated


**Figure EV4.**

**Figure EV5. Combination of HIF2α inhibition and ATRA increases AML differentiation in NB4, Molm13, and THP1 cells.**

A Percentages of $CD11b^+$ NB4 (left graph), Molm13 (middle graph) and THP1 (right graph) cells with shRNAs against HIF1α, HIF2α, or a scrambled shRNA as control (shCTRL) treated with 1 μM ATRA for 2 days. Data represent mean ± SD of three biological replicates (one-way ANOVA followed by Tukey's multiple comparison test).

B Percentages of $CD11b^+$ NB4 (left graph), Molm13 (middle graph) and THP1 (right graph) cells following treatment with 50 μM PT2385, 1 μM ATRA, or combination for 4 days. Data represent mean ± SD of three biological replicates (one-way ANOVA followed by Tukey's multiple comparison test).

C Percentages of NB4 (left graph), Molm13 (right graph) and THP1 (lower graph) cells in the indicated phases of the cell cycle following treatment with 50 μM PT2385, 1 μM ATRA, or combination for 4 days. Data represent mean ± SD of three biological replicates (Student's *t*-test).


© 2023 The Authors

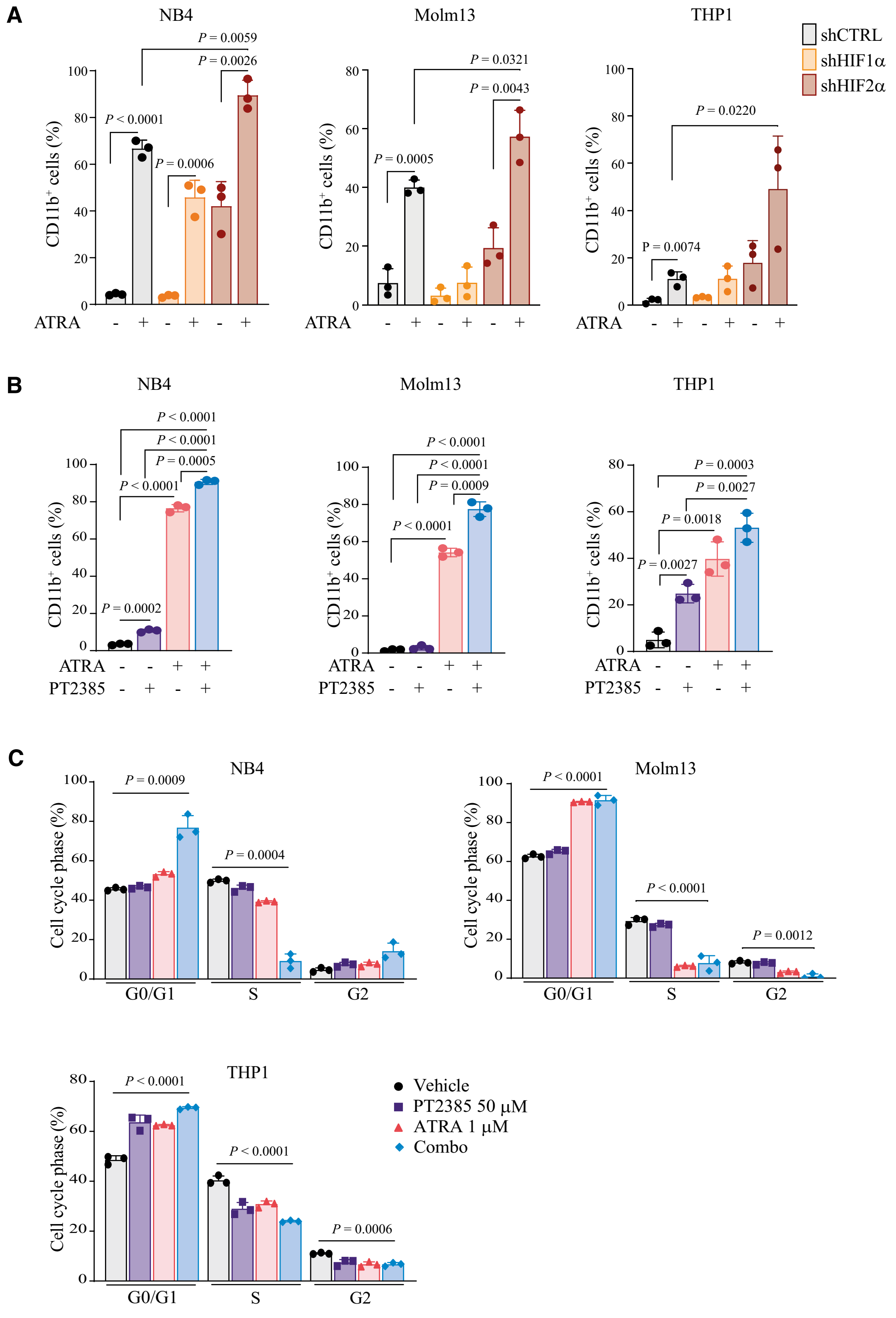
A
NB4
Molm13
THP1
shCTRL
shHIF1α
shHIF2α
CD11b+ cells (%)
ATRA
P < 0.0001
P = 0.0006
P = 0.0059
P = 0.0026
P = 0.0005
P = 0.0321
P = 0.0043
P = 0.0074
P = 0.0220
B
NB4
Molm13
THP1
ATRA
PT2385
P = 0.0002
P < 0.0001
P = 0.0005
P = 0.0009
P = 0.0003
P = 0.0027
P = 0.0018
C
NB4
Molm13
THP1
Cell cycle phase (%)
G0/G1
S
G2
P = 0.0009
P = 0.0004
P < 0.0001
P = 0.0012
P = 0.0006
Vehicle
PT2385 50 µM
ATRA 1 µM
Combo

**Figure EV5.**
