# Supplementary material for: The transcription factor HIF2α partakes in the differentiation block of acute myeloid leukemia
Source: EMBO Mol Med. 2023 Oct 9;15(11):e17810. doi: 10.15252/emmm.202317810 (PMC10630882; doi:10.15252/emmm.202317810)
Supplement: Supplementary file 3 — PDF+ [file EMMM-15-e17810-s009.pdf]

Article

SOURCE DATA | TRANSPARENT PROCESS | OPEN ACCESS

EMBO Molecular Medicine

The transcription factor HIF2α partakes in the differentiation block of acute myeloid leukemia

Daniela Magliulo[1], Matilde Simoni[1], Carolina Caserta[2], Cristina Fracassi[1], Serena Belluschi[3,†], Kety Giannetti[2], Raffaella Pini[4], Ettore Zapparoli[4], Stefano Beretta[2], Martina Uggè[1], Eleonora Draghi[5], Federico Rossari[2,3], Nadia Coltella[2], Cristina Tresoldi[6], Marco J Morelli[4], Raffaella Di Micco[2], Bernhard Gentner[2,‡], Luca Vago[5] & Rosa Bernardi[1,*]

Abstract

One of the defining features of acute myeloid leukemia (AML) is an arrest of myeloid differentiation whose molecular determinants are still poorly defined. Pharmacological removal of the differentiation block contributes to the cure of acute promyelocytic leukemia (APL) in the absence of cytotoxic chemotherapy, but this approach has not yet been translated to non-APL AMLs. Here, by investigating the function of hypoxia-inducible transcription factors HIF1α and HIF2α, we found that both genes exert oncogenic functions in AML and that HIF2α is a novel regulator of the AML differentiation block. Mechanistically, we found that HIF2α promotes the expression of transcriptional repressors that have been implicated in suppressing AML myeloid differentiation programs. Importantly, we positioned HIF2α under direct transcriptional control by the prodifferentiation agent all-*trans* retinoic acid (ATRA) and demonstrated that HIF2α blockade cooperates with ATRA to trigger AML cell differentiation. In conclusion, we propose that HIF2α inhibition may open new therapeutic avenues for AML treatment by licensing blasts maturation and leukemia debulking.

Keywords AML; ATRA; differentiation therapy; HIF2α
Subject Categories Cancer; Haematology
DOI 10.15252/emmm.202317810 | Received 5 April 2023 | Revised 19 September 2023 | Accepted 21 September 2023 | Published online 9 October 2023
EMBO Mol Med (2023) 15: e17810

Introduction

Acute myeloid leukemia (AML) is an aggressive disease characterized by uncontrolled proliferation and arrest of myeloid differentiation. AML is genetically heterogeneous, with different karyotypic aberrations, mutations, gene expression, and epigenetic profiles that define disease subsets and clonal populations within individual patients (Döhner *et al*, 2017). The main therapeutic opportunity for AML patients consists of intensive chemotherapy and allogeneic hematopoietic cell transplantation for eligible candidates, with the recent introduction of novel targeted therapies for selected groups of patients. However, elderly patients cannot sustain overly toxic treatments, and younger patients who undergo remission upon standard therapies often relapse due to genetic plasticity of clonal AML populations and therapy-resistant leukemia stem cells (LSCs). For these reasons, AML survival is still discouragingly low, and new therapeutic options are urgently needed (Döhner *et al*, 2017).

Blockade of myeloid differentiation is a common feature of AML, occurring at different stages of myeloid maturation, and generating morphological subsets that are only partly defined by genetic features. In some AML subsets, oncogenic drivers impose a block of differentiation by directly perturbing the expression of lineage commitment genes, as is the case of the PML-RARα fusion protein of acute promyelocytic leukemia (APL) (van Gils *et al*, 2017). In other instances, oncogenic transcriptional regulators affect expression of differentiation genes via epigenetic mechanisms (e.g., IDH or TET mutants; Figueroa *et al*, 2010). However, in most cases, the molecular underpinnings of the differentiation block remain to be elucidated, and it is not known if common regulatory mechanisms may exist across AML subsets.

Defining the details of halted differentiation is crucial not only to gain insights into AML pathogenesis but also to transform this feature into an actionable vulnerability. In this respect, the finding that all-*trans* retinoic acid (ATRA) triggers differentiation of APL blasts has been a turning point in AML therapy and has sparked considerable interest into translating ATRA treatment to other AML subsets. However, few non-APL AML subtypes undergo differentiation upon ATRA treatment (El Hajj *et al*, 2015; Ma *et al*, 2016; Verhagen

1 Division of Experimental Oncology, IRCCS San Raffaele Scientific Institute, Milan, Italy
2 San Raffaele Telethon Institute for Gene Therapy (SR-TIGET), IRCCS San Raffaele Scientific Institute, Milan, Italy
3 Vita Salute San Raffaele University School of Medicine, Milan, Italy
4 Center for Omics Sciences, IRCCS San Raffaele Scientific Institute, Milan, Italy
5 Unit of Immunogenetics, Leukemia Genomics and Immunobiology, IRCCS San Raffaele Scientific Institute, Milan, Italy
6 Unit of Hematology and Bone Marrow Transplantation, IRCCS San Raffaele Scientific Institute, Milan, Italy
*Corresponding author. Tel: +39 0226 435606; Fax: +39 0226 435602; E-mail: bernardi.rosa@hsr.it
†Present address: Mogrify, Cambridge, UK
‡Present address: Ludwig Institute for Cancer research, Lausanne University, Lausanne, Switzerland

© 2023 The Authors. Published under the terms of the CC BY 4.0 license.

et al, 2016; Mugoni *et al*, 2019), and it is hypothesized that most AMLs are resistant to ATRA-induced differentiation because of epigenetic silencing of myeloid differentiation genes (van Gils *et al*, 2017).

In this work, we identify the transcription factor HIF2α as a novel regulator of the AML differentiation block. Hypoxia-inducible factors (HIFs) are heterodimeric transcription factors composed of an inducible α and a constitutive β subunit. The two main α subunits, HIF1α and HIF2α, perform nonredundant functions and regulate different and cell type-specific target genes (Magliulo & Bernardi, 2018). The function of HIF factors has been widely studied in solid tumors, where they promote tumor progression by regulating cell metabolism, neo-angiogenesis, metastasis, and stem cell features (Wigerup *et al*, 2016). In AML, recent work has described HIF1α and HIF2α as either tumor promoters (Wang *et al*, 2011; Matsunaga *et al*, 2012; Rouault-Pierre *et al*, 2013; Coltella *et al*, 2014; Forristal *et al*, 2015; Gao *et al*, 2015; Migliavacca *et al*, 2016) or tumor suppressors (Velasco-Hernandez *et al*, 2014, 2019; Vukovic *et al*, 2015), a distinction that may be dictated by molecular specificities of leukemia subtypes, or different outputs of HIFs activity in normal hematopoietic progenitors versus leukemic cells (Magliulo & Bernardi, 2018).

Here, by comparing the activity of HIF1α and HIF2α in models of established AML, we confirmed that both play oncogenic functions, and uncovered a new role of HIF2α in hindering AML differentiation. Also, we found that HIF2α inhibition cooperates with ATRA to favor AML maturation. This finding has attractive therapeutic implications, as a small molecule inhibitor of HIF2α has been recently approved for patients with von-Hippel Lindau disease and is in clinical testing for other tumor types (Wallace *et al*, 2016; Courtney *et al*, 2018; Renfrow *et al*, 2018; Hasanov & Jonasch, 2021). Thus, we propose that HIF2α inhibition may be exploited as a new therapeutic avenue to treat AML.

Results

HIF2α is a novel regulator of AML differentiation

To comparatively define the roles of HIF1α and HIF2α in AML, we interfered with their expression in cell lines representative of favorable (Kasumi1 and NB4 cells) or high-risk (Molm13 and THP1 cells) patients' categories, including cell lines conventionally used for differentiation studies (HL60 and NB4 cells). In accordance with previous observations (Kocabas *et al*, 2012; Schulz *et al*, 2012), silencing of either HIFα factor caused variable compensatory upregulation of the cognate gene (Figs 1A and EV1A). Phenotypically, we found that both HIFα factors promote proliferation and colony formation in all AML cell lines (Figs EV1B and 1B), confirming previous results that described their oncogenic function in established leukemia models (Wang *et al*, 2011; Rouault-Pierre *et al*, 2013; Coltella *et al*, 2014; Forristal *et al*, 2015; Gao *et al*, 2015; Migliavacca *et al*, 2016). Of note, efficacy of HIFα silencing was reduced upon cell passaging (Fig EV1C), suggesting that cells with higher shRNA were being counter selected in the population due to their decreased proliferation. For this reason, all experiments were performed in the first 10 passages upon shRNAs transduction. Thus, although *EPAS1* (HIF2α gene) is expressed at lower levels than *HIF1A* in AML cell lines and patients (Fig EV1D and E), we confirmed that tampering HIF2α expression has important phenotypic consequences in AML, as previously observed by us and others (Rouault-Pierre *et al*, 2013; Coltella *et al*, 2014).

Intriguingly, for the first time, we observed that specific silencing of HIF2α promotes AML differentiation, as measured by surface expression of the myeloid differentiation marker CD11b (Fig 1C, Appendix Fig S1) and morphological changes of maturing myeloid cells such as nuclear multilobulation and reduced nucleus/cytoplasm ratio (Fig 1D). On note, the increase in CD11b$^+$ cells upon HIF2α silencing was variable and more modest in non-APL cell lines than in NB4 cells.

These results were confirmed with an additional set of short hairpin RNAs (shRNAs). Similar levels of HIF1α and HIF2α downregulation led to comparable inhibition of colony formation, while CD11b expression was induced only by HIF2α-specific silencing in both Kasumi1 and HL60 cells (Fig EV1F–I).

Taken together, these data confirm that both HIFα factors promote AML expansion and suggest that HIF2α is specifically involved in the AML differentiation block.

Interestingly, analysis of HIFα expression along normal hematopoiesis revealed that *EPAS1* is predominantly expressed in hematopoietic stem and progenitor cells (HSPCs), with minimal expression in differentiated lineages, while *HIF1A* is also expressed in differentiated cells like monocytes and dendritic cells (Fig 1E), indicating that in hematopoiesis HIF2α functions are preferentially exerted in uncommitted progenitors.

Figure 1. HIF2α silencing induces AML differentiation.

A Immunoblot analysis showing silencing efficiency of shRNAs against HIF1α, HIF2α, or a scrambled shRNA as control (shCTRL) in five AML cell lines at early passages upon retroviral infection (P5-10). α-tubulin was used as loading control. Densitometric analyses in the bottom boxes show relative levels of HIFα factors over control cells. Data are representative of one out of three independent experiments.

B Colony forming capacity of indicated AML cell lines expressing shHIF1α, shHIF2α, or shCTRL. Shown is the average number of colonies/field in 20 fields (10× objective). Data represent mean ± SD of four biological replicates (Student's *t*-test).

C Upper panel: Percentages of AML cells expressing the myeloid differentiation marker CD11b upon HIFα-specific silencing in the indicated cell lines. Lower panel: mean fluorescence intensity (MFI) of CD11b in the indicated cell lines. Data represent mean ± SD of four biological replicates (Student's *t*-test).

D May-Grunwald Giemsa staining of shCTRL and shHIF2α Kasumi1 and NB4 cells. Scale bar, 20 μm (40× objective). Data are representative of one out of three independent experiments. Dot plots on the right indicate nucleus/cytoplasm ratio of shCTRL and shHIF2α Kasumi1 and NB4 cells, with each dot representing a single cell (n = 30, mean ± SD, Student's *t*-test). Areas of nucleus and cytoplasm were calculated using ImageJ software.

E Hierarchical hematopoietic trees showing expression of *HIF1A* and *EPAS1* genes in normal human hematopoiesis using HemaExplorer dataset (data obtained from BloodSpot; Bagger *et al*, 2016).

Source data are available online for this figure.

 © 2023 The Authors

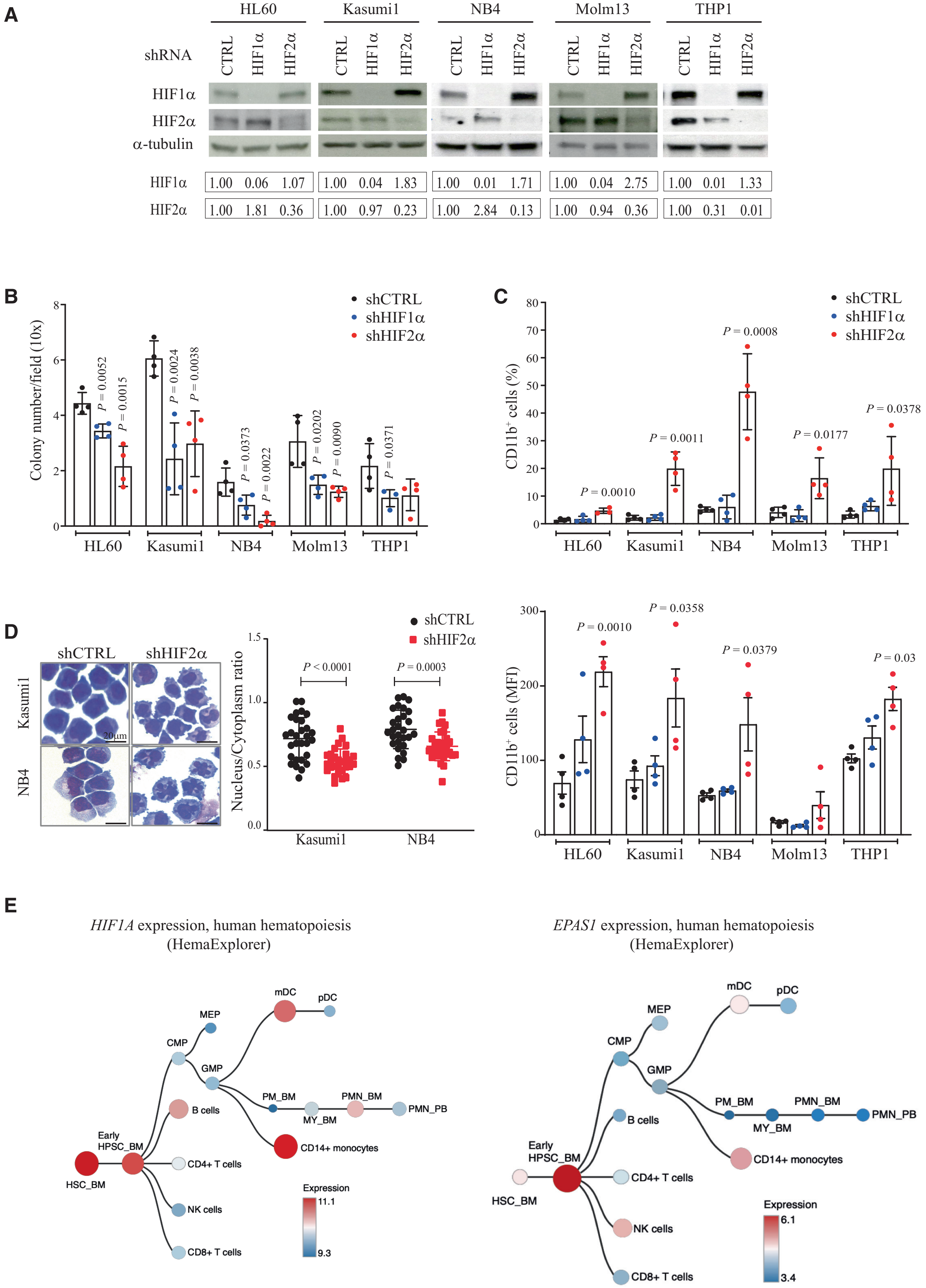
A
HL60
Kasumi1
NB4
Molm13
THP1
shRNA
CTRL HIF1α HIF2α
HIF1α
HIF2α
α-tubulin
HIF1α 1.00 0.06 1.07 | 1.00 0.04 1.83 | 1.00 0.01 1.71 | 1.00 0.04 2.75 | 1.00 0.01 1.33
HIF2α 1.00 1.81 0.36 | 1.00 0.97 0.23 | 1.00 2.84 0.13 | 1.00 0.94 0.36 | 1.00 0.31 0.01
B
Colony number/field (10x)
P = 0.0052
P = 0.0015
P = 0.0024
P = 0.0038
P = 0.0373
P = 0.0022
P = 0.0202
P = 0.0090
P = 0.0371
shCTRL
shHIF1α
shHIF2α
C
CD11b+ cells (%)
P = 0.0010
P = 0.0011
P = 0.0008
P = 0.0177
P = 0.0378
D
shCTRL
shHIF2α
Kasumi1
NB4
20µm
Nucleus/Cytoplasm ratio
P < 0.0001
P = 0.0003
CD11b+ cells (MFI)
P = 0.0010
P = 0.0358
P = 0.0379
P = 0.03
E
HIF1A expression, human hematopoiesis (HemaExplorer)
EPAS1 expression, human hematopoiesis (HemaExplorer)
HSC_BM
Early HPSC_BM
CMP
MEP
GMP
mDC
pDC
PM_BM
MY_BM
PMN_BM
PMN_PB
CD14+ monocytes
B cells
CD4+ T cells
NK cells
CD8+ T cells
Expression
11.1
9.3
6.1
3.4

Figure 1.

HIF2α promotes expression of AML pathogenic genes and represses gene sets of myeloid differentiation

To get mechanistic insights into HIFα functions in AML, we profiled HIFα-regulated transcriptomes in HL60 and Kasumi1 cells. Global changes in gene expression revealed that HIFα factors regulate different genes (Fig EV2A), confirming previous evidence of non-redundant functions (Dengler *et al*, 2014). Principal component analysis on differentially expressed genes showed that HIF2α inhibition causes greater separation from control cells than HIF1α (Appendix Fig S2A), further indicating that albeit expressed at lower levels, HIF2α is an important transcriptional regulator in AML. Analysis of specific gene perturbations revealed that HIF1α silencing produced different degrees of gene deregulation in HL60 and Kasumi1 cells, with HL60 cells responding minimally to HIF1α suppression (Appendix Fig S2B). Consequently, few genes were commonly modulated in the two cell lines (Fig EV2B). Individual functional enrichment analysis (Appendix Fig S2C) showed that HIF1α silencing predominantly perturbed the expression of glycolytic/metabolic pathways, thus confirming previously defined metabolic functions in hematopoiesis and AML (Wierenga *et al*, 2014, 2019).

Silencing of HIF2α caused larger gene expression perturbations (Appendix Fig S2B), with 20% of deregulated genes common to the two AML cell lines (Fig EV2B). To identify shared functions of HIF2α, we focused on gene sets concordantly regulated in HL60 and Kasumi1 cells. Functional enrichment analysis of 118 genes co-upregulated after HIF2α depletion (Appendix Table S1) revealed that the most significant categories are centered on neutrophil maturation and activation (Fig 2A). Shared in these categories are integrins (*ITGB2* and *ITGAV*) and other genes involved in myeloid differentiation (e.g., *CD53*, *IFI16*, *MYD88*, and *CD4*) (Fig 2B), which are induced upon HIF2α and not HIF1α silencing, thus confirming morphological and immunophenotypical differentiation features shown in Fig 1.

In search of HIF2α target genes that may explain the phenotypic consequences of its inhibition, functional enrichment analysis was performed on the 74 genes co-downregulated upon HIF2α silencing (Appendix Table S2). Interestingly, the most significant gene categories are implicated in transcriptional regulation and include epigenetic regulators and chromatin organizers (Fig 2C). Regulation of these gene sets by HIF2α appears specific to AML cells as this was not observed in renal cancer, hematopoiesis, or a chronic myeloid leukemia (CML) cell line (Wierenga *et al*, 2014, 2019; Courtney *et al*, 2020). HIF2α-regulated genes comprehend known inducers of AML pathogenesis and proliferation (*FLT3*, *CDK6*, *BCL11A*, and *RUNX2*) (Gilliland & Griffin, 2002; Kuo *et al*, 2009; Scheicher *et al*, 2015; Sunami *et al*, 2022) and epigenetic regulators involved in cell fate determination and differentiation via heterochromatin formation (*TRIM28* and *UHRF1*; Czerwińska *et al*, 2017; Oleksiewicz *et al*, 2017; Zhao *et al*, 2017; Kim *et al*, 2018; Fig 2D). qPCR analysis confirmed HIF2α-mediated regulation of representative genes in additional AML cell lines, with the exception of NB4 cells where *RUNX2*, *TRIM28*, and *UHRF1* were not regulated upon HIF2α silencing (Fig EV2C).

Enrichment of heterochromatin factors within genes induced by HIF2α is in line with a recently described function of HIF2α in modulating heterochromatin via EZH2 recruitment and H3K27me3-mediated epigenetic silencing of specific target genes in macrophages (Li *et al*, 2021). Because it is generally assumed that the AML differentiation block is caused by epigenetic silencing of myeloid differentiation genes (Momparler *et al*, 2020), we measured H3K27me3 deposition and chromatin accessibility upon HIF2α silencing. HIF2α downregulation affected global H3K27me3 deposition (measured as number, coverage, and intensity of H3K27me3

Figure 2. HIF2α suppresses expression of myeloid differentiation genes and promotes transcriptional repressors and leukemogenic factors.

A–D (A, C) Gene set enrichment analysis of differentially expressed genes (DEGs; significant threshold of 0.05, adjusted *P*-value by False Discovery Rate) commonly upregulated (A) and downregulated (C) in HL60 and Kasumi1 cells upon HIF2α silencing. Indicated are the terms most significantly enriched in the following libraries: gene ontology (GO) biological process, GO molecular function, GO cellular component, Bioplanet, Reactome, and Hallmarks of cancer. Dot sizes represent the number of genes in each term, and colors indicate Enrichment Scores expressed as $-\log_{10}$ (*P*-value). (B, D) Heatmaps of commonly upregulated (B) and downregulated (D) genes within the terms most significantly enriched. The red-blue color scale reflects normalized RPKM (Reads Per Kilobase Million), with red indicating genes with higher expression and blue indicating genes with lower expression. Asterisks indicate genes that are mentioned in the main text. Results for each cell line represent the average of two independent experiments.

E Immunoblot analysis of BCL11A and Runx2 upon HIFα-specific silencing in Kasumi1 cells. Vinculin was used as a loading control. The blot represents one out of three independent experiments with similar results.

F Schematic view of HREs location in the regulatory regions of *BCL11A* and *RUNX2* genes. HREs positions are numbered relative to annotated promoters (green boxes). HRE consensus sequence was obtained from MotifMap (motif ID: M01249).

G ChIP-qPCR for HIF2α with primer pairs amplifying the HREs of *BCL11A* and *RUNX2* and the positive control *SLC7A5* gene in Kasumi1 cells. IgG was used as negative control. Results are represented as percentage of enrichment over input and represent mean ± SD of three biological replicates (Student's *t*-test).

H ChIP-qPCR for HIF2α with primer pairs amplifying the HREs of *BCL11A* and *RUNX2* and the positive control *SLC7A5* gene in shCTRL and shHIF2α Kasumi1 cells. Data were normalized over input and control IgG and presented as fold enrichment over control cells. ChIP-qPCR data represent mean ± SD of three biological replicates (Student's *t*-test).

I Immunoblot analysis of HIF2α upon induction of exogenous HIF2α expression in Kasumi1 cells transduced with the X^{on} system. UI: uninduced cells; I: induced cells. α-tubulin was used as a loading control. The blot represents one out of two independent experiments with similar results.

J Proximity ligation assay with HIF2α and HIF1β antibodies in Kasumi1 cells transduced with the X^{on} system. UI: uninduced cells; I: induced cells. Numbers of nuclear interaction foci/cell are represented ($n = 80$, three independent experiments, Student's *t*-test).

K qPCR analysis of the indicated genes in Kasumi1 cells transduced with the X^{on} system. UI: uninduced cells; I: induced cells. Values are represented as fold change in gene expression compared to uninduced cells. Data represent mean ± SD of three biological replicates (Student's *t*-test).

L ChIP-qPCR for HIF2α with primer pairs amplifying the HREs of *BCL11A* and *RUNX2* and the positive control *SLC7A5* gene in Kasumi1 cells transduced with the X^{on} system. UI: uninduced cells; I: induced cells. Data were normalized over input and control IgG and presented as fold enrichment over control cells. Data represent mean ± SD of three biological replicates (Student's *t*-test).

Source data are available online for this figure.

© 2023 The Authors

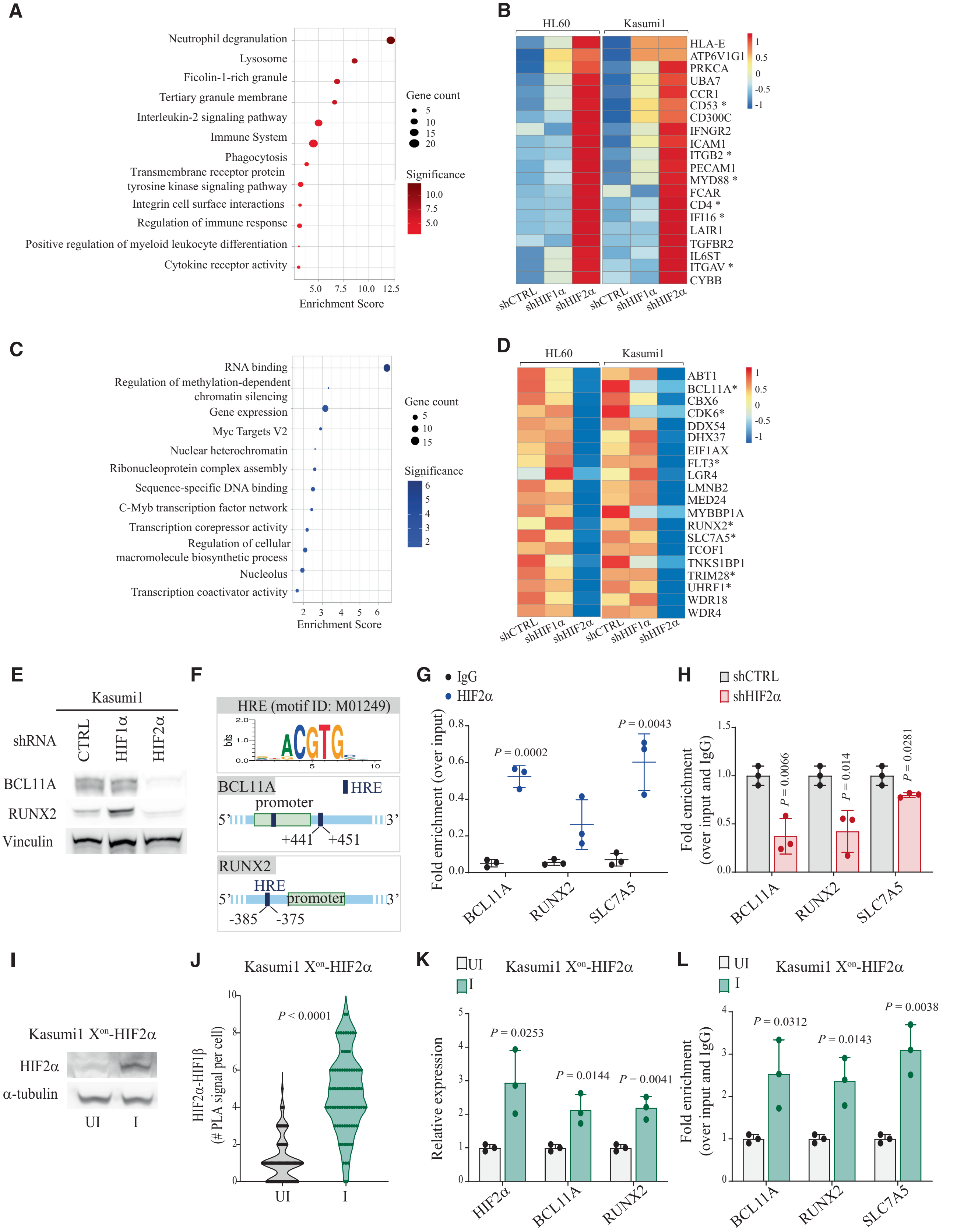
A
Neutrophil degranulation
Lysosome
Ficolin-1-rich granule
Tertiary granule membrane
Interleukin-2 signaling pathway
Immune System
Phagocytosis
Transmembrane receptor protein tyrosine kinase signaling pathway
Integrin cell surface interactions
Regulation of immune response
Positive regulation of myeloid leukocyte differentiation
Cytokine receptor activity
Gene count
Significance
Enrichment Score
B
HL60
Kasumi1
HLA-E
ATP6V1G1
PRKCA
UBA7
CCR1
CD53 *
CD300C
IFNGR2
ICAM1
ITGB2 *
PECAM1
MYD88 *
FCAR
CD4 *
IFI16 *
LAIR1
TGFBR2
IL6ST
ITGAV *
CYBB
shCTRL
shHIF1α
shHIF2α
C
RNA binding
Regulation of methylation-dependent chromatin silencing
Gene expression
Myc Targets V2
Nuclear heterochromatin
Ribonucleoprotein complex assembly
Sequence-specific DNA binding
C-Myb transcription factor network
Transcription corepressor activity
Regulation of cellular macromolecule biosynthetic process
Nucleolus
Transcription coactivator activity
Gene count
Significance
Enrichment Score
D
HL60
Kasumi1
ABT1
BCL11A*
CBX6
CDK6*
DDX54
DHX37
EIF1AX
FLT3*
LGR4
LMNB2
MED24
MYBBP1A
RUNX2*
SLC7A5*
TCOF1
TNKS1BP1
TRIM28*
UHRF1*
WDR18
WDR4
shCTRL
shHIF1α
shHIF2α
E
Kasumi1
shRNA
CTRL
HIF1α
HIF2α
BCL11A
RUNX2
Vinculin
F
HRE (motif ID: M01249)
BCL11A
HRE
promoter
+441
+451
RUNX2
HRE
promoter
-385
-375
G
IgG
HIF2α
Fold enrichment (over input)
P = 0.0002
P = 0.0043
BCL11A
RUNX2
SLC7A5
H
shCTRL
shHIF2α
Fold enrichment (over input and IgG)
P = 0.0066
P = 0.014
P = 0.0281
BCL11A
RUNX2
SLC7A5
I
Kasumi1 X^on-HIF2α
HIF2α
α-tubulin
UI
I
J
Kasumi1 X^on-HIF2α
HIF2α-HIF1β (# PLA signal per cell)
P < 0.0001
UI
I
K
UI
I
Kasumi1 X^on-HIF2α
Relative expression
P = 0.0253
P = 0.0144
P = 0.0041
HIF2α
BCL11A
RUNX2
L
UI
I
Kasumi1 X^on-HIF2α
Fold enrichment (over input and IgG)
P = 0.0312
P = 0.0143
P = 0.0038
BCL11A
RUNX2
SLC7A5

Figure 2.

peaks) and caused decreased H3K27me3 protein levels, as previously observed in macrophages (Li *et al*, 2021; Appendix Fig S3A–D). Genes with reduced H3K27me3 peaks and increased expression upon HIF2α silencing were enriched in functions related to myeloid differentiation (Appendix Fig S3E, Fig EV2D), while genes with gain in H3K27me3 peaks and reduced expression upon HIF2α silencing were enriched in transcriptional/chromatin regulation (Appendix Fig S3F, Fig EV2E). Thus, HIF2α silencing provokes a general deregulation of facultative heterochromatin that is linked to transcriptional regulation. Mechanistically, because genes belonging to the polycomb repressive complex 2 and histone demethylases were not in the HIF2α-regulated transcriptome, we hypothesize that the role of HIF2α in promoting H3K27me3 modifications in AML may be indirect.

Interestingly, analysis of chromatin accessibility revealed that HIF2α-regulated myeloid differentiation genes were not within the gene sets with increased chromatin accessibility upon HIF2α silencing (Appendix Fig S3G). This contrasts with transcriptional regulators including *BCL11A* and *UHRF1*, which are positively regulated by HIF2α and show coherent changes in chromatin accessibility (Appendix Fig S3H). Of note, the regulatory regions of representative myeloid differentiation genes revealed a state of open chromatin in control AML cells that was not modified by HIF2α silencing, a condition that was confirmed in primary AML cells (Gambacorta *et al*, 2022a) (Fig EV2F). Thus, these observations indicate that transcriptional repression of myeloid differentiation genes in AML cells is not always mediated by chromatin compaction at their regulatory regions.

In searching for HIF2α-regulated genes that may be directly implicated in blocking AML differentiation, we focused on Runx2 and BCL11A, which reportedly interfere with the expression of myeloid differentiation genes in AML via transcriptional repression or recruitment of co-repressor complexes (Kuo *et al*, 2009; Sunami *et al*, 2022). We confirmed that specific HIF2α silencing led to reduced Runx2 and BCL11A protein levels in Kasumi1 cells (Fig 2E). Hypoxia-responsive elements (HREs) were identified in the regulatory regions of both genes, and HIF2α was found associated to these genetic elements similarly to the bonafide HIF2α-target gene *SCL7A5* (Elorza *et al*, 2012) (Fig 2F–H). Also, induction of exogenous HIF2α, which correlated with increased association with the obliged transcriptional partner HIF1β, confirmed increased HIF2α association to their regulatory regions and transcriptional upregulation (Fig 2I–L).

In conclusion, we found that in AML HIF2α regulates pro-leukemogenic factors that suppress myeloid differentiation via transcriptional repression. Concordantly, HIF2α inhibition unleashes expression of myeloid differentiation genes and directs AML cells towards a neutrophilic differentiation path.

HIF2α is required for leukemia progression in *in vivo* AML models

To validate and compare the functional consequences of HIF1α and HIF2α suppression *in vivo*, we utilized two AML patient-derived xenograft (PDX) models from leukemia samples collected at diagnosis (Toffalori *et al*, 2019). Leukemic cells expanded in immunodeficient mice were recovered from bone marrow, transduced *ex vivo* with lentiviral vectors containing specific shRNAs along with OFP (orange fluorescent protein) and reinoculated in recipient mice in a competitive assay between transduced and untransduced cells. AML-01 and AML-02 are representative of patients with adverse prognosis (i.e., patients carrying *DNMT3A*, *NPM1*, and *FLT3* mutations; Appendix Fig S4A), but show different *in vivo* disease aggressiveness and lentiviral transduction efficiency (30% and 98%, respectively; Appendix Fig S4B). Overall, we observed that while HIF1α had minor effects on leukemia progression, reduction of HIF2α affected leukemia expansion in both PDX models, albeit in different compartments (Fig 3A). Importantly, analysis of OFP^+ cells revealed that cells with HIF2α suppression were at a competitive disadvantage compared to control-transduced cells, albeit for AML-02 the decrease in OFP^+ cells was not significant (Fig 3B). Surprisingly, analysis of myeloid differentiation at experimental endpoint did not reveal increased $CD11b^+$ cells upon HIF2α silencing (Appendix Fig S4C). However, we observed that OFP^+ cells from the bone marrow of leukemic mice had recovered HIFα expression when compared to gene silencing at preinoculation (Fig 3C), suggesting *in vivo* compensatory mechanisms of HIFα expression that are presently uncharacterized.

Relevance of HIF2α to AML pathogenesis was confirmed via HIF2α downregulation in AML cell lines. We selected Kasumi1 and Molm13 cells as representative of favorable and high-risk AML, respectively. Kasumi1 cells were transplanted subcutaneously (Li *et al*, 2017; Neldeborg *et al*, 2023), while Molm13 were injected intravenously (Migliavacca *et al*, 2016). HIF2α downregulation in Kasumi1 cells drastically reduced tumor progression (Fig 3D and E) and caused a modest and not-significant increase in CD11b expressing cells (Appendix Fig S4D). However, qPCR analysis revealed upregulation of representative myeloid differentiation genes (Fig 3F), indicating that a differentiation process was being triggered. Also, in line with *in vitro* data (Fig EV1C), HIF2α silencing was counter selected *in vivo* (Fig 3G). HIF2α downregulation also affected Molm13 expansion *in vivo*, particularly in spleen and peripheral blood (Fig 3H), which was accompanied by increased CD11b expressing cells in all compartments (Fig 3I) and upregulation of representative myeloid differentiation genes in bone marrow (Fig 3J). In addition, HIF2α silencing was counter selected also in this *in vivo* model (Fig 3K).

Taken together, these data indicate that HIF2α plays a prominent role in AML progression, and its inhibition exerts a significant anti-leukemic function.

Pharmacological inhibition of HIF2α induces AML differentiation

A specific small molecule inhibitor of HIF2α has been recently approved for von-Hippel Lindau disease and is being tested for renal cancer and glioblastoma (Wallace *et al*, 2016; Courtney *et al*, 2018; Renfrow *et al*, 2018; Hasanov & Jonasch, 2021). In all AML cell lines, 2-days treatment with increasing concentrations of PT2385 induced a dose-dependent reduction in cell counts (Figs 4A and EV3A) and a concordant increase in $CD11b^+$ cells (Figs 4B and EV3B) in the absence of cell death (Fig EV3C). Also, PT2385 treatment reduced the expression of genes regulated by HIF2α in AML cells (*FLT3*, *CDK6*, *BCL11A*, *RUNX2*, *UHRF1*, and *TRIM28*), thus confirming a block of HIF2α activity (Figs 4C and EV3D). Notably, because the induction of differentiation at 2 days was modest, we performed a longer time course of PT2385 treatment using the dose of 50 μM to avoid the toxic

 © 2023 The Authors

effects of higher PT2385 doses at 6 days (Fig EV3C). Increased surface expression of CD11b was observed in all cell lines (Figs 4D and EV3E) indicating further commitment to a differentiation program with time. In addition, PT2385 promoted CD11b expression and suppressed HIF2α-regulated genes *ex vivo* in PDX-derived AML cells (Fig EV3F and G).

We next tested the efficacy of PT2385 in an *in vivo* PDX model. PT2385 treatment reduced AML-01 burden in bone marrow, spleen and peripheral blood (Fig 4E–G) and caused increased expression of CD11b in all compartments (Fig 4H–J). Importantly, this was accompanied by increased surface expression of two additional surface markers of myeloid differentiation, CD15 and CD14 (Fig 4H–J)

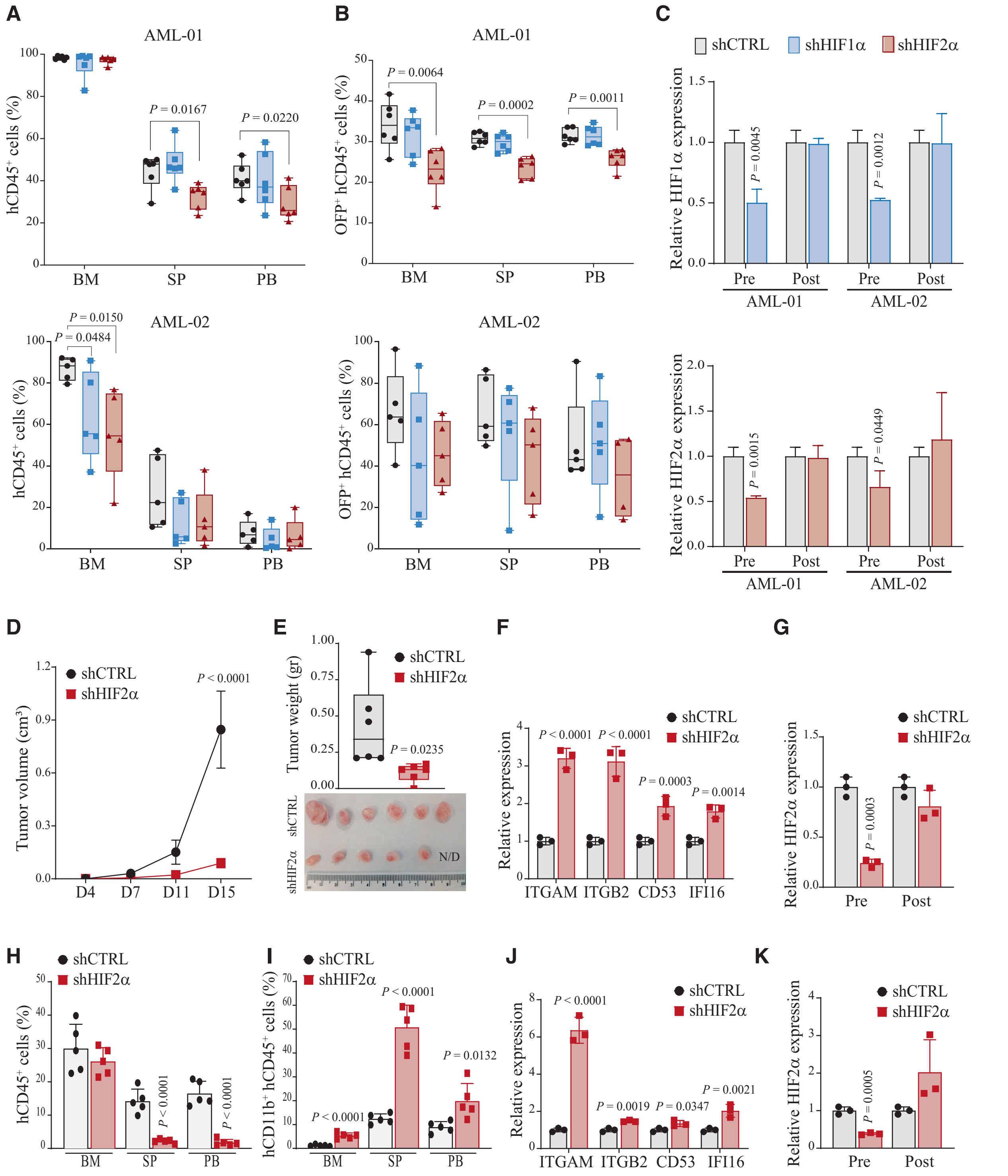

Figure 3.

Figure 3. HIF2α knockdown impairs leukemia progression and induces AML differentiation *in vivo*.

A Percentages of leukemic cells ($hCD45^+$) in the bone marrow (BM), spleen (SP), and peripheral blood (PB) of mice injected with cells derived from AML-01 (n = 6, upper panel) and AML-02 (n = 5, lower panel) PDX and carrying shCTRL, shHIF1α, or shHIF2α. Data are represented in box and whisker plots where the central band denotes the median value, box contains interquartile ranges, while whiskers mark minimum and maximum values. All biological replicates are shown (n = 5/6, Student's *t*-test).

B Percentages of leukemic cells expressing the OFP marker (OFP^+hCD45^+) in mice described in (A). Data are represented in box and whisker plots where the central band denotes the median value, box contains interquartile ranges, while whiskers mark minimum and maximum values. All biological replicates are shown (n = 5/6, Student's *t*-test).

C qPCR of HIF1α (upper panel) and HIF2α (lower panel) genes in pre-inoculated (Pre) AML-01 and AML-02 cells transduced with shCTRL, shHIF1α, and shHIF2α and in cells isolated from the bone marrow of transplanted mice at experimental endpoint (Post). Values indicate fold changes in gene expression compared to shCTRL cells. Data represent mean ± SD of three biological replicates (Student's *t*-test).

D Tumor progression of Kasumi1 cells expressing shCTRL or shHIF2α injected subcutaneously. Tumor volumes were measured at indicated days (D) upon injection. Data represent mean ± SD of six biological replicates (Student's *t*-test).

E Tumor weights of mice described in (D) at experimental endpoint (D20). Data are represented in box and whisker plots where the central band denotes the median value, box contains interquartile ranges, while whiskers mark minimum and maximum values. All biological replicates are shown (n = 6, Student's *t*-test). Lower panel, photograph of excised tumors. N/D: not detected.

F qPCR analysis of the indicated representative myeloid differentiation genes in Kasumi1 shCTRL or shHIF2α tumors. Values are represented as fold change in gene expression compared to shCTRL. Data represent mean ± SD of three biological replicates (Student's *t*-test).

G qPCR of HIF2α in pre-inoculated (Pre) Kasumi1 shCTRL or shHIF2α cells and in cells isolated from Kasumi1 shCTRL or shHIF2α tumors (Post). Values indicate fold changes in gene expression compared to shCTRL cells. Data represent mean ± SD of three biological replicates (Student's *t*-test).

H Percentages of leukemic cells ($hCD45^+$) in the bone marrow (BM), spleen (SP), and peripheral blood (PB) of mice injected intravenously with Molm13 cells with shCTRL or shHIF2α and sacrificed at day 20 post-injection. Data represent mean ± SD of five biological replicates (Student's *t*-test).

I Percentages of Molm13 leukemic cells expressing CD11b ($hCD11b^+hCD45^+$) in mice described in (H). Data represent mean ± SD of five biological replicates (Student's *t*-test).

J qPCR analysis of the indicated representative myeloid differentiation genes in leukemic Molm13 shCTRL or shHIF2α cells recovered from bone marrow. Values are represented as fold change in gene expression compared to shCTRL cells. Data represent mean ± SD of three biological replicates (Student's *t*-test).

K qPCR of HIF2α in pre-inoculated (Pre) Molm13 shCTRL or shHIF2α cells and in cells isolated from bone marrow at experimental endpoint (Post). Values indicate fold changes in gene expression compared to shCTRL cells. Data represent mean ± SD of three biological replicates (Student's *t*-test).

Source data are available online for this figure.

in the absence of sizable cell death (Fig EV4A). Also, repression of representative HIF2α-regulated genes was confirmed (Fig EV4B).

Finally, an additional compound with reported HIF-inhibitory functions was tested *in vivo*. EZN-2208 is a polyethylene glycol conjugate of camptothecin (a topoisomerase inhibitor) that also inhibits HIFα factors (Rapisarda *et al*, 2004; Pastorino *et al*, 2010). Treatment of AML-01 and AML-02 engrafted mice with an established regimen of EZN-2208 that did not induce leukemia cell death (Fig EV4C) affected leukemia progression and induced AML differentiation (Fig EV4D–F). Moreover, EZN-2208 inhibited HIF2α and not HIF1α, as measured by expression of HIFα factors and their regulated genes in AML cells recovered from treated mice (Fig EV4G). Therefore, although we cannot exclude that EZN-2208 exerts additional effects on other molecular targets, with these experiments we identified an additional anti-leukemic compound that triggers AML differentiation.

Taken together, our data demonstrate that HIF2α inhibitory molecule PT2385 recapitulates the induction of AML differentiation observed upon HIF2α knockdown and holds the potential of acting as a novel differentiation agent for AML treatment.

HIF2α is a direct target of ATRA receptors and its inhibition cooperates with ATRA towards AML differentiation

Ongoing efforts to enhance ATRA-induced differentiation and/or proliferation arrest in AML are aiming to combine ATRA with a

Figure 4. Specific inhibition of HIF2α by PT2385 promotes AML differentiation and impairs leukemia progression.

A Cell numbers of HL60 and Kasumi1 cells 2 days after treatment with PT2385 at the indicated doses and compared to vehicle treated cells. Data represent mean ± SD of three biological replicates (one-way ANOVA followed by Tukey's multiple comparison test).

B Percentages of $CD11b^+$ HL60 and Kasumi1 cells 2 days after treatment with PT2385 at the indicated doses and compared to vehicle treated cells. Data represent mean ± SD of three biological replicates (one-way ANOVA followed by Tukey's multiple comparison test).

C qPCR analysis of the indicated genes in HL60 and Kasumi1 cells 2 days after treatment with 200 μM PT2385. Values are represented as fold change in gene expression compared to vehicle treated cells. Data represent mean ± SD of three biological replicates (Student's *t*-test).

D Percentages of $CD11b^+$ HL60 and Kasumi1 cells at the indicated days (D2, D4, D6) after treatment with 50 μM PT2385 and compared to vehicle treated cells. Data represent mean ± SD of three biological replicates (Student's *t*-test).

E Percentage of leukemic cells ($hCD45^+$) in the bone marrow (BM) of mice injected with AML-01 cells and treated with 100 mg/kg PT2385 or vehicle. Data represent mean ± SD of four biological replicates (Student's *t*-test).

F Spleen weights (left graph) and percentages of leukemic cells ($hCD45^+$, right graph) in the spleen (SP) of mice injected with AML-01 cells and treated with 100 mg/kg PT2385 or vehicle. Data represent mean ± SD of four biological replicates (Student's *t*-test).

G Percentages of leukemic cells ($hCD45^+$) in the peripheral blood (PB) of mice injected with AML-01 cells and treated with 100 mg/kg PT2385 or vehicle. Data represent mean ± SD of four biological replicates (Student's *t*-test).

H–J Percentages of leukemic ($hCD45^+$) cells expressing CD11b, CD15 and CD14 in the bone marrow (BM; H), spleen (SP; I) and peripheral blood (PB; J) of mice injected with AML-01 cells and treated with 100 mg/kg PT2385 or vehicle. Data represent mean ± SD of four biological replicates (Student's *t*-test).

Source data are available online for this figure.

© 2023 The Authors

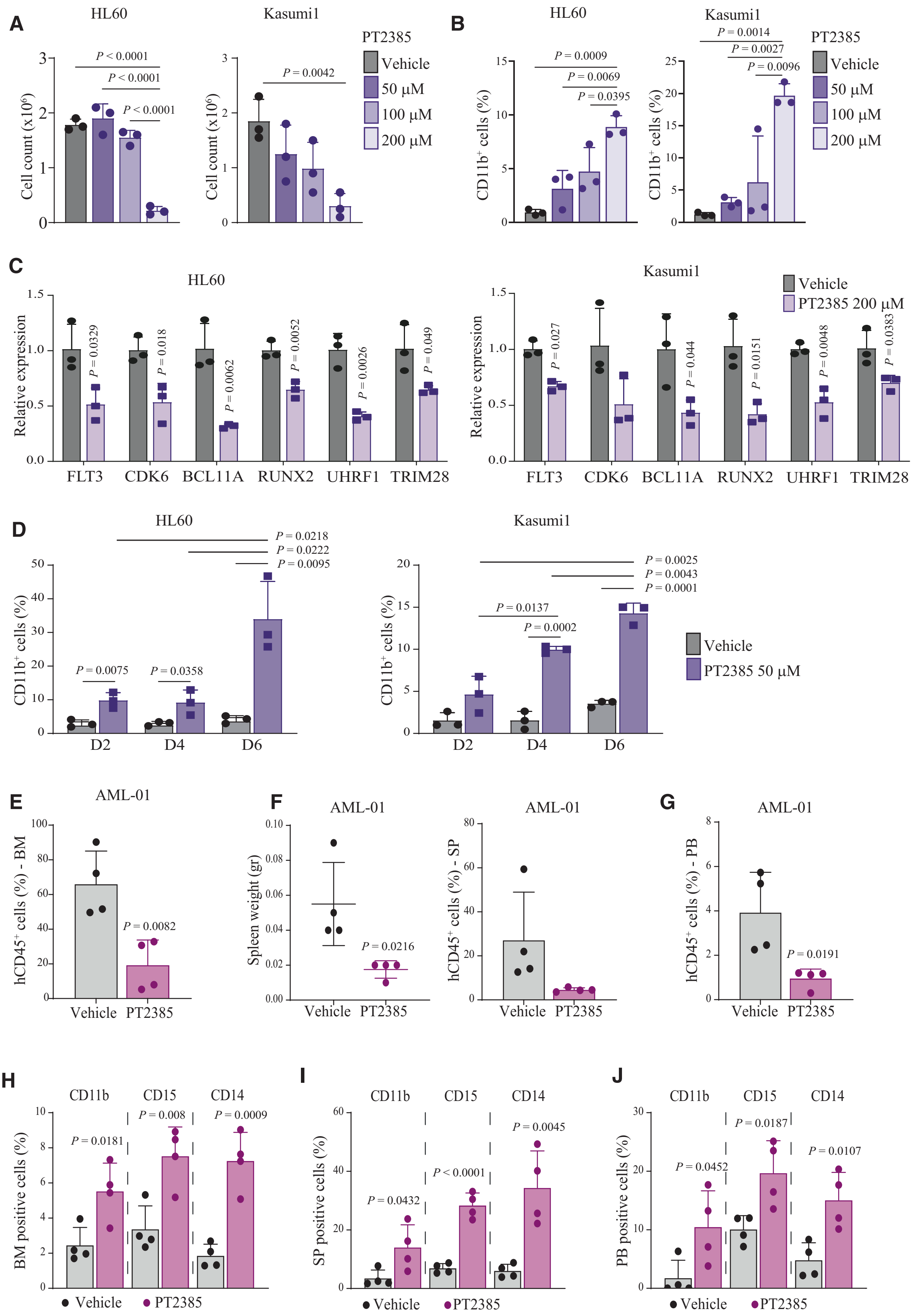
A
HL60
Kasumi1
Cell count (x10⁶)
P < 0.0001
P < 0.0001
P < 0.0001
P = 0.0042
PT2385
Vehicle
50 μM
100 μM
200 μM
B
HL60
Kasumi1
CD11b⁺ cells (%)
P = 0.0009
P = 0.0069
P = 0.0395
P = 0.0014
P = 0.0027
P = 0.0096
PT2385
Vehicle
50 μM
100 μM
200 μM
C
HL60
Kasumi1
Relative expression
P = 0.0329
P = 0.018
P = 0.0062
P = 0.0052
P = 0.0026
P = 0.049
P = 0.027
P = 0.044
P = 0.0151
P = 0.0048
P = 0.0383
FLT3
CDK6
BCL11A
RUNX2
UHRF1
TRIM28
Vehicle
PT2385 200 μM
D
HL60
Kasumi1
CD11b⁺ cells (%)
P = 0.0218
P = 0.0222
P = 0.0095
P = 0.0075
P = 0.0358
P = 0.0025
P = 0.0043
P = 0.0001
P = 0.0137
P = 0.0002
D2
D4
D6
Vehicle
PT2385 50 μM
E
AML-01
hCD45⁺ cells (%) - BM
P = 0.0082
Vehicle
PT2385
F
AML-01
Spleen weight (gr)
P = 0.0216
Vehicle
PT2385
AML-01
hCD45⁺ cells (%) - SP
Vehicle
PT2385
G
AML-01
hCD45⁺ cells (%) - PB
P = 0.0191
Vehicle
PT2385
H
CD11b
CD15
CD14
BM positive cells (%)
P = 0.0181
P = 0.008
P = 0.0009
Vehicle
PT2385
I
CD11b
CD15
CD14
SP positive cells (%)
P = 0.0432
P < 0.0001
P = 0.0045
Vehicle
PT2385
J
CD11b
CD15
CD14
PB positive cells (%)
P = 0.0452
P = 0.0187
P = 0.0107
Vehicle
PT2385

Figure 4.

broad repertoire of anti-leukemia drugs (Geoffroy *et al*, 2021). Based on the newly identified function of HIF2α, we speculated that HIF2α inhibition might cooperate with ATRA to promote AML differentiation. Accordingly, silencing of HIF2α significantly augmented ATRA-induced differentiation in all AML cell lines tested (Figs 5A and EV5A). This was confirmed by use of a low dose of PT2385, which in combination with ATRA provoked a dramatic increase in $CD11b^+$ cells and caused cell cycle arrest, as measured by accumulation of cells in G0/G1 and decreased S phase (Figs 5B and C, and EV5B and C). RNA sequencing upon HIF2α silencing and ATRA administration in Kasumi1 cells revealed upregulation of similar gene families, with an enrichment of myeloid maturation terms within the most significant gene ontologies (Fig 5D). Notably, combined ATRA and HIF2α silencing increased the number of genes within these families and the expression levels of concordantly regulated genes (Fig 5D and E), indicating that HIF2α inhibition and ATRA converge to stimulate the same pro-differentiation programs.

Intriguingly, we observed that ATRA treatment caused a significant upregulation of HIF2α and not HIF1α in AML cell lines (Fig 6A and B). In investigating the molecular mechanism of this regulation, we found that both RARα and RARγ bind the HIF2α promoter. In accordance with previous literature (Rochette-Egly & Germain, 2009), RARs binding occurred in the absence of ATRA stimulation (Fig 6C) and was further increased upon ATRA treatment in a time-dependent manner (Fig 6D). These data show that HIF2α is a direct target of RAR transcription factors and is increasingly expressed upon ATRA administration. These results are in line with the reported induction of hypoxia/stress response genes upon ATRA treatment in a mouse model of AML1-ETO-driven AML (Chee *et al*, 2013).

Because it has been suggested that in AML ATRA may induce differentiation while also promoting self-renewal of leukemic blasts (Geoffroy *et al*, 2021), we wondered whether HIF2α induction may provide a self-renewal signal downstream ATRA, which is hampered by HIF2α inhibition. Along these lines, by overlapping genes that were induced by ATRA and reduced upon HIF2α silencing in Kasumi1 cells (Fig 6E), we found several genes implicated in self-renewal mechanisms in HSCs, such as *PML*, *GFI1*, *KDM6B* and *EPAS1* itself (Ito *et al*, 2008; Rouault-Pierre *et al*, 2013; Mallaney *et al*, 2019; Möröy & Khandanpour, 2019). Increased expression of these genes in Kasumi1 cells treated with ATRA was abolished by concomitant HIF2α inhibition (Fig 6F).

Taken together, our data suggest that targeting HIF2α cooperates with ATRA for differentiation induction and removes a negative feedback loop of HIF2α upregulation that may be implicated in promoting AML self-renewal, thus potentiating ATRA-based therapies in AML.

Discussion

In this work, we place the transcription factor HIF2α within the molecular circuitry of the AML differentiation block and propose that HIF2α inhibition may add therapeutic efficacy to differentiation therapy for AML treatment thus broadening the therapeutic horizon of ATRA beyond APL.

Recent work has implicated HIF transcription factors as either tumor promoters or tumor suppressors in AML, via several studies performed with different experimental approaches in distinct AML subsets (Wang *et al*, 2011; Matsunaga *et al*, 2012; Rouault-Pierre *et al*, 2013; Coltella *et al*, 2014; Velasco-Hernandez *et al*, 2014, 2019; Forristal *et al*, 2015; Gao *et al*, 2015; Vukovic *et al*, 2015; Migliavacca *et al*, 2016). To reconcile this apparent contradiction, we speculated that HIFα factors may exert different functions in specific AML subsets, or at different stages of leukemia development (Magliulo & Bernardi, 2018). Here, we found that both HIF1α and HIF2α exert leukemia-promoting functions in models of established AML (i.e., cell lines and PDX). Importantly, we described a new and specific involvement of HIF2α in blocking AML differentiation. Mechanistically, we observed that HIF2α promotes expression of transcriptional repressors/corepressors (i.e., *RUNX2* and *BCL11A*) that suppress myeloid differentiation genes in AML (Kuo *et al*, 2009; Sunami *et al*, 2022), suggesting that differentiation blockade by HIF2α occurs via regulation of transcriptional repressive programs.

Of note, our *in vitro* experiments were performed in normoxia, thus indicating that HIF factors play important functions in experimental settings where they are not stabilized by low oxygen conditioning. Similar results were obtained by other investigators in hematopoietic progenitors, where HIF2α silencing impacted proliferation and colony formation in normoxic conditions (Rouault-Pierre *et al*, 2013). Notably, by comparing the functions of HIF1α and HIF2α, these investigators identified HIF2α as the main regulator of self-renewal in human long-term repopulating hematopoietic progenitors (Rouault-Pierre *et al*, 2013), an observation that is consistent with HIF2α being expressed specifically in hematopoietic stem and progenitor cells. Therefore, we speculate that the function of HIF2α in the AML differentiation arrest may reflect its physiological function in normal hematopoiesis. In this respect, it is notable that HIF2α is not mutated or overexpressed in AML, thus further

Figure 5. HIF2α inhibition cooperates with ATRA to promote AML differentiation.

A Percentages of $CD11b^+$ HL60 and Kasumi1 cells with shRNAs against HIF1α, HIF2α, or a scrambled shRNA as control (shCTRL) treated with 1 μM ATRA for 2 days. Data represent mean ± SD of three biological replicates (one-way ANOVA followed by Tukey's multiple comparison test).

B Percentages of $CD11b^+$ HL60 and Kasumi1 cells following treatment with 50 μM PT2385, 1 μM ATRA, or combination for 4 days. Data represent mean ± SD of three biological replicates (one-way ANOVA followed by Tukey's multiple comparison test).

C Percentages of HL60 (left graph) and Kasumi1 (right graph) cells in the indicated phases of the cell cycle following treatment with 50 μM PT2385, 1 μM ATRA, or combination for 4 days. Data represent mean ± SD of three biological replicates (Student's *t*-test).

D List of top common upregulated Gene Ontology (GO) terms in Kasumi1 cells upon shHIF2α, 1 μM ATRA treatment or combination with respect to shCTRL cells. Dot sizes represent the number of genes in each term, and colors indicate experimental conditions shown in legend.

E Venn diagram indicating the overlap of commonly upregulated genes (38 genes), which are represented in terms shown in (D). Violin plot indicating fold induction of each of the 38 genes commonly upregulated in each condition. Values represent the Log_2 (FoldChange) with respect to shCTRL cells. Data indicate fold enrichment over control cells (Student's *t*-test).

Source data are available online for this figure.

© 2023 The Authors

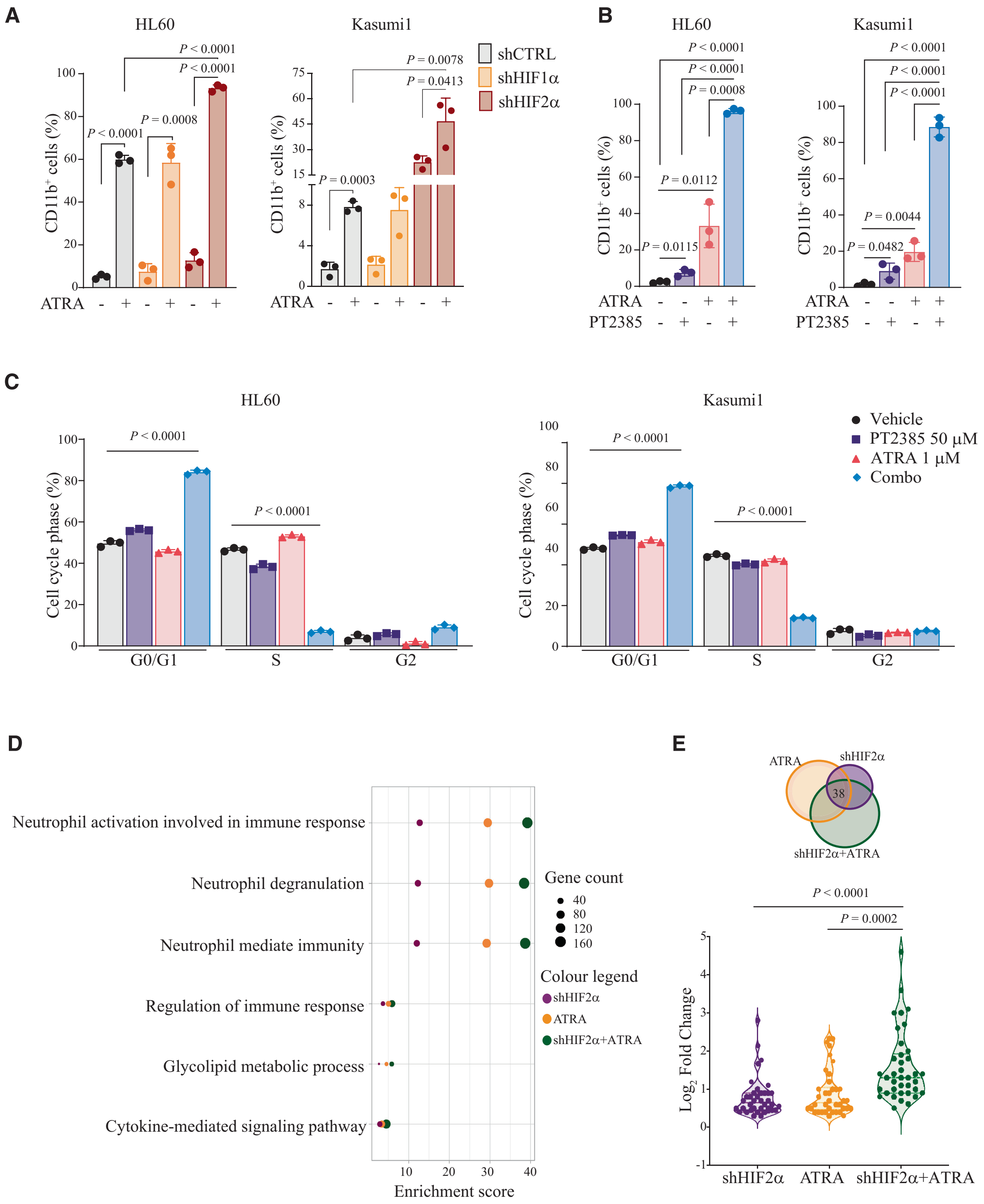

Figure 5.

suggesting that an intrinsic function exerted at specific hematopoietic stages may be exploited in leukemic cells.

Our data carry significant therapeutic implications, as we observed that pharmacological strategies targeting HIF2α impair AML expansion and prompt myeloid differentiation both *in vitro* and *in vivo*. Although HIF2α inhibition is not sufficient *per se* to trigger a complete differentiation program, our studies suggest that HIF2α targeting may cooperate with ATRA for AML treatment. ATRA-based therapies have generated extraordinary results for the treatment of APL but produced disappointing outcomes in other

© 2023 The Authors

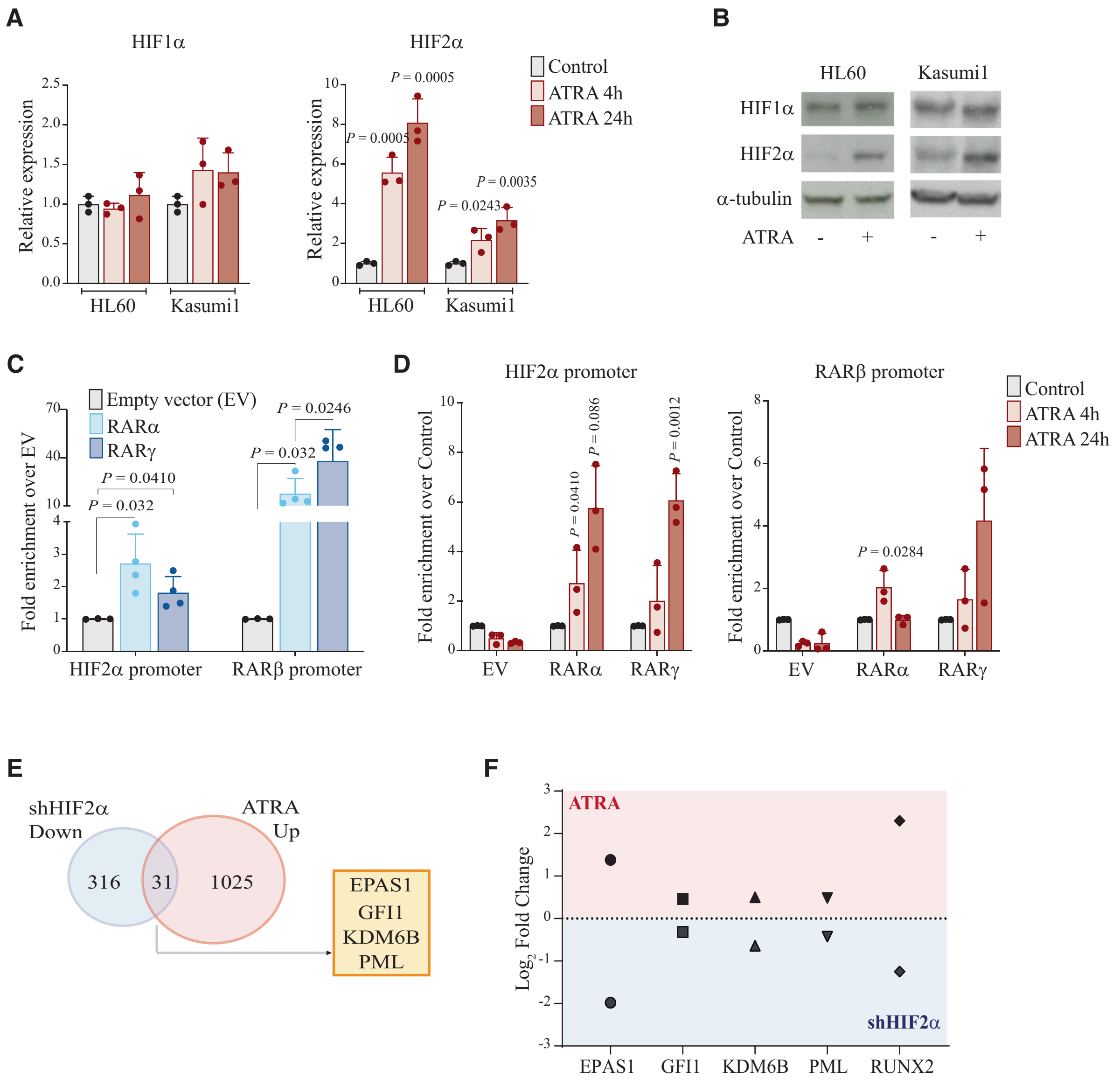

Figure 6. HIF2α is a RAR-target gene upregulated upon ATRA treatment.

A qPCR analysis of HIF1α (left graph) and HIF2α (right graph) in HL60 and Kasumi1 cells after 1 μM ATRA treatment at the indicated time points. Values indicate fold change in gene expression compared to control vehicle treated cells and represent mean ± SD of three biological replicates (Student's *t*-test).

B Immunoblot analysis of HIF1α and HIF2α in HL60 and Kasumi1 cells treated with 1 μM ATRA. α-tubulin was used as a loading control. Blots are indicative of one out of three independent experiments with similar results.

C RARα and RARγ binding to the HIF2α promoter (−1,199 bps from TSS) in Kasumi1 cells expressing FLAG-tagged RARα, RARγ, or empty vector (EV) as negative control. RARβ promoter was used as a positive control (de Thé *et al*, 1990). Values represent fold enrichment of RARs binding compared to EV. Data represent mean ± SD of four biological replicates (Student's *t*-test).

D Fold enrichment of RARα and RARγ binding to the HIF2α promoter (left graph) of the RARβ promoter as a positive control (right graph) following 1 μM ATRA treatment of Kasumi1 cells described in (C). Values represent fold enrichment of RARs binding compared to control vehicle treated cells. Data represent mean ± SD of three biological replicates (Student's *t*-test).

E Venn diagram indicating the overlap of genes upregulated upon ATRA treatment and downregulated upon HIF2α silencing in Kasumi1 cells (31 genes). Genes mentioned in the main text are reported in the box.

F Violin plot showing fold induction of *EPAS1*, *GFI1*, *PML*, and *KDM6B* genes in Kasumi1 cells treated with ATRA alone or in combination with HIF2α silencing. Values represent the Log_2 (FoldChange) with respect to control cells.

Source data are available online for this figure.

AML subsets. In this respect, several limitations are emerging to the use of ATRA for AML treatment: i) few non-APL AML subtypes undergo differentiation upon ATRA treatment alone (El Hajj *et al*, 2015; Ma *et al*, 2016; Verhagen *et al*, 2016; Mugoni *et al*, 2019) and current therapeutic approaches are aiming to ameliorate ATRA efficacy via combination with different types of anti-leukemia drugs

 © 2023 The Authors

(Geoffroy *et al*, 2021); ii) differentiation is not the main contributor to ATRA efficacy in APL, where spontaneous reversal of the differentiated phenotype has been documented (McKenzie *et al*, 2019) and combined treatment with arsenic trioxide (ATO) is necessary to target the driver mutation PML-RARα and eliminate LSCs (Lo-Coco *et al*, 2013; Geoffroy *et al*, 2021); iii) beside promoting differentiation, ATRA increases self-renewal of stem cells in normal hematopoiesis by regulating RARα or RARγ respectively, and a similar antagonistic function has been observed also in AML1-ETO transformed progenitors (Chee *et al*, 2013). Taken together, these observations suggest that further mechanistic investigations are needed to drive clinical application of ATRA-based therapies for AML treatment.

With our work, we suggest that HIF2α partakes to the mechanisms of ATRA-induced self-renewal, as we found that ATRA directly induces HIF2α expression and HIF2α in turn promotes the expression of ATRA-regulated genes implicated in self-renewal of hematopoietic stem cells (*PML*, *KDM6B*, and *GFI1*). Once again, we hypothesize that this molecular circuit may reflect a functional crosstalk that exists in normal hematopoiesis, where RAR transcription factors and HIF signaling may cooperate to promote self-renewal. In addition, we observed that HIF2α inhibition converges onto ATRA transcriptional outputs by increasing expression of gene sets linked to myeloid differentiation. In conclusion, we propose that HIF2α inhibition may add therapeutic value to ATRA-based therapies via a dual mechanism that favors myeloid differentiation, whilst reducing self-renewal.

Materials and Methods

Cell culture

The human leukemic cell lines HL60 (ATCC, CCL-240), Kasumi1 (ATCC, CRL-2724), NB4 (ATCC, 55546), Molm13 (DSMZ, ACC-554), and THP1 (ATCC, TIB-202) were cultured in RPMI-1640 (Euroclone). For lentiviral production, HEK293T cells (ATCC, CRL-11268) were maintained in Dulbecco's modified Eagle medium (DMEM, Lonza). Ms5 stromal cells (DSMZ, ACC-441) were maintained in MEMaplha GlutaMAX (Gibco). All cells were *Mycoplasma* negative. Growth media were supplemented with 10% heat-inactivated fetal bovine serum (FBS, Lonza), and 1% penicillin/streptomycin (Lonza), and maintained at 37°C in a humidified atmosphere containing 5% CO_2.

Reagents and treatments

ATRA (Sigma, Cat# R2625) was used at concentration of 1 μM for the indicated time points. For Western blot analysis, HIF1α and HIF2α were stabilized by 6 h treatment with 250 nM $CoCl_2$ (Sigma, Cat# 232696) or 24 h of hypoxia exposure (1% O_2). PT2385 was purchased from MedChemExpress (Cat# HY-12867). EZN-2208 was provided by Belrose Pharma Inc. LMI070 (Cat# S6642) was purchased from Selleckchem and utilized at concentration of 50 nM for 6 h to induce HIF2α expression in X^{on}-HIF2α Kasumi1 expressing cells. Human recombinant SCF (Cat# 300-07), IL-3 (Cat# 200-03), IL-6 (Cat# 200-06), G-CSF (Cat# 300-23), GM-CSF (Cat# 300-03), and FLT-3 ligand (Cat# 300-19) cytokines were purchased from PeproTech. Human recombinant TPO (Cat# 130-094-011) was purchased from Milteny.

Lentiviral vectors production

Third generation lentivirus (LV) stocks were prepared, concentrated and titrated as previously described (Dull *et al*, 1998; Follenzi *et al*, 2000). Briefly, self-inactivating (SIN) LV vectors were produced by transient transfection of HEK293T cells with the packaging plasmid pMDLg/pRRE, Rev-expressing pCMV-Rev, the VSV-G envelop-encoding pMD2.VSV-G plasmids, and specific shRNA-carrying vectors. For knockdown experiments on patient-derived xenografted cells, shRNAs targeting HIF1α (5′-CCAGTTATGATTGTGAAGTTA-3′) or HIF2α (5′-CCATGAGGAGATTCGTGAGAA-3′) were cloned in the integrating lentiviral vector coexpressing shRNA and mOrange (OFP, orange fluorescent protein) pCCLsin.PPT.SFFV.E-F.Intron.mO2.Wpre (Amendola *et al*, 2009). For knockdown experiments, shRNAs were obtained from Sigma (MISSION®). pLKO.1-puro non-mammalian control (SHC002; 5′-CAACAAGATGAAGAGCACCAA-3′), pLKO.1-puro with HIF1α shRNAs (TRCN0000003809 and TRCN0000003810; shHIF1α#1 5′-CCAGTTATGATTGTGAAGTTA-3′ and shHIF1α#2 5′-GTGATGAAAGAATTACCGAAT-3′ respectively), and pLKO.1-puro with HIF2α shRNAs (TRCN0000003805 and TRCN0000003807; shHIF2α#1 5′-GCGCAAATGTACCCAATGATA-3′ and shHIF2α#2 5′-CCATGAGGAGATTCGTGAGAA-3′ respectively).

To generate inducible HIF2α-expressing Kasumi1 cells, a stable HIF2α mutant (P405A/P531A, Yan *et al*, 2007) was synthesized by GenScript in the X^{on} LMI070-inducible system (Monteys *et al*, 2021) and subcloned in a bidirectional lentiviral vector to obtain the plasmid hPGK.Xon.HA.HIF2amut.WPRE_mhCMV.dNGFR.SV40PA.

Lentiviral infection

Human AML cell lines were spinoculated in medium containing concentrated viral supernatant, 8 μg/ml polybrene, and Hepes 1 M pH 7.4 pH for 90 min at 1,200 *g* at 30°C. After 24 h, fresh medium was added, and cells were allowed to recover for 48 h before antibiotic selection. Optimized puromycin (Sigma, Cat# P8833) concentrations were: 2 μg/ml for Kasumi1, NB4 and Molm13 cells, 6 μg/ml for HL60 cells, and 7 μg/ml for THP1 cells. Experiments were conducted with bulk populations.

For infection of AML cells obtained from PDX mice, cells were isolated from the bone marrow of terminally sick mice using the Mouse Cell Depletion Kit (Milteny, Cat# 130-104-694), according to manufacturer's instructions. Immunomagnetic beads isolation was performed by using MiniMACS separator and LS Columns (MACS, Milteny Biotec). 5×10^6 cells/ml were incubated for 4–6 h in 6-well plates in pre-activation medium containing: Iscove's modified Dulbecco's medium (IMDM, Euroclone), 20% BIT-9500 serum (Stem Cell Technologies, Cat# 09500), 1% Pen/strep, 1% glutamine (Lonza, Cat# LOBEBP17605E), IL-3 (10 ng/ml), TPO (25 ng/ml), G-CSF (10 ng/ml), IL-6 (20 ng/ml), FLT-3 ligand (50 ng/ml), and SCF (50 ng/ml). Cells were incubated overnight with concentrated virus (MOI = 20), and then transplanted into NSG recipient mice within 24 h from bone marrow isolation.

For infection of Kasumi1 cells with the X^{on}-HIF2α expressing vector, cells were plated in 6-well plates at 2.5×10^5 cells/ml and transduced with concentrated viral supernatant. Transduction efficiency

© 2023 The Authors

was measured 7 days upon infection by flow cytometry (FACS Canto II) with 7AAD and anti-human αNGFR-APC (1:100) (Miltenyl Biotec, Cat# 130-113-418). Transduced cells were selected with human CD271 microbead Kit (Miltenyl Biotec, Cat# 130-099-023) following manufacturer instructions.

AML xenograft model

Animal studies were approved by San Raffaele Institutional Animal Care and Use Committee (IACUC, protocol number 897 and 1193). All animals were purchased from Charles River Laboratories, maintained in pathogen-free animal facility and treated in accordance with European Union guidelines. All animals utilized in this study were male. For experiments with PDX AML cells transduced with shRNAs, 1–2 × 10^6 cells resuspended in physiological saline solution were injected via tail vein into 6–8 weeks old NOD/SCID/IL2Rγ^{null} (NSG) recipient mice. Mice were sacrificed when control animals displayed ≥ 10% AML cells in peripheral blood and signs of disease (56 days for AML-01 and 92 days for AML-02). For PT2385 treatment, 5 × 10^6 AML-01 PDX cells were injected intravenously (i.v.) into 6–8 weeks old NSG recipient mice. Treatment was started when leukemic blasts reached 1% in peripheral blood. PT2385 was formulated with 10% absolute ethanol, 30% PEG400, 60% water containing 0.5% methylcellulose and 0.5% Tween 80 (Wallace *et al*, 2016). PT2385 or vehicle solution were administered by oral gavage at 100 mg/kg twice a day for 15 days. For EZN-2208 treatment, 3–5 × 10^6 AML-01 and AML-02 PDX cells were injected intravenously (i.v.) into 6–8 weeks old NSG recipient mice. EZN-2208 treatment started when leukemic blasts reached 5–10% in peripheral blood. EZN-2208 was administered i.v. at 2 mg/kg/dose every other day for 5 days (q2dx5 schedule). Mice were sacrificed at the end of treatment. For *in vivo* experiments with Kasumi1 shCTRL and shHIF2α transduced cells, 1.5 × 10^6 cells were injected subcutaneously into the flanks of 6–8 weeks old NSG recipient mice. Tumor progression was measured every 3/4 days using the caliper method and the formula V (mm^3) = $(\text{width} \times \text{length})^2 \times \pi/6$. Mice where sacrificed at 20 days from injection. For *in vivo* experiments with Molm13 shCTRL and shHIF2α transduced cells, 5 × 10^6 cells were injected intravenously into 6–8 weeks old NSG recipient mice. Mice were sacrificed at 20 days from injection.

Ex vivo treatment of PDX-derived cells

To perform qPCR analysis of HIFα-regulated genes upon EZN-2208 treatment, 10 × 10^6 AML-01 cells recovered from the bone marrow of control and EZN-2208 treated mice were labeled with PE mouse anti-human CD33 antibody (1:100) (BD Biosciences, Cat# 555450), and then separated by using anti-PE MicroBeads (MACS, Miltenyl Biotec Cat# 130-048-801), according to manufacturer's instructions. Immunomagnetic beads isolation was performed by using MiniMACS separator and MS Columns (MACS, Miltenyl Biotec). To perform qPCR analysis of HIF1α and HIF2α gene expression in HIFα-silenced PDX models, bone marrow cells were recovered from mice transplanted with AML-01 and AML-02 and human leukemic cells were isolated with the Mouse Cell Depletion Kit, according to manufacturer's instructions. OFP$^+$ AML-01 and AML-02 cells were sorted using the BD FACSAria Fusion (Becton Dickinson). For qPCR analysis of myeloid differentiation genes from shHIF2α and shCTRL Kasumi1 and Molm13, 2 × 10^6 leukemic cells were recovered from Kasumi1 tumor masses and bone marrow of Molm13 injected mice. For *ex vivo* treatment with PT2385, Ms5 stromal cells were plated at 1 × 10^5/ml in 12-well plates 24 h before AML seeding (Schuringa & Schepers, 2009). AML-01 cells were collected from bone marrow of leukemic mice and plated at 1 × 10^6/ml on Ms5 feeder layer after red blood cells lysis with ACK buffer (Lonza, Cat# BP10-548E). Cells were maintained in MEM alpha GlutaMAX medium (Thermo Fisher Scientific), supplemented with: 20% FBS, 1% Pen/Strep, 50 μM 2-mercaptoethanol, SCF (50 ng/ml), IL-3 (20 ng/ml), IL-6 (20 ng/ml), GM-CSF (20 ng/ml), G-CSF (20 ng/ml), and FLT-3 ligand (50 ng/ml), according to recent literature (Duy *et al*, 2019). PT2385 was added at the indicated concentration and time points.

Cell proliferation

For HIFα-silenced AML cells, 1 × 10^4 cells were seeded in 24-well plates in technical triplicates, and their growth and viability was evaluated by trypan-blue exclusion assay. Cells were counted every 24 h for 4–5 consecutive days, and cell proliferation ratio was calculated as the mean value of triplicates compared to day 0.

For PT2385 treatment, 3 × 10^5 cells were seeded in 24-well plates in triplicates, and their growth and viability was evaluated after 48 h by trypan-blue exclusion assay.

Methylcellulose colony-forming assay

5 × 10^3 cells were resuspended in human methylcellulose base media and cell resuspension solution (R&D Systems, Cat# HSC002) according to manufacturer's instructions and plated in technical duplicates in 6-wells with water supply in the inter-well chamber to prevent evaporation. After 5–7 days, colonies were counted blindly in 20 fields per condition using standard light microscopy (Zeiss Axiovert 40C, 10× objective).

May-Grunwald Giemsa (MGG) staining

1 × 10^5 cells were resuspended in PBS with 10% fetal bovine serum (FBS) and centrifuged on slides by cytospin at 500 rpm for 5 min. For MGG staining, cells were stained by May-Grunwald and Giemsa dyes. After drying and mounting, cellular morphology was examined with AxioImager M2m microscope, 40× objective (Carl Zeiss). To obtain nucleus/cytoplasm ratio, areas of cytoplasm and nucleus were calculated for 30 cells/condition, using ImageJ software (v1.53e, National Institutes of Health).

Proximity ligation assay (PLA)

4 × 10^5 Kasumi1 cells were seeded on coverslips in 12 well plates in RPMI-1640 medium without supplements for 30 min at 37°C in a humidified atmosphere containing 5% CO_2. Adherent cells were fixed with 4% PFA for 10 min at room temperature and permeabilized with PBS and 1% Triton X-100 for 5 min. PLA was performed using rabbit polyclonal anti-HIF2α antibody (1:5,000) (Novus, Cat# NB100-122), mouse monoclonal anti-Arnt1 (H-10) antibody (1:100) (Santa Cruz Biotechnology, Cat# SC-55526), and Duolink® Proximity Ligation Assay kit (Merck, Cat# DUO92101) according to

© 2023 The Authors

manufacturer's instructions. Images were acquired with the Axio Imager.M2 (Zeiss, 60× objectives).

Cell cycle analysis

1 × 10^4 cells were fixed in 70% cold ethanol and stored at −20°C overnight. After fixation, cells were centrifuged at 3,100 *g* for 2 min and washed once with PBS. After centrifugation, cells where permeabilized with PBS Triton X-100 0.25% for 15 min on ice and then washed once with PBS. DNA was stained with 20 μg/mL PI (Merck, Cat# P4864) and RNA was digested with RNaseI 10 μg/ml (ThermoFisher, Cat# 12091021). DNA content was measured with the BD FACSCanto II (Becton Dickinson) and analysis was performed with FCS Express 7 Research software.

Flow cytometry

To measure cell differentiation, AML cell lines were stained at 4°C for 20 min in the dark with PE mouse anti-human CD11b antibody (1:100) (BD Biosciences, Cat# 557321). PDX-derived cells were recovered from bone marrow and spleen by smashing, resuspended in 1× PBS with 10% FBS, and passed through 70 μm cell strainer. Red blood cells from bone marrow, spleen and peripheral blood were lysed with ACK buffer. 2 × 10^6 cells were incubated with rat anti-mouse CD16/CD32 (BD Biosciences, Cat# 553142) for 10 min at room temperature, and then stained at 4°C for 20 min in the dark with APC mouse anti-human CD45 antibody (1:50) (BD Biosciences, Cat# 555485), and Pacific Blue mouse anti-human CD11b antibody (1:100) (BioLegend, Cat# 301316). Annexin V staining was performed using the FITC Annexin V Apoptosis Detection Kit I (BD Biosciences, Cat# 556547), according to manufacturer's instructions. To measure differentiation upon *in vivo* treatment with PT2385, PDX-derived cells recovered from bone marrow, spleen and peripheral blood were incubated with the following antibodies: PE mouse anti-human CD11b antibody (1:100) (BD Biosciences, Cat# 557321), Pacific Blue anti-human CD14 antibody (1:200) (BioLegend, Cat# 301828), and APC/Cyanine7 anti-human CD15 (SSEA-1) antibody (1:200) (BioLegend, Cat# 323048). Fluorescence was measured using the BD FACSCanto II (Becton Dickinson). Gating and analysis were performed using FCS Express 7 Research software.

Quantitative PCR (qPCR)

Total RNA from AML cell lines was isolated using RNeasy mini Kit (Qiagen). Total RNA from PDX-derived cells was isolated using ReliaPrep RNA Cell Miniprep System (Promega). Equal amounts of RNA were reverse transcribed into cDNA with Advantage RT-for-PCR Kit (Clontech) and analyzed by qPCR using a 7900 Fast-Real Time PCR System (Applied Biosystem). Probes for TaqMan assays were purchased from Applied Biosystem (sequences are provided in Appendix Table S3). Each sample was evaluated in technical triplicates, and data were normalized to 18s gene. Relative expression was calculated using the comparative threshold cycle method ($2^{-\Delta\Delta Ct}$), except for assessing basal gene expression where the $2^{-\Delta Ct}$ was used.

Immunoblotting

Proteins were extracted in 0.125 M Tris–HCl pH 6.8 and 2.5% SDS (Sigma), and boiled for 3 min at 95°C. Lysates were sonicated for 1 min (1" ON/1" OFF) at 20% amplitude, and then centrifuged at 15,870 *g* for 10 min at room temperature. Proteins were quantified using BCA protein assay (ThermoFisher Scientific, Cat# 23225). 50–80 μg of total proteins was resolved by standard SDS-PAGE and transferred to a PVDF membrane (Biorad) with transBlot TurboTM Transfer System (Biorad). Blocking of nonspecific sites was performed with 5% milk in PBS 0.1% Tween-20 (PBST), and membranes were incubated overnight with the following antibodies: mouse monoclonal anti-HIF1α (1:250) (BD Biosciences, Cat# 610958), rabbit monoclonal anti-HIF2α (1:250) (Cell Signaling, Cat# 7096S), rabbit monoclonal anti-BCL11A (1:10,000) (Abcam, Cat# ab191401), rabbit monoclonal anti-RUNX2 (1:1,000) (Cell Signaling, Cat# 12556), H3K27me3 (1:5,000) (Merck, Cat# 07–449), H3K9me3 (1:20,000) (Abcam, Cat# AB8898), rabbit monoclonal anti-vinculin (1:10,000) (Cell Signaling, Cat# 13901) and rabbit polyclonal anti-α-tubulin (1:20,000) (Abcam, Cat# ab4074), as loading controls. Incubation with secondary antibodies conjugated with horseradish peroxidase (Santa Cruz Biotechnology, mouse anti-rabbit IgG-HRP Cat# sc-2357, and goat anti-mouse IgG-HRP Cat# sc-2005) was performed 1 h at room temperature in 5% milk/PBST (1:5,000), and immunoreactive proteins were detected using ECL Western Blotting Detection Reagents (GE Healthcare). Densitometric analysis was performed using ImageJ software.

Chromatin immunoprecipitation (ChIP) qPCR, ChIP sequencing and data analysis

ChIP experiments were performed as previously described (Cabianca *et al*, 2012). 50–100 μg of chromatin were used for ChIP of HIF2α and FLAG, whereas 10 μg of chromatin were used for H3K27me3 ChIP-seq. For ChIP-qPCR experiments, GoTaq qPCR Master Mix (Promega) was used to amplify DNA fragments. To measure enrichment, qPCR values were normalized over input. ChIP experiments were performed with the following antibodies: mouse monoclonal anti-H3K27me3 (1:10) (Abcam, Cat# ab6002), rabbit polyclonal anti-HIF2α (1:10) (Novus, Cat# NB100-122), normal rabbit IgG (1:10) (Merck, Cat# 12-370), and mouse monoclonal anti-FLAG (1:50) (Sigma, Cat# F1804). Primer sets for DNA fragments amplification are listed in Appendix Table S4.

For ChIP sequencing (ChIP-seq) experiments, libraries were barcoded, pooled and sequenced on an Illumina Nova-Seq 6000 sequencing system. ChIP-seq experiments were performed generating 40 M reads, 100 nucleotide long, in paired end. After sequencing, reads were trimmed using BBDuk from BBTools suite version 37.36 (http://sourceforge.net/projects/bbmap/), then mapped using BWA-MEM version 0.7.12-r1039 on the human genome assembly GRCh38. Uniquely mapped reads were selected with MarkDuplicates from Picard Tools version 1.104 (http://broadinstitute.github.io/picard/). Further filtering was done on reads mapping in regions present in the ENCODE hg38 blacklist (Amemiya *et al*, 2019) and regions flagged as not primary alignment or with mapping quality score less than 15. ChIP read counts were normalized to library size using the reads per genome coverage (RPGC) function in Deeptools v3.5.1 (https://github.com/deeptools/deepTools/releases/tag/3.5.1) (Ramírez *et al*, 2014), and mean among replicates was calculated using wiggletools v1.2 (https://github.com/Ensembl/WiggleTools). Bigwig files for normalized read counts were visualized using Integrative Genomics Viewer (Robinson *et al*, 2011). ChIP-seq peaks

© 2023 The Authors

were called with MACS2 v2.2.7.1 (https://github.com/macs3-project/MACS/releases/tag/v2.2.7.1). Intersects and unique peaks were determined using BEDOPS v2.4.41 (https://github.com/bedops/bedops/releases/tag/v2.4.) and profile plots were computed with Deeptools (https://doi.org/10.1093/nar/gkw257). Gene annotation was performed with GREAT (PMID 20436461) with Basal plus extension association rule settings.

RNA sequencing and data analysis

For RNA sequencing analysis, specific silencing of HIF1α and HIF2α and absence of compensatory upregulation of HIFα subunits was evaluated by qPCR in HL60 and Kasumi1 cells stably expressing shCTRL, shHIF1α or shHIF2α. For RNA sequencing upon combination of HIFα inhibition and ATRA, Kasumi1 cells were treated with 1 μM ATRA for 24 h. RNA sequencing experiments are representative of two independent experiments performed upon different lentiviral infections. Each sample was processed as follows: (i) total RNA was isolated from 1 to 3 × 10^6 cells with QIAGEN RNeasy Plus Micro Kit, according to manufacturer's instructions. (ii) RNA was treated with DNAse I (Sigma, D5307), according to manufacturer's instructions. (iii) RNA quality was evaluated with a 2100 Bioanalyzer (Agilent) to select RNA with a RIN above 9. TruSeq stranded mRNA protocol was used for 5′/3′ library preparation starting from 100 ng of total RNA. Libraries were barcoded, pooled and sequenced on an Illumina Nova-Seq 6000 sequencing system. For each run, RNA sequencing experiments were performed generating 30 M single-end reads, 100 nucleotide long. After trimming, sequences were aligned using the STAR aligner (Dobin *et al*, 2013) to human reference genome GRCh38, and counted with featureCounts (Liao *et al*, 2014) on the last Gencode (Harrow *et al*, 2012) release for RNA sequencing. Differential gene expression was evaluated in R/BioConductor (Huber *et al*, 2015) using the DESeq2 package (Love *et al*, 2014). A significant threshold of 0.05, adjusting the *P*-value by FDR (False Discovery Rate) was established to identify differentially expressed genes. Functional enrichment analysis were performed using Enrichr (Kuleshov *et al*, 2016).

ATAC sequencing and data analysis

ATAC sequencing experiments are representative of three experimental replicates. A total of 6 × 10^5 cells were lysed with digitonin (Promega, Cat# G944A) and tagmented with an engineered Tn5 transposase (Illumina, Cat# 15027865) at 37°C for 30 min, following a protocol optimized for blood cells (Corces *et al*, 2016). Tagmented DNA was purified using the MinElute Reaction Cleanup kit (Qiagen) and then amplified with 10 cycles of PCR. Before sequencing, fragments with a 1–5 kb size range were removed by magnetic separation with AMPure XP beads (Beckman Coulter, Cat# A63881). DNA concentration was measured with the Qubit fluorometer (Life Technologies), and quality of samples' enrichment was assessed using Agilent TapeStation system. Sequencing was performed using Illumina High throughput Sequencing technology (NovaSeq 6000). Raw reads were trimmed using the software BBDuck. Reads were aligned to the human genome assembly (GRCh38) using the BWA software with standard parameters, and uniquely mapped reads were selected with MarkDuplicates from Picard Tools [http://broadinstitute.github.io/picard/]. Further filtering was done on reads mapping in regions present in the ENCODE hg38 blacklist (Amemiya *et al*, 2019). ChIP read counts were normalized to library size using the reads per genome coverage (RPGC) function in Deeptools v3.5.1 and mean among replicates was calculated using wiggletools v1.2. Bigwig files for normalized read counts were visualized using Integrative Genomics Viewer (Robinson *et al*, 2011). Peaks were called with MACS2 v2.2.7.1. Intersects and unique peaks were determined using BEDOPS v2.4.41 (https://github.com/bedops/bedops/releases/tag/v2.4.) and profile plots were computed with Deeptools (https://doi.org/10.1093/nar/gkw257). Gene annotation was performed with GREAT (PMID 20436461) with Two nearest genes association rule settings.

The paper explained

Problem
Acute myeloid leukemia (AML) is an aggressive disease affecting blood cells of the myeloid lineage. AML patients have a 5-year overall survival rate of less than 30%, and new therapeutic strategies are urgently needed to improve this grim prognosis. In AML, uncontrolled cell proliferation is intertwined with differentiation arrest, that is the inability of cells with a progenitor phenotype to undergo differentiation, to mature and self-exhausting myeloid cells. The molecular basis of this differentiation arrest is complex and a matter of ongoing investigation.

Results
We found that the transcription factor HIF2α, a gene that evolved to adapt cellular physiology to variations in oxygen tension, partakes to the AML differentiation block. We identify important transcriptional regulators and suppressors of myeloid differentiation in the HIF2α-regulated transcriptome and demonstrate that inhibiting HIF2α via genetic or pharmacological manipulation prompts AML differentiation, induces leukemia debulking, and potentiates the effect of all-*trans* retinoic acid (ATRA), a compound that has revolutionized the treatment of acute promyelocytic leukemia.

Impact
This study adds new insights into the molecular mechanisms that suppress differentiation programs in AML and proposes a novel therapeutic strategy for leukemia debulking via HIF2α inhibition. Because a small molecule inhibitor of HIF2α has been recently generated and is entering the clinic for solid cancers, this work sets the basis for extending the use of this compound to another disease in need of additional therapies and with the therapeutic endpoint of cell exhaustion via differentiation, rather than the conventional cytotoxic or cytostatic activity of anticancer agents.

Statistical analysis

Animals were randomized into different treatment groups such that leukemia engraftment was similar between the groups. The experiments were conducted as non-blind tests and no mice were excluded from the experiments. One-way ANOVA was used for comparison of three or more groups, with the addition of *post-hoc* Tukey's multiple comparison test. Two-sided Student's *t*-test was used for comparison of two groups. All data are expressed as means ± standard deviations (SD), and significance is indicated with exact *P*-value in the figures. Data were processed using GraphPad Prism version 9.0.2 (GraphPad Software, San Diego,

© 2023 The Authors

California, USA, www.graphpad.com), and the R statistical environment.

Data availability

RNA, ChIP and ATAC sequencing data have been deposited in Gene Expression Omnibus (GEO) at GSE202107 (https://www.ncbi.nlm.nih.gov/geo/query/acc.cgi?acc=GSE202107).

Expanded View for this article is available online.

Acknowledgements
The authors would like to thank all authors for valuable discussion and support; L. Greenberger, Y. Zhang and Belrose Pharma Inc. for providing EZN-2208; the San Raffaele Hospital Flow cytometry Resource, Advanced Cytometry Technical Applications Laboratory (FRACTAL) for help in performing FACS experiments; the San Raffaele Hospital Advanced Light and Electron Microscopy BioImaging Center (ALEMBIC) for imaging studies. This work was supported by Ministero della Salute (Italian Ministry of Health; Ricerca Finalizzata, RF-2019-12369841). Synopsis figure was created with BioRender.com. Open access funding provided by BIBLIOSAN. [Correction added on 16 October 2023, after first online publication: funding statement has been added.]

Author contributions
Daniela Magliulo: Conceptualization; data curation; formal analysis; validation; investigation; visualization; methodology; writing – original draft; project administration; writing – review and editing. **Matilde Simoni:** Data curation; investigation; methodology. **Carolina Caserta:** Investigation; methodology. **Cristina Fracassi:** Data curation; investigation. **Serena Belluschi:** Investigation; methodology. **Kety Giannetti:** Methodology. **Raffaella Pini:** Data curation. **Ettore Zapparoli:** Data curation. **Stefano Beretta:** Data curation. **Martina Uggè:** Investigation; methodology. **Eleonora Draghi:** Resources. **Federico Rossari:** Methodology. **Nadia Coltella:** Resources; methodology. **Cristina Tresoldi:** Resources. **Marco J Morelli:** Conceptualization; resources. **Raffaella Di Micco:** Conceptualization; resources. **Bernhard Gentner:** Conceptualization; resources. **Luca Vago:** Conceptualization; resources. **Rosa Bernardi:** Conceptualization; supervision; funding acquisition; investigation; writing – original draft; project administration; writing – review and editing.

Disclosure and competing interests statement
The authors declare that they have no conflict of interest.

References

Amemiya HM, Kundaje A, Boyle AP (2019) The ENCODE blacklist: identification of problematic regions of the genome. *Sci Rep* 9: 9354

Amendola M, Passerini L, Pucci F, Gentner B, Bacchetta R, Naldini L (2009) Regulated and multiple miRNA and siRNA delivery into primary cells by a lentiviral platform. *Mol Ther* 17: 1039–1052

Bagger FO, Sasivarevic D, Sohi SH, Laursen LG, Pundhir S, Sønderby CK, Winther O, Rapin N, Porse BT (2016) BloodSpot: a database of gene expression profiles and transcriptional programs for healthy and malignant haematopoiesis. *Nucleic Acids Res* 44: D917–D924

Cabianca DS, Casa V, Bodega B, Xynos A, Ginelli E, Tanaka Y, Gabellini D (2012) A long ncRNA links copy number variation to a polycomb/trithorax epigenetic switch in FSHD muscular dystrophy. *Cell* 149: 819–831

Cerami E, Gao J, Dogrusoz U, Gross BE, Sumer SO, Aksoy BA, Jacobsen A, Byrne CJ, Heuer ML, Larsson E *et al* (2012) The cBio cancer genomics portal: an open platform for exploring multidimensional cancer genomics data. *Cancer Discov* 2: 401–404

Chee LCY, Hendy J, Purton LE, McArthur GA (2013) ATRA and the specific RARα agonist, NRX195183, have opposing effects on the clonogenicity of pre-leukemic murine AML1-ETO bone marrow cells. *Leukemia* 27: 1369–1380

Coltella N, Percio S, Valsecchi R, Cuttano R, Guarnerio J, Ponzoni M, Pandolfi PP, Melillo G, Pattini L, Bernardi R (2014) HIF factors cooperate with PML-RARα to promote acute promyelocytic leukemia progression and relapse. *EMBO Mol Med* 6: 640–650

Corces MR, Buenrostro JD, Wu B, Greenside PG, Chan SM, Koenig JL, Snyder MP, Pritchard JK, Kundaje A, Greenleaf WJ *et al* (2016) Lineage-specific and single-cell chromatin accessibility charts human hematopoiesis and leukemia evolution. *Nat Genet* 48: 1193–1203

Courtney KD, Infante JR, Lam ET, Figlin RA, Rini BI, Brugarolas J, Zojwalla NJ, Lowe AM, Wang K, Wallace EM *et al* (2018) Phase I dose-escalation trial of PT2385, a first-in-class hypoxia-inducible factor-2α antagonist in patients with previously treated advanced clear cell renal cell carcinoma. *J Clin Oncol* 36: 867–874

Courtney KD, Ma Y, Diaz de Leon A, Christie A, Xie Z, Woolford L, Singla N, Joyce A, Hill H, Madhuranthakam AJ *et al* (2020) HIF-2 complex dissociation, target inhibition, and acquired resistance with PT2385, a first-in-class HIF-2 inhibitor, in patients with clear cell renal cell carcinoma. *Clin Cancer Res* 26: 793–803

Czerwińska P, Mazurek S, Wiznerowicz M (2017) The complexity of TRIM28 contribution to cancer. *J Biomed Sci* 24: 63

Dengler VL, Galbraith M, Espinosa JM (2014) Transcriptional regulation by hypoxia inducible factors. *Crit Rev Biochem Mol Biol* 49: 1–15

Dobin A, Davis CA, Schlesinger F, Drenkow J, Zaleski C, Jha S, Batut P, Chaisson M, Gingeras TR (2013) STAR: ultrafast universal RNA-seq aligner. *Bioinformatics* 29: 15–21

Döhner H, Estey E, Grimwade D, Amadori S, Appelbaum FR, Büchner T, Dombret H, Ebert BL, Fenaux P, Larson RA *et al* (2017) Diagnosis and management of AML in adults: 2017 ELN recommendations from an international expert panel. *Blood* 129: 424–447

Dull T, Zufferey R, Kelly M, Mandel RJ, Nguyen M, Trono D, Naldini L (1998) A third-generation lentivirus vector with a conditional packaging system. *J Virol* 72: 8463–8471

Duy C, Teater M, Garrett-Bakelman FE, Lee TC, Meydan C, Glass JL, Li M, Hellmuth JC, Mohammad HP, Smitheman KN *et al* (2019) Rational targeting of cooperating layers of the epigenome yields enhanced therapeutic efficacy against AML. *Cancer Discov* 9: 872–889

El Hajj H, Dassouki Z, Berthier C, Raffoux E, Ades L, Legrand O, Hleihel R, Sahin U, Tawil N, Salameh A *et al* (2015) Retinoic acid and arsenic trioxide trigger degradation of mutated NPM1, resulting in apoptosis of AML cells. *Blood* 125: 3447–3454

Elorza A, Soro-Arnáiz I, Meléndez-Rodríguez F, Rodríguez-Vaello V, Marsboom G, de Cárcer G, Acosta-Iborra B, Albacete-Albacete L, Ordóñez A, Serrano-Oviedo L *et al* (2012) HIF2α acts as an mTORC1 activator through the amino acid carrier SLC7A5. *Mol Cell* 48: 681–691

Figueroa ME, Abdel-Wahab O, Lu C, Ward PS, Patel J, Shih A, Li Y, Bhagwat N, Vasanthakumar A, Fernandez HF *et al* (2010) Leukemic IDH1 and IDH2

© 2023 The Authors

mutations result in a hypermethylation phenotype, disrupt TET2 function, and impair hematopoietic differentiation. *Cancer Cell* 18: 553 – 567

Follenzi A, Ailles LE, Bakovic S, Geuna M, Naldini L (2000) Gene transfer by lentiviral vectors is limited by nuclear translocation and rescued by HIV-1 pol sequences. *Nat Genet* 25: 217 – 222

Forristal CE, Brown AL, Helwani FM, Winkler IG, Nowlan B, Barbier V, Powell RJ, Engler GA, Diakiw SM, Zannettino ACW *et al* (2015) Hypoxia inducible factor (HIF)-2α accelerates disease progression in mouse models of leukemia and lymphoma but is not a poor prognosis factor in human AML. *Leukemia* 29: 2075 – 2085

Gambacorta V, Beretta S, Ciccimarra M, Zito L, Giannetti K, Andrisani A, Gnani D, Zanotti L, Oliveira G, Carrabba MG *et al* (2022a) Integrated multiomic profiling identifies the epigenetic regulator PRC2 as a therapeutic target to counteract leukemia immune escape and relapse. *Cancer Discov* 12: 1449 – 1461

Gambacorta V, Beretta S, Cittaro D, Merelli I, Di Micco R, Vago L (2022b) Gene Expression Omnibus GSE197416. (https://www.ncbi.nlm.nih.gov/geo/query/acc.cgi?acc=GSE197416). [DATASET]

Gao XN, Yan F, Lin J, Gao L, Lu XL, Wei SC, Shen N, Pang JX, Ning QY, Komeno Y *et al* (2015) AML1/ETO cooperates with HIF1α to promote leukemogenesis through DNMT3a transactivation. *Leukemia* 29: 1730 – 1740

Geoffroy M-C, Esnault C, de Thé H (2021) Retinoids in hematology: a timely revival? *Blood* 137: 2429 – 2437

Gilliland DG, Griffin JD (2002) The roles of FLT3 in hematopoiesis and leukemia. *Blood* 100: 1532 – 1542

van Gils N, Verhagen HJMP, Smit L (2017) Reprogramming acute myeloid leukemia into sensitivity for retinoic-acid-driven differentiation. *Exp Hematol* 52: 12 – 23

Harrow J, Frankish A, Gonzalez JM, Tapanari E, Diekhans M, Kokocinski F, Aken BL, Barrell D, Zadissa A, Searle S *et al* (2012) GENCODE: The reference human genome annotation for the ENCODE project. *Genome Res* 22: 1760 – 1774

Hasanov E, Jonasch E (2021) MK-6482 as a potential treatment for von Hippel-Lindau disease-associated clear cell renal cell carcinoma. *Expert Opin Investig Drugs* 30: 495 – 504

Huber W, Carey VJ, Gentleman R, Anders S, Carlson M, Carvalho BS, Bravo HC, Davis S, Gatto L, Girke T *et al* (2015) Orchestrating high-throughput genomic analysis with Bioconductor. *Nat Methods* 12: 115 – 121

Ito K, Bernardi R, Morotti A, Matsuoka S, Saglio G, Ikeda Y, Rosenblatt J, Avigan DE, Teruya-Feldstein J, Pandolfi PP (2008) PML targeting eradicates quiescent leukaemia-initiating cells. *Nature* 453: 1072 – 1078

Kim K-Y, Tanaka Y, Su J, Cakir B, Xiang Y, Patterson B, Ding J, Jung Y-W, Kim J-H, Hysolli E *et al* (2018) Uhrf1 regulates active transcriptional marks at bivalent domains in pluripotent stem cells through Setd1a. *Nat Commun* 9: 2583

Kocabas F, Zheng J, Thet S, Copeland NG, Jenkins NA, DeBerardinis RJ, Zhang C, Sadek HA (2012) Meis1 regulates the metabolic phenotype and oxidant defense of hematopoietic stem cells. *Blood* 120: 4963 – 4972

Kuleshov MV, Jones MR, Rouillard AD, Fernandez NF, Duan Q, Wang Z, Koplev S, Jenkins SL, Jagodnik KM, Lachmann A *et al* (2016) Enrichr: a comprehensive gene set enrichment analysis web server 2016 update. *Nucleic Acids Res* 44: W90 – W97

Kuo Y, Zaidi SK, Gornostaeva S, Komori T, Stein GS, Castilla LH (2009) Runx2 induces acute myeloid leukemia in cooperation with Cbfβ-SMMHC in mice. *Blood* 113: 3323 – 3332

Li Y, Ning Q, Shi J, Chen Y, Jiang M, Gao L, Huang W, Jing Y, Huang S, Liu A *et al* (2017) A novel epigenetic AML1-ETO/THAP10/miR-383 mini-circuitry contributes to t(8;21) leukaemogenesis. *EMBO Mol Med* 9: 933 – 949

Li X, Zhang X, Xia J, Zhang L, Chen B, Lian G, Yun C, Yang J, Yan Y, Wang P *et al* (2021) Macrophage HIF-2α suppresses NLRP3 inflammasome activation and alleviates insulin resistance. *Cell Rep* 36: 109607

Liao Y, Smyth GK, Shi W (2014) FeatureCounts: an efficient general purpose program for assigning sequence reads to genomic features. *Bioinformatics* 30: 923 – 930

Lo-Coco F, Avvisati G, Vignetti M, Thiede C, Orlando SM, Iacobelli S, Ferrara F, Fazi P, Cicconi L, Di Bona E *et al* (2013) Retinoic acid and arsenic trioxide for acute promyelocytic leukemia. *N Engl J Med* 369: 111 – 121

Love MI, Huber W, Anders S (2014) Moderated estimation of fold change and dispersion for RNA-seq data with DESeq2. *Genome Biol* 15: 550

Ma HS, Greenblatt SM, Shirley CM, Duffield AS, Bruner JK, Li L, Nguyen B, Jung E, Aplan PD, Ghiaur G *et al* (2016) All-trans retinoic acid synergizes with FLT3 inhibition to eliminate FLT3/ITD+ leukemia stem cells in vitro and in vivo. *Blood* 127: 2867 – 2878

Magliulo D, Bernardi R (2018) HIF-α factors as potential therapeutic targets in leukemia. *Expert Opin Ther Targets* 22: 917 – 928

Mallaney C, Ostrander EL, Celik H, Kramer AC, Martens A, Kothari A, Koh WK, Haussler E, Iwamori N, Gontarz P *et al* (2019) Kdm6b regulates context-dependent hematopoietic stem cell self-renewal and leukemogenesis. *Leukemia* 33: 2506 – 2521

Matsunaga T, Imataki O, Torii E, Kameda T, Shide K, Shimoda H, Kamiunten A, Sekine M, Taniguchi Y, Yamamoto S *et al* (2012) Elevated HIF-1α expression of acute myelogenous leukemia stem cells in the endosteal hypoxic zone may be a cause of minimal residual disease in bone marrow after chemotherapy. *Leuk Res* 36: e122 – e124

McKenzie MD, Ghisi M, Oxley EP, Ngo S, Cimmino L, Esnault C, Liu R, Salmon JM, Bell CC, Ahmed N *et al* (2019) Interconversion between tumorigenic and differentiated states in acute myeloid leukemia. *Cell Stem Cell* 25: 258 – 272

Migliavacca J, Percio S, Valsecchi R, Ferrero E, Spinelli A, Ponzoni M, Tresoldi C, Pattini L, Bernardi R, Coltella N (2016) Hypoxia inducible factor-1α regulates a pro-invasive phenotype in acute monocytic leukemia. *Oncotarget* 7: 53540 – 53557

Momparler RL, Côté S, Momparler LF (2020) Epigenetic modulation of self-renewal capacity of leukemic stem cells and implications for chemotherapy. *Epigenomes* 4: 3

Monteys AM, Hundley AA, Ranum PT, Tecedor L, Muehlmatt A, Lim E, Lukashev D, Sivasankaran R, Davidson BL (2021) Regulated control of gene therapies by drug-induced splicing. *Nature* 596: 291 – 295

Möröy T, Khandanpour C (2019) Role of GFI1 in epigenetic regulation of MDS and AML pathogenesis: mechanisms and therapeutic implications. *Front Oncol* 9: 824

Mugoni V, Panella R, Cheloni G, Chen M, Pozdnyakova O, Stroopinsky D, Guarnerio J, Monteleone E, Lee JD, Mendez L *et al* (2019) Vulnerabilities in mIDH2 AML confer sensitivity to APL-like targeted combination therapy. *Cell Res* 29: 446 – 459

Neldeborg S, Soerensen JF, Møller CT, Bill M, Gao Z, Bak RO, Holm K, Sorensen B, Nyegaard M, Luo Y *et al* (2023) Dual intron-targeted CRISPR-Cas9-mediated disruption of the AML RUNX1-RUNX1T1 fusion gene effectively inhibits proliferation and decreases tumor volume in vitro and in vivo. *Leukemia* 37: 1792 – 1801

Oleksiewicz U, Gładych M, Raman AT, Heyn H, Mereu E, Chlebanowska P, Andrzejewska A, Sozańska B, Samant N, Fąk K *et al* (2017) TRIM28 and interacting KRAB-ZNFs control self-renewal of human pluripotent stem cells through epigenetic repression of pro-differentiation genes. *Stem Cell Reports* 9: 2065 – 2080

 © 2023 The Authors

Pastorino F, Loi M, Sapra P, Becherini P, Cilli M, Emionite L, Ribatti D, Greenberger LM, Horak ID, Ponzoni M (2010) Tumor regression and curability of preclinical neuroblastoma models by PEGylated SN38 (EZN-2208), a novel topoisomerase I inhibitor. *Clin Cancer Res* 16: 4809 – 4821

Ramírez F, Dündar F, Diehl S, Grüning BA, Manke T (2014) deepTools: a flexible platform for exploring deep-sequencing data. *Nucleic Acids Res* 42: W187 – W191

Rapisarda A, Shoemaker RH, Melillo G (2004) Targeting topoisomerase I to inhibit hypoxia inducible factor 1. *Cell Cycle* 3: 172 – 175

Renfrow JJ, Soike MH, Debinski W, Ramkissoon SH, Mott RT, Frenkel MB, Sarkaria JN, Lesser GJ, Strowd RE (2018) Hypoxia-inducible factor 2α: a novel target in gliomas. *Future Med Chem* 10: 2227 – 2236

Robinson JT, Thorvaldsdóttir H, Winckler W, Guttman M, Lander ES, Getz G, Mesirov JP (2011) Integrative genomics viewer. *Nat Biotechnol* 29: 24 – 26

Rochette-Egly C, Germain P (2009) Dynamic and combinatorial control of gene expression by nuclear retinoic acid receptors (RARs). *Nucl Recept Signal* 7: e005

Rouault-Pierre K, Lopez-Onieva L, Foster K, Anjos-Afonso F, Lamrissi-Garcia I, Serrano-Sanchez M, Mitter R, Ivanovic Z, de Verneuil H, Gribben J *et al* (2013) HIF-2a protects human hematopoietic stem/progenitors and acute myeloid leukemic cells from apoptosis induced by endoplasmic reticulum stress. *Cell Stem Cell* 13: 549 – 563

Scheicher R, Hoelbl-Kovacic A, Bellutti F, Tigan A-S, Prchal-Murphy M, Heller G, Schneckenleithner C, Salazar-Roa M, Zöchbauer-Müller S, Zuber J *et al* (2015) CDK6 as a key regulator of hematopoietic and leukemic stem cell activation. *Blood* 125: 90 – 101

Schulz K, Milke L, Rübsamen D, Menrad H, Schmid T, Brüne B (2012) HIF-1α protein is upregulated in HIF-2α depleted cells via enhanced translation. *FEBS Lett* 586: 1652 – 1657

Schuringa JJ, Schepers H (2009) Ex vivo assays to study self-renewal and long-term expansion of genetically modified primary human acute myeloid leukemia stem cells. *Methods Mol Biol* 538: 287 – 300

Sunami Y, Yokoyama T, Yoshino S, Takahara T, Yamazaki Y, Harada H, Nakamura T (2022) BCL11A promotes myeloid leukemogenesis by repressing PU.1 target genes. *Blood Adv* 6: 1827 – 1843

de Thé H, Vivanco-Ruiz MM, Tiollais P, Stunnenberg H, Dejean A (1990) Identification of a retinoic acid responsive element in the retinoic acid receptor beta gene. *Nature* 343: 177 – 180

Toffalori C, Zito L, Gambacorta V, Riba M, Oliveira G, Bucci G, Barcella M, Spinelli O, Greco R, Crucitti L *et al* (2019) Immune signature drives leukemia escape and relapse after hematopoietic cell transplantation. *Nat Med* 25: 603 – 611

Tyner JW, Tognon CE, Bottomly D, Wilmot B, Kurtz SE, Savage SL, Long N, Schultz AR, Traer E, Abel M *et al* (2018) Functional genomic landscape of acute myeloid leukaemia. *Nature* 562: 526 – 531

Velasco-Hernandez T, Hyrenius-Wittsten A, Rehn M, Bryder D, Cammenga J (2014) HIF-1α can act as a tumor suppressor gene in murine acute myeloid leukemia. *Blood* 124: 3597 – 3607

Velasco-Hernandez T, Soneji S, Hidalgo I, Erlandsson E, Cammenga J, Bryder D (2019) Hif-1α deletion may lead to adverse treatment effect in a mouse model of MLL-AF9-driven AML. *Stem Cell Reports* 12: 112 – 121

Verhagen HJMP, Smit MA, Rutten A, Denkers F, Poddighe PJ, Merle PA, Ossenkoppele GJ, Smit L (2016) Primary acute myeloid leukemia cells with overexpression of EVI-1 are sensitive to all-trans retinoic acid. *Blood* 127: 458 – 463

Vukovic M, Guitart AV, Sepulveda C, Villacreces A, O'Duibhir E, Panagopoulou TI, Ivens A, Menendez-Gonzalez J, Iglesias JM, Allen L *et al* (2015) Hif-1α and Hif-2α synergize to suppress AML development but are dispensable for disease maintenance. *J Exp Med* 212: 2223 – 2234

Wallace EM, Rizzi JP, Han G, Wehn PM, Cao Z, Du X, Cheng T, Czerwinski RM, Dixon DD, Goggin BS *et al* (2016) A small-molecule antagonist of HIF2α is efficacious in preclinical models of renal cell carcinoma. *Cancer Res* 76: 5491 – 5500

Wang Y, Liu Y, Malek SN, Zheng P, Liu Y (2011) Targeting HIF1α eliminates cancer stem cells in hematological malignancies. *Cell Stem Cell* 8: 399 – 411

Wierenga ATJ, Vellenga E, Schuringa JJ (2014) Convergence of hypoxia and TGFβ pathways on cell cycle regulation in human hematopoietic stem/progenitor cells. *PloS One* 9: 1 – 11

Wierenga ATJ, Cunningham A, Erdem A, Lopera NV, Brouwers-Vos AZ, Pruis M, Mulder AB, Günther UL, Martens JHA, Vellenga E *et al* (2019) HIF1/2-exerted control over glycolytic gene expression is not functionally relevant for glycolysis in human leukemic stem/progenitor cells. *Cancer Metab* 7: 11

Wigerup C, Påhlman S, Bexell D (2016) Therapeutic targeting of hypoxia and hypoxia-inducible factors in cancer. *Pharmacol Ther* 164: 152 – 169

Yan Q, Bartz S, Mao M, Li L, Kaelin WGJ (2007) The hypoxia-inducible factor 2alpha N-terminal and C-terminal transactivation domains cooperate to promote renal tumorigenesis in vivo. *Mol Cell Biol* 27: 2092 – 2102

Zhao J, Chen X, Song G, Zhang J, Liu H, Liu X (2017) Uhrf1 controls the self-renewal versus differentiation of hematopoietic stem cells by epigenetically regulating the cell-division modes. *Proc Natl Acad Sci U S A* 114: E142 – E151

License: This is an open access article under the terms of the Creative Commons Attribution License, which permits use, distribution and reproduction in any medium, provided the original work is properly cited.

Expanded View Figures

Figure EV1. Expression and silencing of HIF1α and HIF2α in AML cell lines.

A qPCR analysis of HIF1α and HIF2α in the indicated AML cell lines stably expressing shRNAs against HIF1α or HIF2α or a scrambled shRNA as control (shCTRL). Data are expressed as fold change in HIFα-silenced cells compared to shCTRL cells. Data represent mean ± SD of three biological replicates (Student's *t*-test).

B Cell proliferation of the indicated AML cell lines carrying shHIF1α, shHIF2α, or shCTRL. Values represent cell numbers normalized over day 0. Data represent mean ± SD of three biological replicates (Student's *t*-test).

C qPCR analysis of HIF1α and HIF2α in HL60 and Kasumi1 cells stably expressing shCTRL, shHIF1α or shHIF2α. Data are expressed as fold change in HIFα-silenced cells compared to shCTRL cells. Data represent mean ± SD of three biological replicates of cells at passages 4–10 (< P10) or passages 11–16 (> P10) (Student's *t*-test).

D Analysis of *HIF1A* and *EPAS1* (encoding HIF1α and HIF2α respectively) basal expression in the human AML cell lines utilized in this study. Data represent mean ± SD of three biological replicates.

E mRNA expression of *HIF1A* and *EPAS1* in 451 AML patients from the Oregon Health & Science University (OSHU) dataset (Tyner *et al*, 2018). Data are expressed as normalized RPKM (Reads Per Kilobase Million), and were obtained from the cBioportal database (Cerami *et al*, 2012). Data represent mean ± SD of 451 biological replicates.

F Immunoblot analysis showing silencing efficiency of two independent shRNAs against HIF1α (shHIF1α#1 and shHIF1α#2) and HIF2α (shHIF2α#1 and shHIF2α#2), or a scrambled shRNA as control (shCTRL) in Kasumi1 cells. shHIF1α#1 and shHIF2α#2 are shRNAs utilized in the main figures. α-tubulin was used as loading control.

G qPCR analysis of HIF1α and HIF2α upon HIFα-specific silencing and compared to shCTRL in Kasumi1 (left graph) and HL60 (right graph) cells. Data represent mean ± SD of three biological replicate (Student's *t*-test).

H Colony forming capacity of Kasumi1 (left graph) and HL60 (right graph) cells expressing shHIF1α#1, shHIF1α#2, shHIF2α#1, shHIF2α#2, or shCTRL. Shown is the average number of colonies/field in 20 fields (10x objective). Data represent mean ± SD of three biological replicates (Student's *t*-test).

I Percentages of $CD11b^+$ in cells described in (H). Data represent mean ± SD of three biological replicates (Student's *t*-test).

© 2023 The Authors

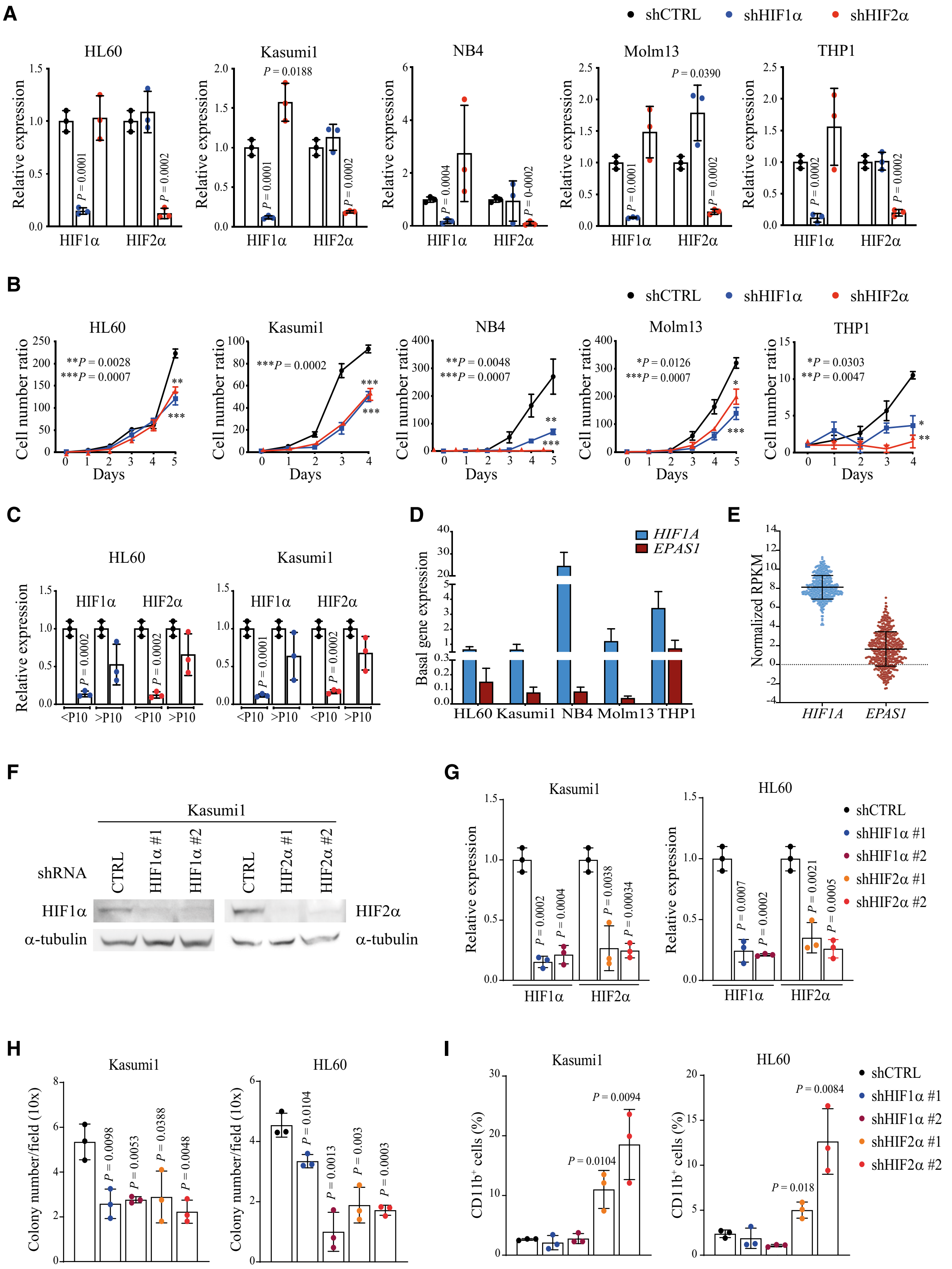

A
shCTRL
shHIF1α
shHIF2α
HL60
Kasumi1
NB4
Molm13
THP1
Relative expression
HIF1α
HIF2α
B
Cell number ratio
Days
C
<P10
>P10
D
HIF1A
EPAS1
Basal gene expression
E
Normalized RPKM
F
shRNA
CTRL
HIF1α #1
HIF1α #2
HIF2α #1
HIF2α #2
α-tubulin
G
shHIF1α #1
shHIF1α #2
shHIF2α #1
shHIF2α #2
H
Colony number/field (10x)
I
CD11b+ cells (%)

Figure EV1.

Figure EV2. Global changes in gene expression, H3K27me3 and chromatin accessibility upon HIF1α or HIF2α silencing in AML cells.

A Unsupervised hierarchical clustering of differentially expressed genes (DEGs) in HL60 and Kasumi1 cells following HIF1α or HIF2α silencing. A red-blue color scale was used to reflect normalized RPKM, with red indicating genes with higher expression and blue indicating genes with lower expression. Each column represents the average of two independent experiments.

B Venn diagrams showing the number of DEGs commonly upregulated and downregulated in HL60 and Kasumi1 cells upon shHIF1α (top) or shHIF2α (bottom). Common/total deregulated genes in each cell line are indicated as percentages.

C qPCR analysis of the indicated HIF2α-regulated genes in NB4 (left graph), Molm13 (middle graph) and THP1 (right graph) cells upon HIF2α silencing. Values indicate fold change in gene expression compared to shCTRL cells. Data represent mean ± SD of three biological replicates (Student's *t*-test).

D, E Gene set enrichment analysis of genes upregulated upon HIF2α silencing and showing unique H3K27me3 peaks in shCTRL (D) or downregulated upon HIF2α silencing and showing unique H3K27me3 peaks in shHIF2α (E) Kasumi1 cells. Indicated are the terms most significantly enriched in the following libraries: gene ontology (GO) biological process, GO molecular function, GO cellular component, Bioplanet, Reactome, and Hallmarks of cancer. Dot sizes represent the number of genes in each term, and colors indicate Enrichment Scores expressed as $-\log_{10}$ (*P*-value).

F Genome browser view of normalized counts of ATAC-seq profiles at the regulatory regions of the myeloid differentiation genes *ITGB2*, *CD53*, and *IFI16*. Open chromatin peaks of Kasumi1 cells are indicated in red for shCTRL and green for shHIF2α. Triplicate experiments and a representative alignment (normalized CPM values) of each condition are shown. Open chromatin peaks of six primary AML cells (obtained from GSE197416; Data ref: Gambacorta *et al*, 2022b) are indicated in orange and a representative alignment is shown. Lower panels represent the corresponding gene annotation from GrCh38 human reference.

 © 2023 The Authors

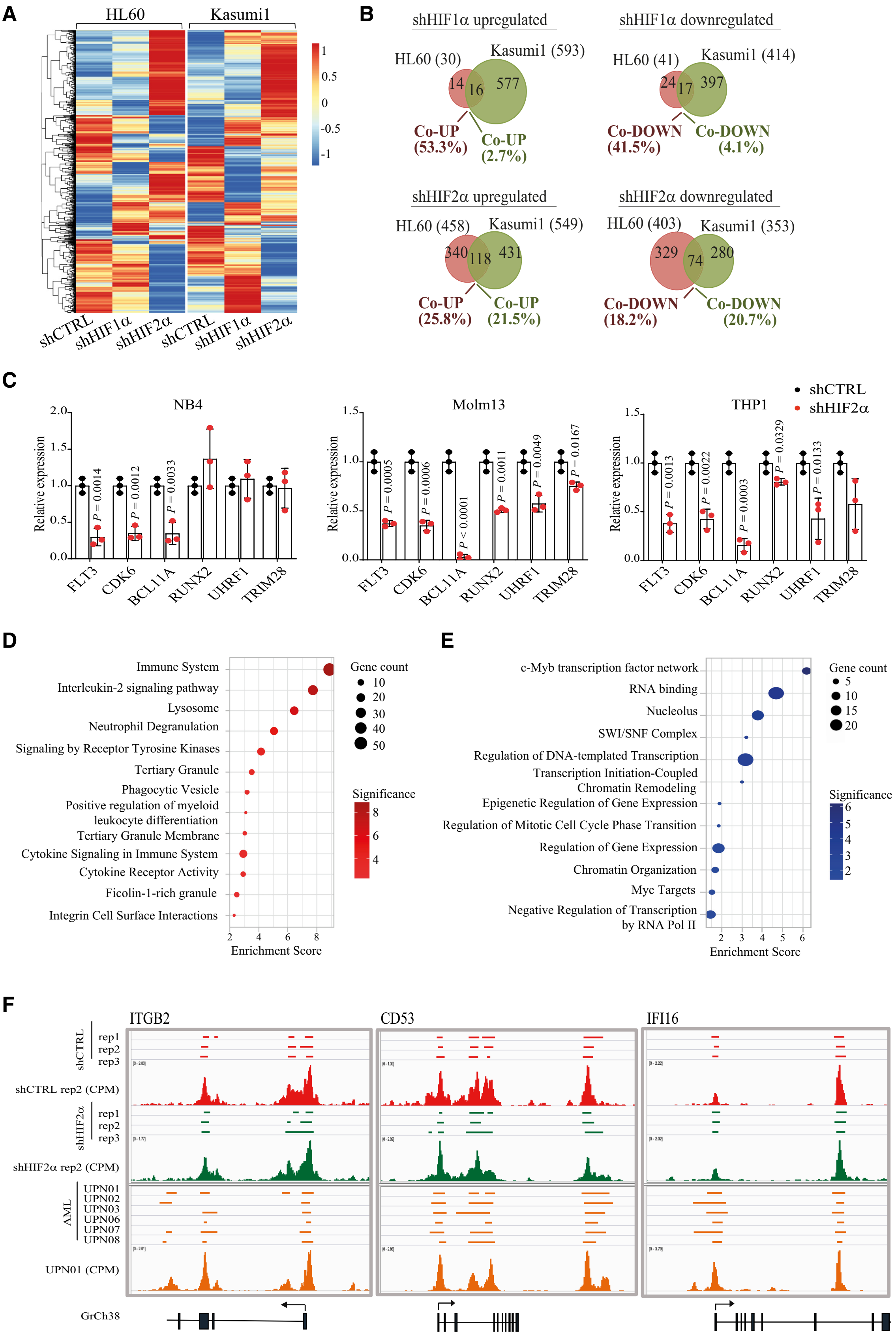
A
HL60
Kasumi1
shCTRL
shHIF1α
shHIF2α
shCTRL
shHIF1α
shHIF2α
B
shHIF1α upregulated
HL60 (30)
Kasumi1 (593)
14
16
577
Co-UP (53.3%)
Co-UP (2.7%)
shHIF1α downregulated
HL60 (41)
Kasumi1 (414)
24
17
397
Co-DOWN (41.5%)
Co-DOWN (4.1%)
shHIF2α upregulated
HL60 (458)
Kasumi1 (549)
340
118
431
Co-UP (25.8%)
Co-UP (21.5%)
shHIF2α downregulated
HL60 (403)
Kasumi1 (353)
329
74
280
Co-DOWN (18.2%)
Co-DOWN (20.7%)
C
NB4
Molm13
THP1
shCTRL
shHIF2α
Relative expression
P = 0.0014
P = 0.0012
P = 0.0033
P = 0.0005
P = 0.0006
P < 0.0001
P = 0.0011
P = 0.0049
P = 0.0167
P = 0.0013
P = 0.0022
P = 0.0003
P = 0.0329
P = 0.0133
FLT3
CDK6
BCL11A
RUNX2
UHRF1
TRIM28
D
Immune System
Interleukin-2 signaling pathway
Lysosome
Neutrophil Degranulation
Signaling by Receptor Tyrosine Kinases
Tertiary Granule
Phagocytic Vesicle
Positive regulation of myeloid leukocyte differentiation
Tertiary Granule Membrane
Cytokine Signaling in Immune System
Cytokine Receptor Activity
Ficolin-1-rich granule
Integrin Cell Surface Interactions
Gene count
Significance
Enrichment Score
E
c-Myb transcription factor network
RNA binding
Nucleolus
SWI/SNF Complex
Regulation of DNA-templated Transcription
Transcription Initiation-Coupled Chromatin Remodeling
Epigenetic Regulation of Gene Expression
Regulation of Mitotic Cell Cycle Phase Transition
Regulation of Gene Expression
Chromatin Organization
Myc Targets
Negative Regulation of Transcription by RNA Pol II
Gene count
Significance
Enrichment Score
F
ITGB2
CD53
IFI16
shCTRL
rep1
rep2
rep3
shCTRL rep2 (CPM)
shHIF2α
shHIF2α rep2 (CPM)
AML
UPN01
UPN02
UPN03
UPN06
UPN07
UPN08
UPN01 (CPM)
GrCh38

Figure EV2.

Figure EV3. *In vitro* and *ex vivo* treatment of AML cell lines and PDX-derived cells with the specific HIF2α inhibitor PT2385.

A Cell numbers of NB4, Molm13 and THP1 cells 2 days after treatment with PT2385 at the indicated doses and compared to vehicle treated cells. Data represent mean ± SD of three biological replicates (one-way ANOVA followed by Tukey's multiple comparison test).

B Percentages of $CD11b^+$ NB4, Molm13 and THP1 cells 2 days after treatment with PT2385 at the indicated doses and compared to vehicle treated cells. Data represent mean ± SD of three biological replicates (one-way ANOVA followed by Tukey's multiple comparison test).

C Relative proportion of live ($AnnexinV^-$) and apoptotic ($AnnexinV^+$) cells after treatment with PT2385 at the indicated doses for 2 and 6 days in HL60 (upper graphs) and Kasumi1 (lower graphs) cells. Data represent mean ± SD of three biological replicates (Student's *t*-test).

D qPCR analysis of the indicated genes in NB4 (upper graph), Molm13 (middle graph) and THP1 (lower graph) cells 2 days after treatment with 200 μM PT2385. Values are represented as fold change in gene expression compared to vehicle treated cells. Data represent mean ± SD of three biological replicates (Student's *t*-test).

E Percentages of $CD11b^+$ NB4 (upper graph), Molm13 (middle graph) and THP1 (lower graph) cells at the indicated days (D2, D4, D6) after treatment with 50 μM PT2385 and compared to vehicle treated cells. Data represent mean ± SD of three biological replicates (Student's *t*-test).

F Percentages of $CD11b^+$ AML-01 cells isolated from the bone marrow of PDX mice and treated *ex vivo* with 100 μM PT2385 for 4 days. Data represent mean ± SD of three biological replicates (Student's *t*-test).

G qPCR analysis of the indicated genes in AML-01 cells upon *ex vivo* treatment with 100 μM PT2385. Values are represented as fold changes in gene expression compared to control vehicle treated cells. Data represent mean ± SD of three biological replicates (Student's *t*-test).

 © 2023 The Authors

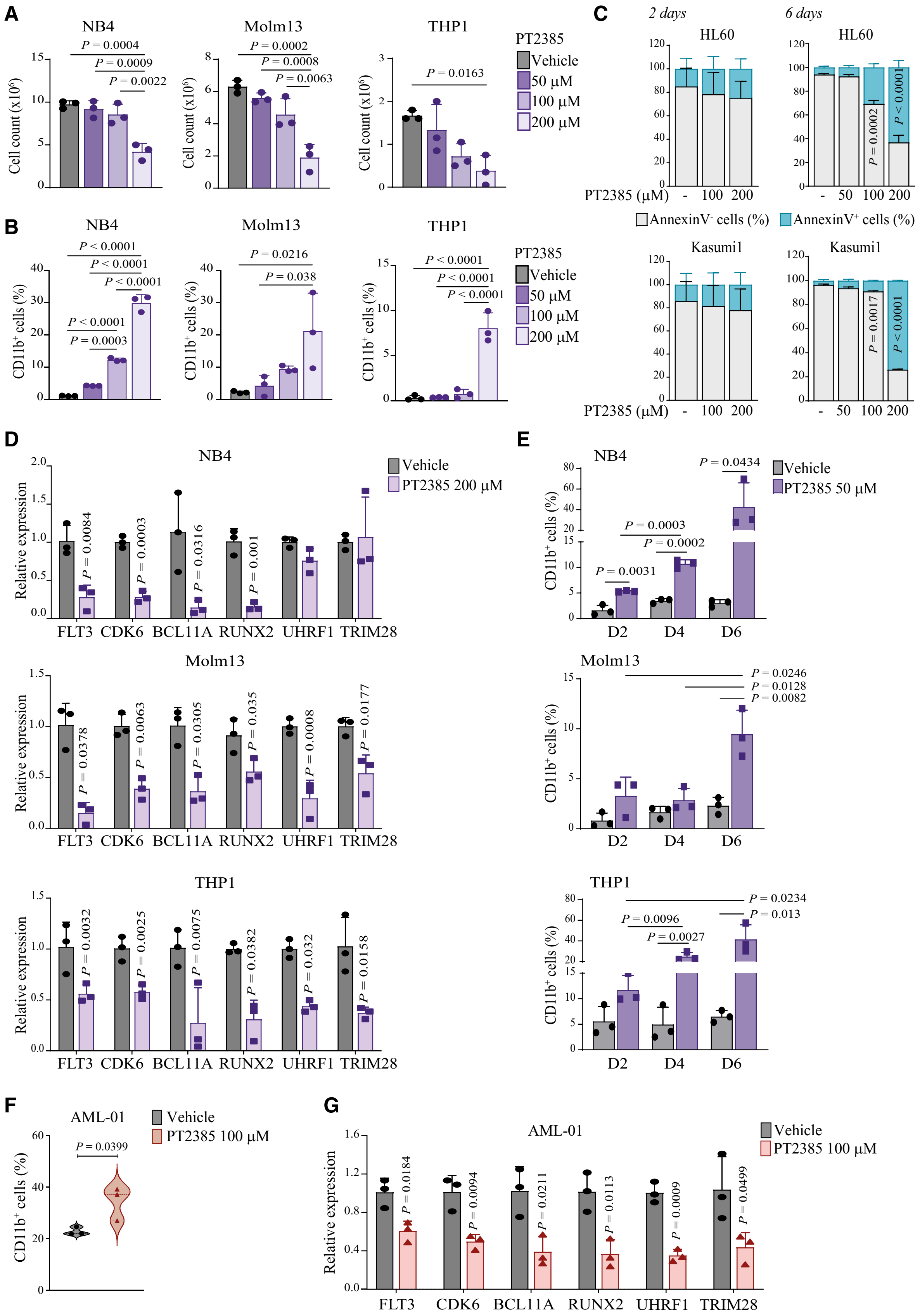
A
NB4
Molm13
THP1
Cell count (x10⁶)
P = 0.0004
P = 0.0009
P = 0.0022
P = 0.0002
P = 0.0008
P = 0.0063
P = 0.0163
PT2385
Vehicle
50 μM
100 μM
200 μM
B
CD11b⁺ cells (%)
P < 0.0001
P = 0.0003
P = 0.0216
P = 0.038
C
2 days
6 days
HL60
Kasumi1
PT2385 (μM)
- 50 100 200
P = 0.0002
P < 0.0001
P = 0.0017
AnnexinV⁻ cells (%)
AnnexinV⁺ cells (%)
D
Relative expression
PT2385 200 μM
FLT3 CDK6 BCL11A RUNX2 UHRF1 TRIM28
P = 0.0084
P = 0.0003
P = 0.0316
P = 0.001
P = 0.0378
P = 0.0063
P = 0.0305
P = 0.035
P = 0.0008
P = 0.0177
P = 0.0032
P = 0.0025
P = 0.0075
P = 0.0382
P = 0.032
P = 0.0158
E
PT2385 50 μM
D2 D4 D6
P = 0.0434
P = 0.0003
P = 0.0002
P = 0.0031
P = 0.0246
P = 0.0128
P = 0.0082
P = 0.0234
P = 0.013
P = 0.0096
P = 0.0027
F
AML-01
PT2385 100 μM
P = 0.0399
G
P = 0.0184
P = 0.0094
P = 0.0211
P = 0.0113
P = 0.0009
P = 0.0499

Figure EV3.

Figure EV4. *In vivo* treatment of PDX AML models with compounds inhibiting HIF2α.

A Percentages of leukemic cells ($hCD45^+$) expressing AnnexinV in bone marrow (BM, left graph), spleen (SP, middle graph) and peripheral blood (PB, right graph) of mice transplanted with AML-01 cells and treated with 100 mg/kg PT2385 or vehicle. Data represent mean ± SD of four biological replicates.

B qPCR analysis of HIF2α and HIF2α-regulated genes in AML-01 cells isolated from the bone marrow of leukemic mice treated with 100 mg/kg PT2385 (n = 4) or vehicle (n = 3). Values indicate fold change in gene expression compared to control vehicle treated cells and represent mean ± SD of 3/4 biological replicates (Student's t-test).

C Percentages of apoptotic leukemic cells ($AnnexinV^+hCD45^+$) in bone marrow (BM), spleen (SP) and peripheral blood (PB) of mice transplanted with AML-01 (left graph) or AML-02 (right graph) and treated with EZN-2208. Data are represented in box and whisker plots where the central band denotes the median value, box contains interquartile ranges, while whiskers mark minimum and maximum values. All biological replicates are shown (n = 7 in AML-01, n = 4 in AML-02).

D Spleen weight (left graph) and percentages of leukemic cells ($hCD45^+$, right graph) in BM, SP, and PB of mice transplanted with AML-01 and treated with EZN-2208 as in (C). Data are represented in box and whisker plots where the central band denotes the median value, box contains interquartile ranges, while whiskers mark minimum and maximum values. All biological replicates are shown (n = 7, Student's t-test).

E Spleen weight (left graph) and percentages of leukemic cells ($hCD45^+$, right graph) in BM, SP, and PB of mice transplanted with AML-02 and treated with EZN-2208 as in (C). Data are represented in box and whisker plots where the central band denotes the median value, box contains interquartile ranges, while whiskers mark minimum and maximum values. All biological replicates are shown (n = 7, Student's t-test).

F Percentages of leukemic AML-01 and AML-02 cells expressing CD11b ($hCD11b^+hCD45^+$) in BM, SP, and PB of mice transplanted with AML-01 (left graph) and AML-02 (right graph) and treated with EZN-2208. Data are represented in box and whisker plots where the central band denotes the median value, box contains interquartile ranges, while whiskers mark minimum and maximum values. All biological replicates are shown (n = 7 in AML-01, n = 4 in AML-02, Student's t-test).

G qPCR of the indicated genes in cells isolated from BM of vehicle (n = 3) and EZN-2208 treated (n = 4) AML-01 transplanted mice. Values indicate fold changes in gene expression of EZN-2208 treated cells compared to vehicle treated control cells. Data represent mean ± SD of 3/4 biological replicates (Student's t-test).

 © 2023 The Authors

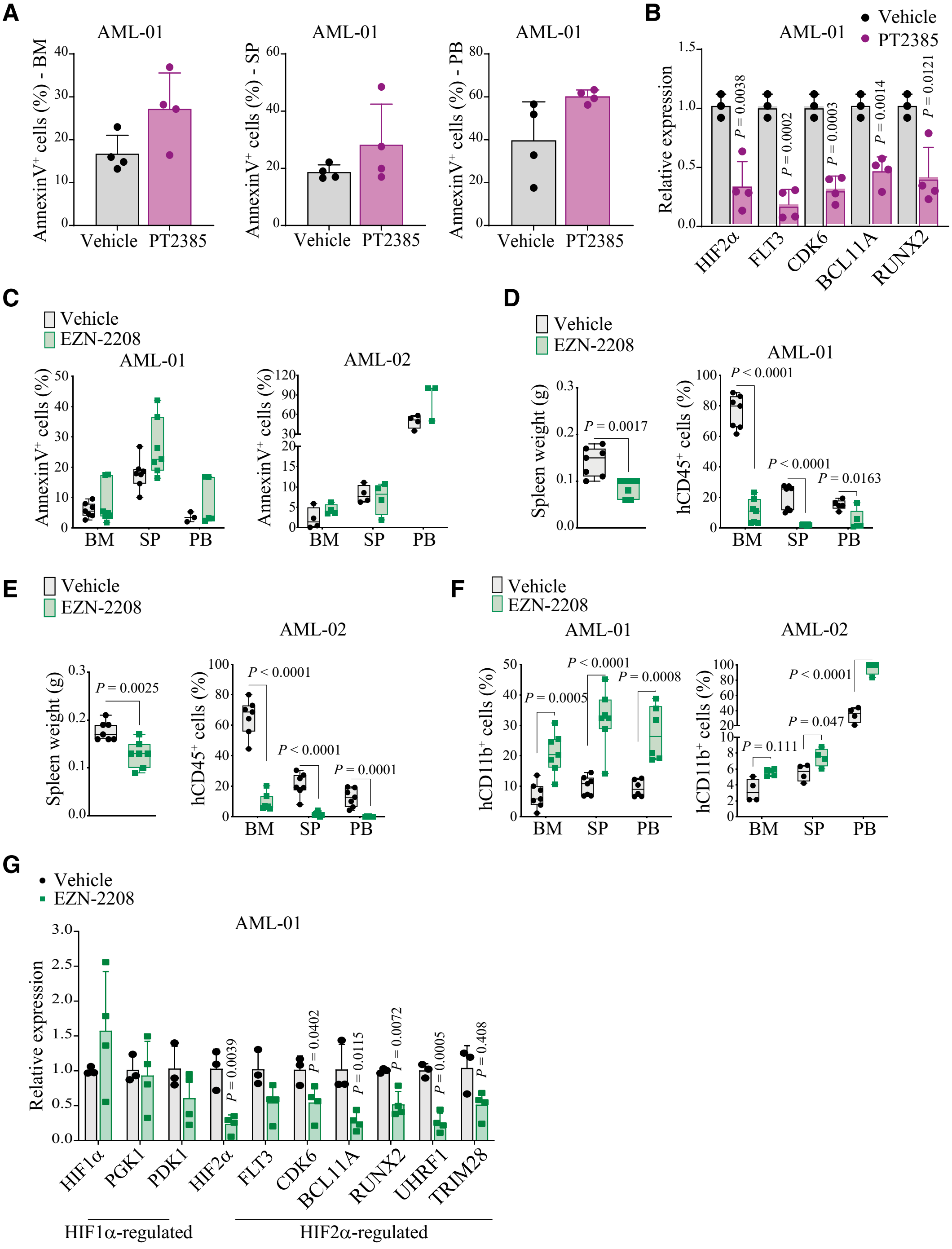
A
AML-01
AnnexinV+ cells (%) - BM
Vehicle
PT2385
AnnexinV+ cells (%) - SP
AnnexinV+ cells (%) - PB
B
AML-01
Vehicle
PT2385
Relative expression
P = 0.0038
P = 0.0002
P = 0.0003
P = 0.0014
P = 0.0121
HIF2α
FLT3
CDK6
BCL11A
RUNX2
C
Vehicle
EZN-2208
AML-02
BM
SP
PB
D
Spleen weight (g)
P = 0.0017
hCD45+ cells (%)
P < 0.0001
P = 0.0163
E
P = 0.0025
P = 0.0001
F
hCD11b+ cells (%)
P = 0.0005
P = 0.0008
P = 0.047
P = 0.111
G
AML-01
HIF1α
PGK1
PDK1
UHRF1
TRIM28
P = 0.0039
P = 0.0402
P = 0.0115
P = 0.0072
P = 0.0005
P = 0.408
HIF1α-regulated
HIF2α-regulated

Figure EV4.

Figure EV5. Combination of HIF2α inhibition and ATRA increases AML differentiation in NB4, Molm13, and THP1 cells.

A Percentages of $CD11b^+$ NB4 (left graph), Molm13 (middle graph) and THP1 (right graph) cells with shRNAs against HIF1α, HIF2α, or a scrambled shRNA as control (shCTRL) treated with 1 μM ATRA for 2 days. Data represent mean ± SD of three biological replicates (one-way ANOVA followed by Tukey's multiple comparison test).

B Percentages of $CD11b^+$ NB4 (left graph), Molm13 (middle graph) and THP1 (right graph) cells following treatment with 50 μM PT2385, 1 μM ATRA, or combination for 4 days. Data represent mean ± SD of three biological replicates (one-way ANOVA followed by Tukey's multiple comparison test).

C Percentages of NB4 (left graph), Molm13 (right graph) and THP1 (lower graph) cells in the indicated phases of the cell cycle following treatment with 50 μM PT2385, 1 μM ATRA, or combination for 4 days. Data represent mean ± SD of three biological replicates (Student's *t*-test).

 © 2023 The Authors

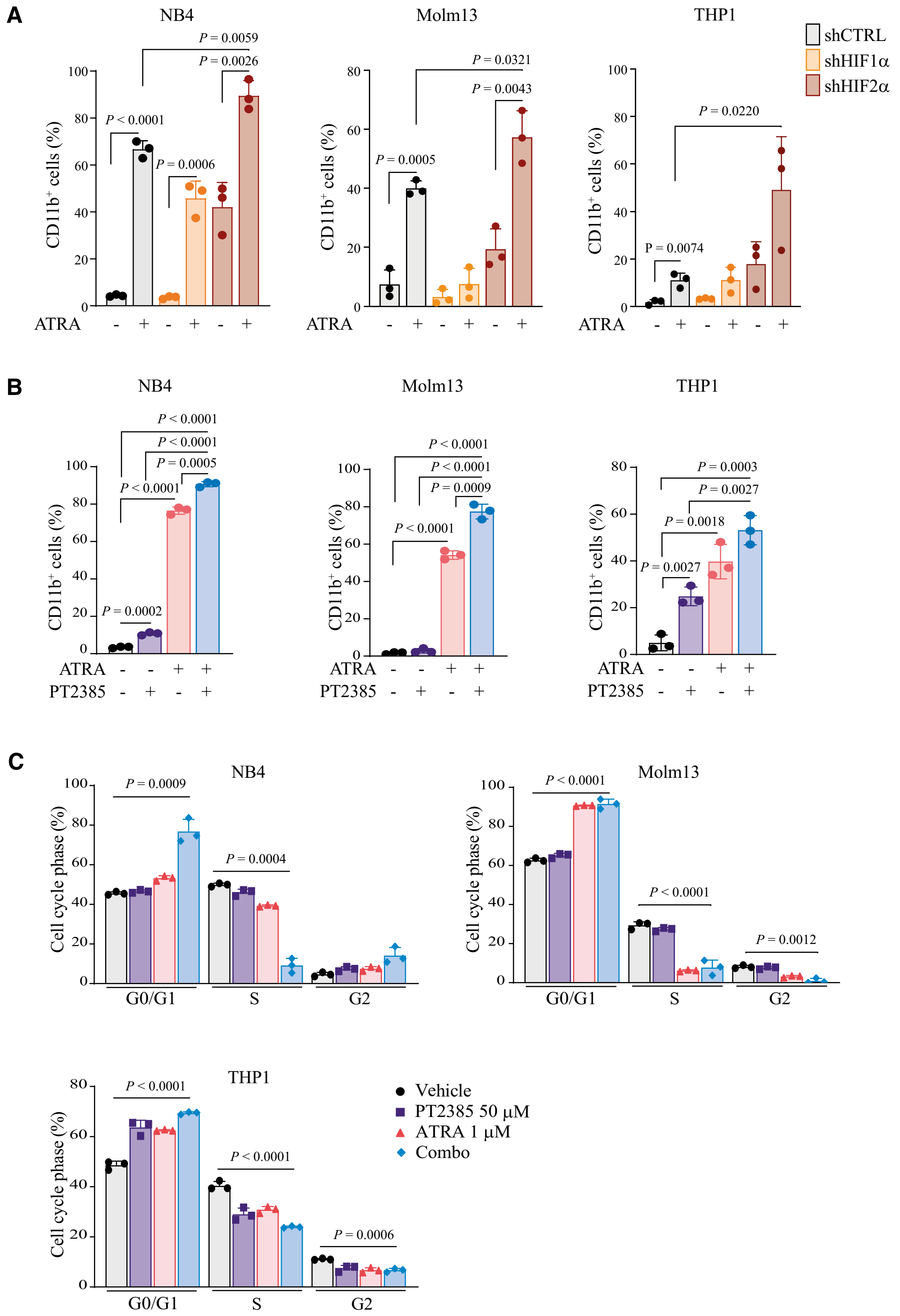
A
NB4
Molm13
THP1
shCTRL
shHIF1α
shHIF2α
CD11b+ cells (%)
ATRA
P < 0.0001
P = 0.0006
P = 0.0059
P = 0.0026
P = 0.0005
P = 0.0321
P = 0.0043
P = 0.0074
P = 0.0220
B
PT2385
P = 0.0002
P = 0.0005
P = 0.0009
P = 0.0003
P = 0.0027
P = 0.0018
C
Cell cycle phase (%)
G0/G1
S
G2
P = 0.0009
P = 0.0004
P = 0.0012
P = 0.0006
Vehicle
PT2385 50 µM
ATRA 1 µM
Combo

Figure EV5.

Table of contents to the appendix file

Contents	Page
Appendix Figure S1	2
Appendix Figure S2	3
Appendix Figure S3	4
Appendix Figure S4	5
Appendix Figure Legends	6-8
Appendix Table S1	9-12
Appendix Table S2	13-15
Appendix Table S3	16
Appendix Table S4	17

Appendix Figure S1

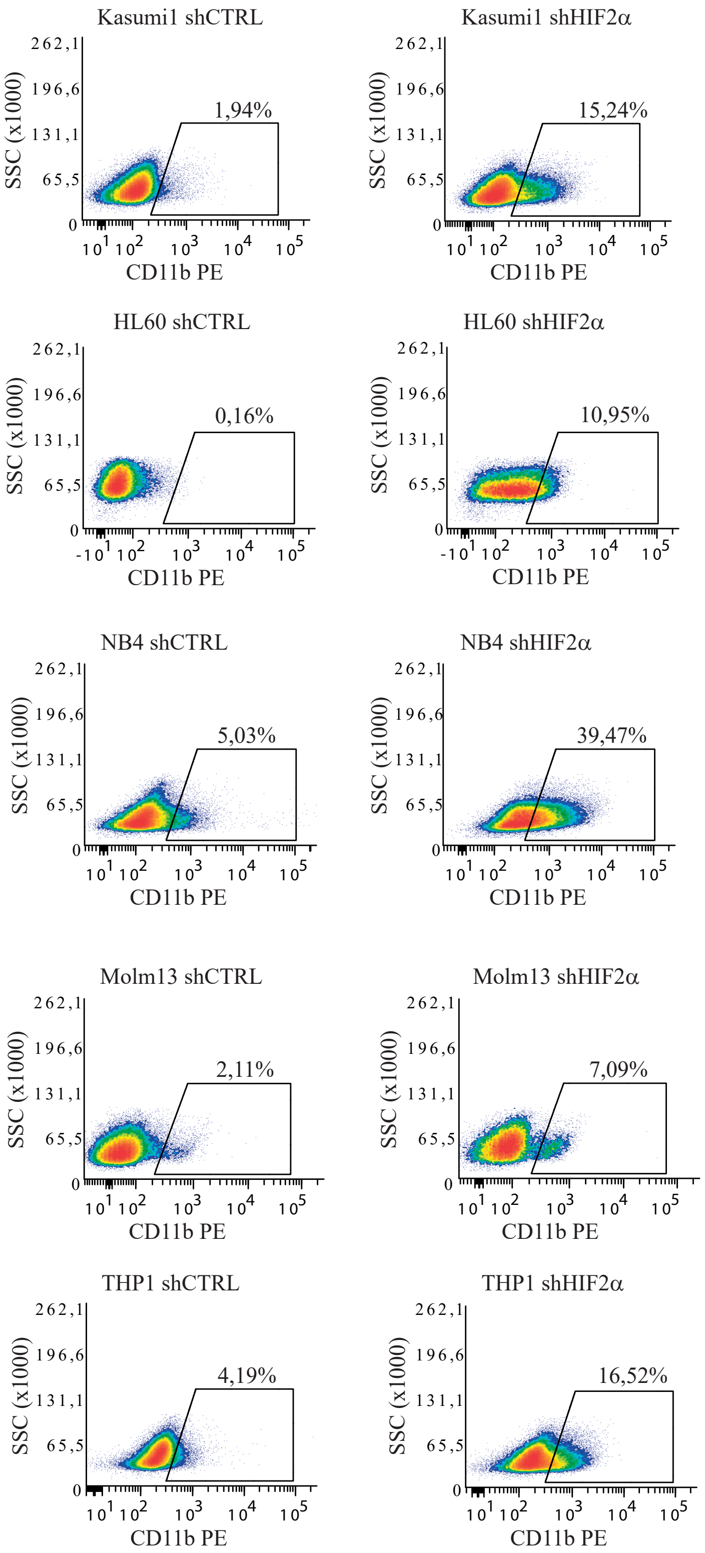

Appendix Figure S2

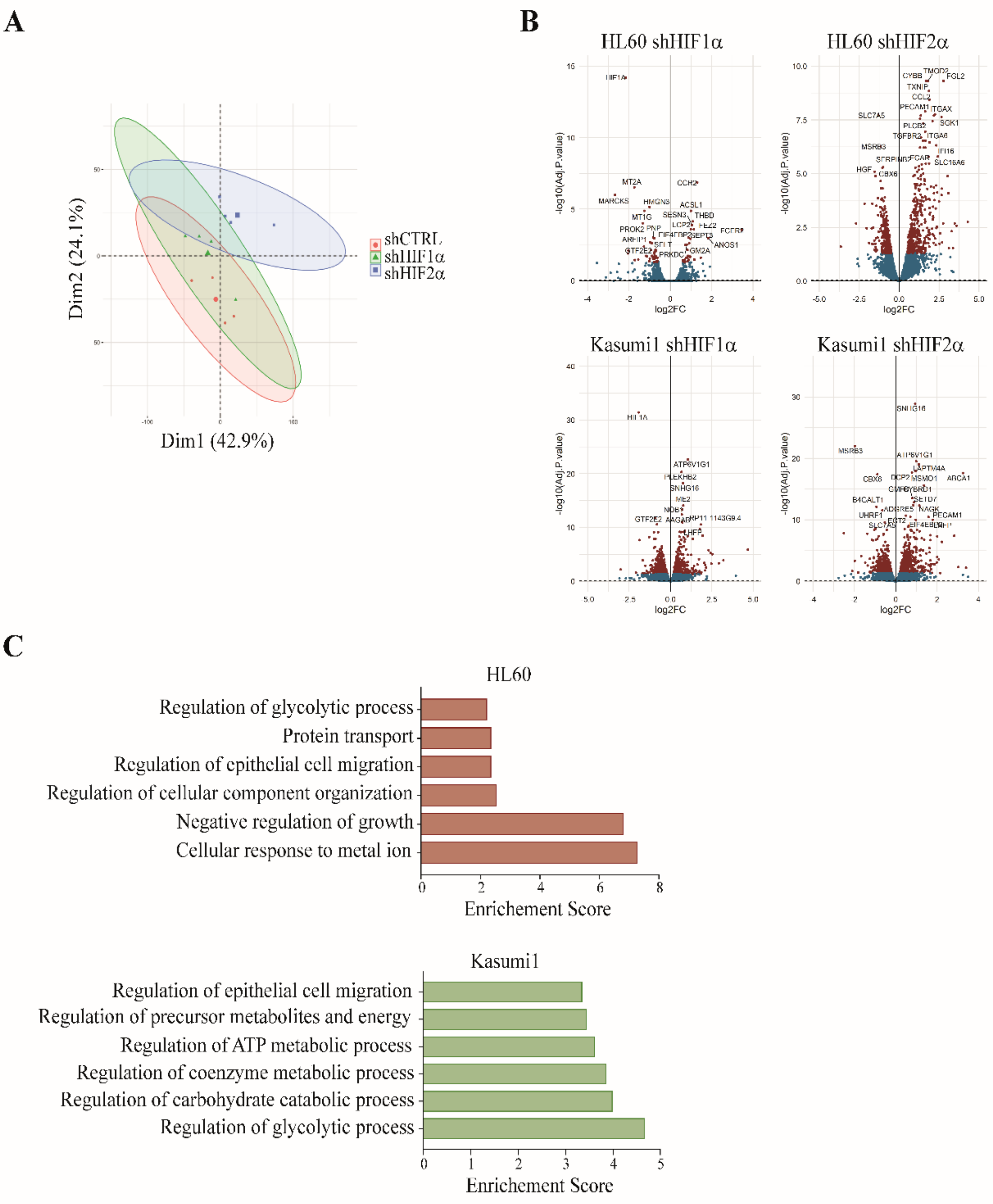

Appendix Figure S3

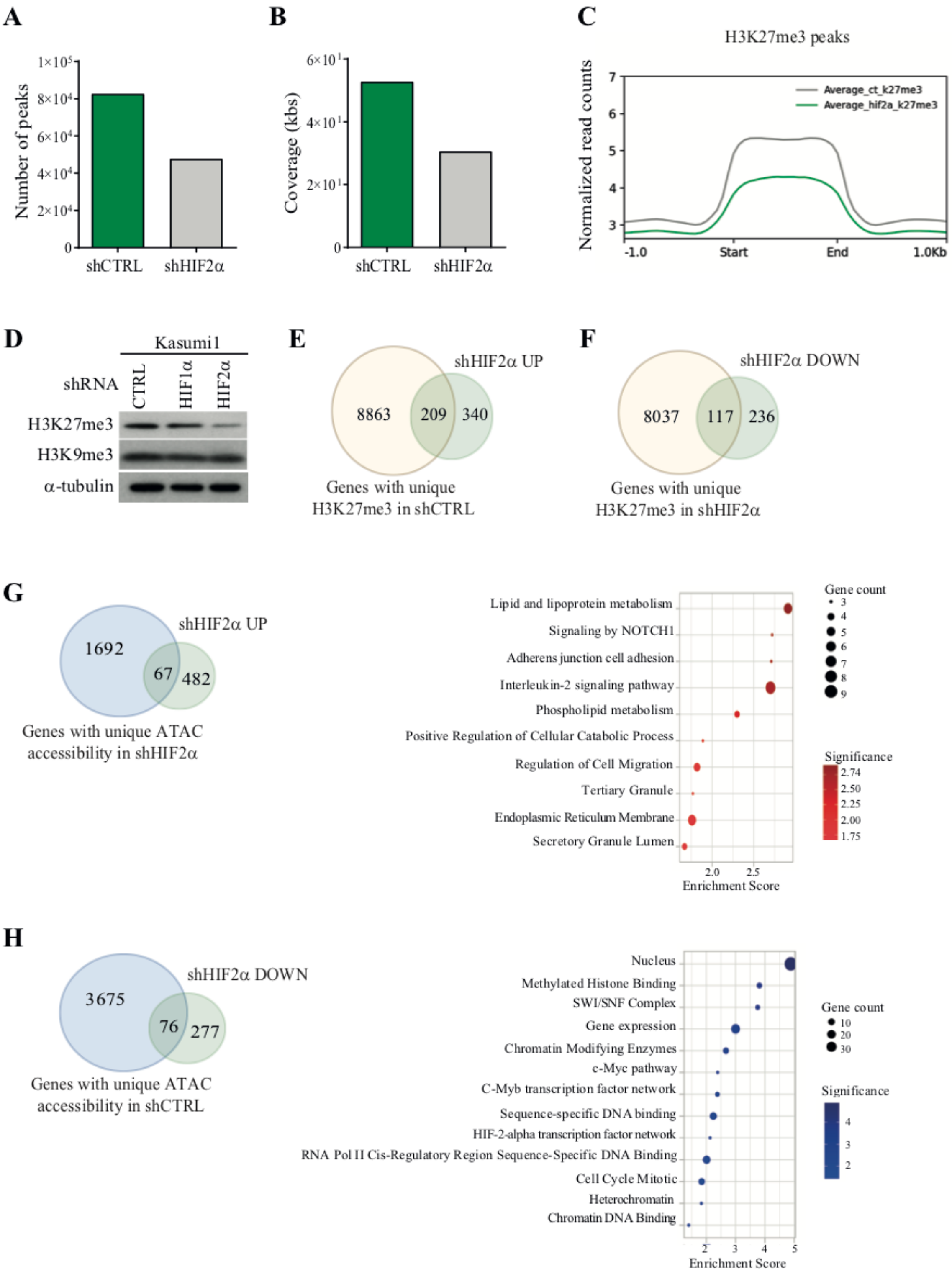

Appendix Figure S4

A

PDX	Chromosomal abnormality	Main genetic lesions	Immunophenotype
AML-01	Normal cytogenetic	NPM1, DNMT3A, FLT3-ITD, IDH1	CD4+/CD7+/CD11b+/CD11c+/CD13+/ CD33+/CD38+/CD45RA+/CD117+
AML-02	Normal cytogenetic	NPM1, DNMT3A, FLT3-ITD	CD24+/CD7+/CD11b+/CD13+/CD33+/ CD34+/CD38+/CD45RA+/CD117+

B

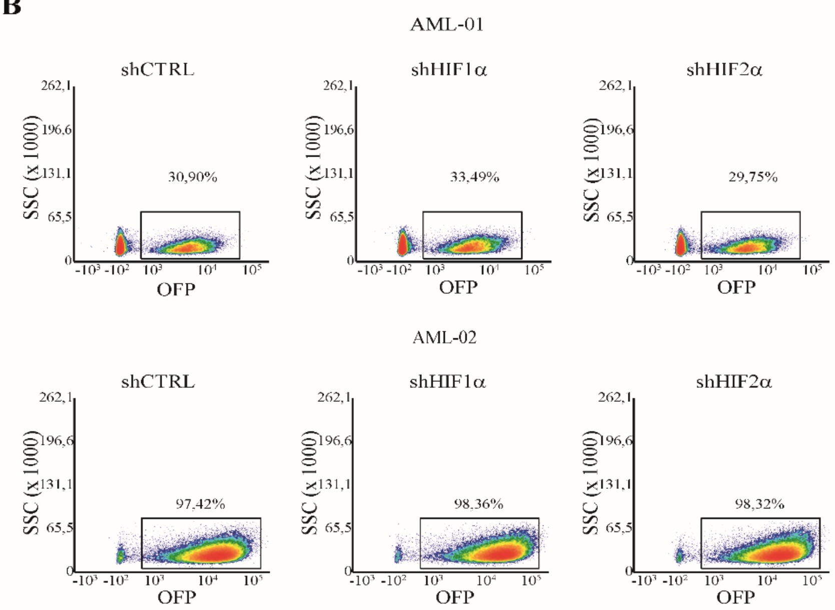

C

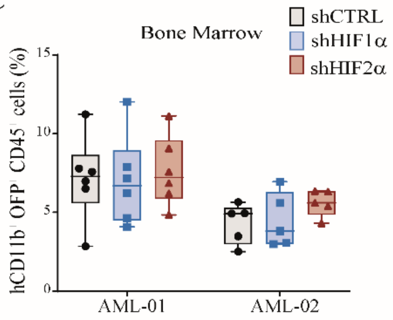

D

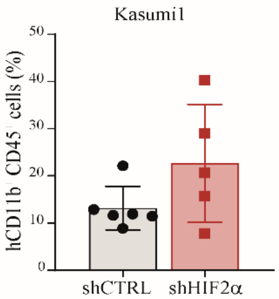

Appendix Figure Legends

Appendix Figure S1. Analysis of CD11b expression upon HIF2α silencing in AML cell lines. Representative flow cytometry profiles of CD11b and side scatter (SSC) in Kasumi1 and HL60 cell lines with control shRNA (shCTRL) or shRNA against HIF2α (shHIF2α). Gates were set over PE isotype control antibody.

Appendix Figure S2. Global changes in gene expression upon HIF1α or HIF2α silencing in AML cells. A. Principal component analysis (PCA) on differentially expressed genes (DEGs) in shHIF1α, shHIF2α, and shCTRL HL60 and Kasumi1 cells. Ellipses identify clusters, the centroid represents the barycenter of the points belonging to the same cluster. **B.** Volcano plots depicting DEGs in shHIF1α and shHIF2α HL60 and Kasumi1 cells with respect to shCTRL cells. Red and blue dots correspond respectively to significantly and non-significantly deregulated genes. A significant threshold of 0.05, adjusting the p-value by FDR (False Discovery Rates), was established to identify DEGs. **C** Representative Gene Ontology (GO) terms within the most significant families of downregulated genes in shHIF1α HL60 and Kasumi1 cells. Data are presented as Enrichment Score ($-\log_{10}$(p-value)) over shCTRL cells.

Appendix Figure S3. Global changes in H3K27me3 and chromatin accessibility via ATAC-seq upon HIF2α silencing in Kasumi1 cells. A, B. Number and coverage in kilobases (kbs) of H3K27me3 peaks in shCTRL and shHIF2α cells. **C.** H3K27me3 levels in peaks identified in shCTRL and shHIF2α cells **D.** Immunoblot analysis of H3K27me3 and H3K9me3 in Kasumi1 cells carrying a scrambled shRNA as control (CTRL) or shRNAs against HIF1α and HIF2α. α-tubulin was used as loading control. **E.** Overlap of genes linked to unique H3K27me3 peaks in shCTRL cells and genes upregulated (UP) upon HIF2α silencing

(shHIF2α). **F.** Overlap of genes mapping to unique H3K27me3 peaks in shHIF2α cells and genes downregulated (DOWN) upon HIF2α silencing (shHIF2α). **G.** Overlap of genes mapping to unique ATAC peaks in HIF2α silenced cells and genes upregulated (UP) upon HIF2α silencing (shHIF2α, left Venn diagram). Gene set enrichment analysis of genes regulated by HIF2α and showing changes in chromatin accessibility upon HIF2α silencing (right plot). **H.** Overlap of genes mapping to unique ATAC peaks in control cells and genes downregulated (DOWN) upon HIF2α silencing (shHIF2α, left Venn diagram). Gene set enrichment analysis of genes regulated by HIF2α and showing changes in chromatin accessibility upon HIF2α silencing (right plot). In **G** and **H**, the following libraries were used: gene ontology (GO) biological process, GO molecular function, GO cellular component, Bioplanet, Reactome, and Hallmarks of cancer. Dot sizes represent the number of genes in each term, and colors indicate Enrichment Scores expressed as $-\log_{10}$(p-value).

Appendix Figure S4. Silencing of HIFα factors in PDX-derived AML cells. A. Genetic and immunophenotypic features of the two AML PDX models utilized in this work. **B.** FACS analysis performed at 96 hours after infection of AML-01 and AML-02 cells with lentiviral vectors carrying specific shRNAs and orange fluorescent protein (OFP). Shown is the transduction efficiency in AML-01 and AML-02 cells. Data are representative of one out of three experiments with similar results. **C.** Percentage of leukemic cells expressing the myeloid differentiation marker CD11b ($hCD11b^{+}OFP^{+}CD45^{+}$) in the bone marrow of AML-01 (n=6) and AML-02 (n=5) mice carrying shCTRL, shHIF1α, or shHIF2α. Data are shown in box and whisker plots where the central band denotes the median value, box contains interquartile ranges, while whiskers mark minimum and maximum values. All biological replicates are shown (n=5/6). **D.** Percentage of leukemic cells expressing the myeloid differentiation marker

CD11b (hCD11b$^+$CD45$^+$) in Kasumi1 cells carrying shCTRL (n=6) or shHIF2α (n=5) injected subcutaneously.

Appendix Table S1. List of 118 genes commonly upregulated upon HIF2α silencing in HL60 and Kasumi1 cells.

Gene Symbol	HL60 Log_2FC	HL60 padj	Kasumi1 Log_2FC	Kasumi1 padj	Description
ACSL4	0,73	1,29E-02	0,52	1,62E-05	acyl-CoA synthetase long chain family member 4
ADAP2	1,71	8,41E-03	1,4	5,32E-03	ArfGAP with dual PH domains 2
ADGRE2	1,13	1,92E-04	0,57	2,24E-02	adhesion G protein-coupled receptor E2
AIF1	0,56	4,90E-02	0,66	7,32E-04	allograft inflammatory factor 1
ALDH3A2	0,7	1,10E-02	0,73	4,38E-09	aldehyde dehydrogenase 3 family member A2
ANOS1	1,62	1,35E-03	2,24	4,10E-07	anosmin 1
ANXA5	0,65	1,29E-02	0,38	2,77E-02	annexin A5
APLP2	0,59	7,18E-03	0,61	4,17E-03	amyloid beta precursor like protein 2
APOL1	1,32	1,64E-02	1	2,54E-02	apolipoprotein L1
ASPH	1,04	3,25E-05	0,99	7,38E-06	aspartate beta-hydroxylase
ATG9A	0,92	4,19E-02	1,02	3,43E-06	autophagy related 9A
ATHL1	1,25	5,83E-03	0,68	3,51E-02	protein-glucosylgalactosylhydroxylysine glucosidase
ATP6V1G1	0,84	1,42E-03	0,98	3,13E-20	ATPase H+ transporting V1 subunit G1
CCR1	1,42	1,31E-04	0,88	2,28E-04	C-C motif chemokine receptor 1
CCSER2	0,96	2,17E-02	0,52	3,95E-02	coiled-coil serine rich protein 2
CD300C	0,99	2,68E-02	0,61	1,25E-02	CD300c molecule
CD4	0,65	1,48E-02	0,35	2,25E-02	CD4 molecule
CD53	1,04	4,76E-05	0,78	4,17E-03	CD53 molecule
CREB3L2	0,73	3,73E-03	0,69	8,09E-07	cAMP responsive element binding protein 3 like 2
CTSB	0,6	2,89E-02	0,59	4,32E-05	cathepsin B
CTSZ	1,02	6,14E-04	0,83	1,21E-04	cathepsin Z
CYB5R1	0,61	4,92E-02	0,48	1,50E-02	cytochrome b5 reductase 1
CYBB	1,67	4,90E-10	0,93	4,57E-04	cytochrome b-245, beta polypeptide
CYBRD1	0,79	5,58E-03	1,37	2,63E-16	cytochrome b reductase 1
CYTH4	0,8	3,74E-02	0,76	2,17E-03	cytohesin 4
DCP2	0,75	3,47E-03	0,79	1,94E-18	decapping mRNA 2
DEF8	0,73	2,80E-02	0,58	2,25E-05	differentially expressed in FDCP 8 homolog
EID1	0,57	1,59E-02	0,32	1,63E-02	EP300 interacting inhibitor of differentiation 1

EIF4EBP2	1,02	5,20E-06	0,98	9,74E-11	eukaryotic translation initiation factor 4E binding protein 2
ENTPD5	0,83	3,84E-02	0,56	4,64E-02	ectonucleoside triphosphate diphosphohydrolase 5
EVI2A	1,51	1,00E-05	0,62	4,85E-02	ecotropic viral integration site 2A
FADS1	0,63	8,16E-03	0,85	3,48E-04	fatty acid desaturase 1
FADS3	1,15	4,84E-02	1,11	3,77E-03	fatty acid desaturase 3
FAM135A	0,71	2,93E-02	0,38	3,87E-03	family with sequence similarity 135 member A
FCAR	1,84	1,67E-06	1,25	4,94E-02	Fc fragment of IgA receptor
FEZ2	0,82	7,71E-03	1,03	4,50E-09	fasciculation and elongation protein zeta 2
FGL2	2,78	4,90E-10	0,97	4,72E-02	fibrinogen-like protein 2
FNDC3A	0,81	4,08E-03	0,5	4,28E-03	fibronectin type III domain containing 3A
FNDC3B	0,73	5,71E-03	0,5	1,82E-03	fibronectin type III domain containing 3B
GAA	1,25	2,74E-03	0,78	7,33E-05	alpha glucosidase
GMFB	0,63	1,42E-02	0,81	3,16E-14	glia maturation factor beta
GNS	0,74	2,26E-03	0,54	1,24E-02	glucosamine (N-acetyl)-6-sulfatase
GOLIM4	0,7	1,49E-02	0,44	9,84E-06	golgi integral membrane protein 4
GPCPD1	0,91	4,92E-04	0,48	2,15E-02	glycerophosphocholine phosphodiesterase 1
GPR137B	1,15	9,22E-03	1,09	5,36E-04	G protein-coupled receptor 137B
H6PD	1,88	3,35E-06	1,64	1,97E-05	hexose-6-phosphate dehydrogenase/glucose 1-dehydrogenase
HEXB	0,65	1,85E-02	0,56	1,62E-02	hexosaminidase subunit beta
HLA-E	0,75	1,03E-02	0,29	3,38E-02	major histocompatibility complex, class I, E
HNMT	0,77	1,93E-02	0,48	1,63E-02	histamine N-methyltransferase
ICAM1	3,11	2,90E-02	2,83	4,23E-08	intercellular adhesion molecule 1
IDH1	0,57	9,15E-03	0,41	3,87E-03	isocitrate dehydrogenase (NADP(+)) 1
IFI16	2,33	4,81E-07	1,01	2,33E-02	interferon gamma inducible protein 16
IFNGR2	0,74	6,81E-03	0,58	2,55E-03	interferon gamma receptor 2
IGF2BP3	0,62	1,65E-02	0,41	4,13E-02	insulin like growth factor 2 mRNA binding protein 3
IL6ST	1,55	6,19E-07	0,74	6,07E-03	interleukin 6 cytokine family signal transduce
ITGAV	1,28	5,60E-05	0,75	2,78E-03	integrin subunit alpha V
ITGB2	0,73	1,56E-02	0,88	4,73E-02	integrin subunit beta 2
KIAA1191	0,58	3,45E-02	0,69	1,29E-05	KIAA1191
KRCC1	1,15	7,21E-04	0,51	8,44E-03	lysine rich coiled-coil 1

LAIR1	0,67	1,68E-02	0,7	6,76E-08	leukocyte associated immunoglobulin like receptor 1
LAPTM4A	1,01	3,15E-04	1,12	9,91E-20	lysosomal protein transmembrane 4 alpha
LCP2	0,77	3,03E-03	0,98	1,97E-05	lymphocyte cytosolic protein 2
LEPROT	1,07	9,59E-04	0,55	6,82E-03	leptin receptor overlapping transcript
LRP1	2,68	5,09E-05	1,64	2,62E-02	LDL receptor related protein 1
MAGEF1	1,01	3,90E-02	0,92	2,79E-02	MAGE family member F1
MANBA	1,07	3,46E-04	0,54	8,99E-04	mannosidase beta
MFSD6	1,44	8,60E-04	0,79	3,21E-02	major facilitator superfamily domain containing 6
MILR1	1,5	2,43E-02	1,08	5,84E-03	mast cell immunoglobulin like receptor 1
MR1	1,06	4,46E-04	0,73	2,81E-04	major histocompatibility complex, class I-related
MYD88	0,73	6,19E-03	0,56	3,87E-02	MYD88 innate immune signal transduction adaptor
MYO1C	0,87	3,02E-02	0,78	5,09E-04	myosin IC
NAGK	0,98	7,35E-03	1,14	5,41E-13	N-acetylglucosamine kinase
NCF1B	1,76	3,37E-03	1,58	2,13E-03	neutrophil cytosolic factor 1B pseudogene
NHLRC3	1	5,07E-04	0,71	2,33E-04	NHL repeat containing 3
NRP1	1,37	3,15E-05	0,69	1,87E-02	neuropilin 1
PAFAH2	0,83	3,32E-02	0,66	4,40E-02	platelet activating factor acetylhydrolase 2
PECAM1	1,64	1,28E-08	1,59	3,85E-11	platelet and endothelial cell adhesion molecule 1
PKN2	0,76	2,34E-02	0,44	4,27E-02	protein kinase N2
POLR3GL	0,73	1,80E-02	0,51	8,89E-03	RNA polymerase III subunit GL
PON2	0,6	1,88E-02	0,36	4,97E-02	paraoxonase 2
PPIC	1,14	1,64E-04	0,71	2,53E-05	peptidylprolyl isomerase C
PRKCA	0,98	1,20E-05	0,52	4,28E-03	protein kinase C alpha
PTTG1IP	0,53	4,49E-02	0,66	2,93E-04	PTTG1 interacting protein
RAB31	1,01	6,42E-04	0,65	3,06E-02	RAB31, member RAS oncogene family
RASSF2	0,65	5,03E-03	0,45	7,52E-03	Ras association domain family member 2
RDX	0,6	4,00E-02	0,53	3,10E-04	radixin
RHOU	1,01	4,92E-04	0,61	7,06E-03	ras homolog family member U
RRAGD	0,54	4,42E-02	0,63	1,57E-03	Ras related GTP binding D
SCARB2	0,62	1,48E-02	0,67	3,40E-02	scavenger receptor class B member 2
SEC23A	0,66	2,67E-02	0,44	2,21E-02	SEC23 homolog A, COPII coat complex component
SERINC3	0,55	2,94E-02	0,42	6,60E-05	serine incorporator 3

SETD7	0,91	1,62E-04	0,89	1,52E-13	SET domain containing 7, histone lysine methyltransferase
SGK3	1,04	6,88E-04	0,9	6,76E-08	serum/glucocorticoid regulated kinase family member 3
SGMS2	1,22	1,37E-02	0,95	4,71E-02	sphingomyelin synthase 2
SIGLEC14	1,35	9,71E-05	0,71	3,38E-02	sialic acid binding Ig like lectin 14
SIGLEC7	0,8	1,85E-02	0,63	1,02E-03	sialic acid binding Ig like lectin 7
SIGLEC9	1,64	3,80E-02	1,68	1,97E-03	sialic acid binding Ig like lectin 9
SKAP2	1,1	3,05E-05	0,57	4,18E-08	src kinase associated phosphoprotein 2
SLC2A6	1,32	3,41E-02	0,87	2,73E-03	solute carrier family 2 member 6
SLC30A1	0,63	3,53E-02	0,55	4,91E-02	solute carrier family 30 member 1
SMPD1	1,24	4,71E-03	1,04	5,92E-04	sphingomyelin phosphodiesterase 1
SNN	0,91	8,84E-03	0,57	2,45E-02	stannin
SNX30	0,83	3,34E-02	0,55	2,33E-03	sorting nexin family member 30
SORT1	1,62	4,92E-04	0,89	1,37E-02	sortilin 1
TGFBR2	1,38	2,07E-07	0,9	2,95E-06	transforming growth factor beta receptor 2
THBD	0,82	2,37E-03	0,95	1,42E-03	thrombomodulin
THEMIS2	0,99	9,04E-04	0,61	2,63E-02	thymocyte selection associated family member 2
TM6SF1	2,07	6,53E-03	1,99	9,11E-04	transmembrane 6 superfamily member 1
TMEM30A	0,55	3,90E-02	0,31	3,40E-02	transmembrane protein 30A
TNFRSF10B	0,92	6,67E-04	0,96	2,39E-06	TNF receptor superfamily member 10b
TWF1	0,78	2,34E-03	0,96	1,06E-07	twinfilin actin binding protein 1
UBA7	1,21	7,43E-03	0,61	2,88E-02	ubiquitin like modifier activating enzyme 7
UBASH3B	1,04	1,34E-04	0,43	3,28E-02	ubiquitin associated and SH3 domain containing B
USP3	0,62	2,70E-02	0,54	5,32E-03	ubiquitin specific peptidase 3
VSIR	0,86	6,62E-03	0,8	4,52E-03	V-set immunoregulatory receptor
WDR11	0,65	2,94E-02	0,27	4,38E-02	WD repeat domain 11
YPEL5	1,4	8,52E-04	0,92	1,20E-02	yippee like 5
ZNF367	0,63	1,37E-02	0,87	1,36E-08	zinc finger protein 367

Appendix Table S2. List of 74 genes commonly downregulated upon HIF2α silencing in HL60 and Kasumi1 cells.

Gene Symbol	HL60 Log_2FC	HL60 padj	Kasumi1 Log_2FC	Kasumi1 padj	Description
ABT1	-0,71	8,97E-03	-0,43	1,26E-02	activator of basal transcription 1
APBA2	-0,86	7,39E-03	-0,51	1,68E-02	amyloid beta precursor protein binding family A member 2
ASB6	-0,62	3,34E-02	-0,35	1,26E-02	ankyrin repeat and SOCS box containing 6
ASIC1	-0,74	4,16E-02	-0,53	4,91E-02	acid sensing ion channel subunit 1
BCL11A	-0,81	4,99E-02	-1,35	2,91E-03	BAF chromatin remodeling complex subunit BCL11A
CALR	-0,68	4,39E-03	-0,23	2,54E-02	calreticulin
CBX6	-1,04	5,47E-06	-0,88	3,89E-18	chromobox 6
CDK6	-0,69	2,70E-02	-0,66	1,78E-03	cyclin dependent kinase 6
CHERP	-0,58	2,93E-02	-0,36	8,19E-04	calcium homeostasis endoplasmic reticulum protein
CLUH	-0,65	6,86E-03	-0,28	9,22E-04	clustered mitochondria homolog
COL27A1	-1,05	1,67E-02	-1,37	1,91E-08	collagen type XXVII alpha 1 chain
CSK	-0,81	1,58E-03	-0,36	4,38E-02	C-terminal Src kinase
DDX54	-0,74	3,52E-03	-0,33	1,31E-02	DEAD-box helicase 54
DHX37	-0,89	3,47E-04	-0,25	2,44E-02	DEAD-box helicase 37
DLX2	-0,94	3,75E-02	-0,85	1,23E-03	distal-less homeobox 2
EIF1AX	-0,55	3,34E-02	-0,5	2,91E-05	eukaryotic translation initiation factor 1A X-linked
FAM216A	-0,66	1,34E-02	-0,6	6,15E-05	family with sequence similarity 216 member A
FBRSL1	-0,89	4,42E-04	-0,57	1,59E-04	fibrosin like 1
FHOD1	-0,55	3,93E-02	-0,36	2,78E-03	formin homology 2 domain containing 1
FLT3	-0,74	3,80E-02	-0,84	1,62E-03	fms related receptor tyrosine kinase 3
GEMIN4	-0,81	6,39E-04	-0,51	1,58E-04	gem nuclear organelle associated protein 4
HDLBP	-0,49	3,78E-02	-0,42	4,50E-09	high density lipoprotein binding protein
HIVEP2	-1,07	3,25E-02	-1	1,78E-02	HIVEP zinc finger 2
HSPB1	-0,93	1,33E-03	-0,32	4,43E-02	heat shock protein family B (small) member 1
IRF2BP1	-0,7	2,86E-02	-0,39	4,47E-02	interferon regulatory factor 2 binding protein 1
KIF21B	-0,66	1,12E-02	-0,49	3,51E-02	kinesin family member 21B

LGR4	-0,83	3,26E-02	-0,75	1,25E-04	leucine rich repeat containing G protein-coupled receptor 4
LMNB2	-0,81	1,22E-03	-0,28	2,91E-03	lamin B2
LPCAT1	-0,59	7,42E-03	-0,36	2,85E-04	lysophosphatidylcholine acyltransferase 1
LRP3	-1,02	1,08E-03	-0,71	4,51E-02	LDL receptor related protein 3
LRRC47	-0,57	3,56E-02	-0,35	2,32E-02	leucine rich repeat containing 47
LZTS2	-0,6	3,80E-02	-0,71	6,44E-06	leucine zipper tumor suppressor 2
MED24	-0,58	2,13E-02	-0,27	4,51E-02	mediator complex subunit 24
MPO	-1,69	1,85E-02	-1,47	9,60E-04	myeloperoxidase
MRM1	-0,79	1,57E-02	-0,51	7,38E-03	mitochondrial rRNA methyltransferase 1
MSRB3	-1,16	5,42E-07	-1,97	9,89E-23	methionine sulfoxide reductase B3
MYBBP1A	-0,66	4,46E-03	-0,24	3,76E-02	MYB binding protein 1a
NFKBIB	-0,71	1,33E-02	-0,36	4,81E-02	NFKB inhibitor beta
NUDT15	-0,75	1,80E-02	-0,82	5,17E-08	nudix hydrolase 15
P3H3	-0,85	2,91E-02	-0,68	3,67E-02	prolyl 3-hydroxylase 3
PPP2R2D	-0,64	2,76E-02	-0,55	2,85E-04	protein phosphatase 2 regulatory subunit Bdelta
PRR22	-1,51	3,67E-02	-0,76	2,02E-02	proline rich 22
PRRT4	-0,98	5,15E-04	-0,89	1,81E-02	proline rich transmembrane protein 4
PSMD3	-0,78	9,36E-04	-0,51	4,11E-07	proteasome 26S subunit, non-ATPase 3
PUS1	-0,74	6,10E-03	-0,38	5,35E-03	pseudouridine synthase 1
RAC3	-1,33	1,44E-04	-0,88	1,36E-07	Rac family small GTPase 3
RANGAP1	-0,65	9,09E-03	-0,2	3,40E-02	Ran GTPase activating protein 1
RASGRP2	-0,67	2,57E-02	-0,52	9,56E-03	RAS guanyl releasing protein 2
REXO4	-0,69	4,02E-03	-0,58	2,70E-08	REX4 homolog, 3'-5' exonuclease
RGS18	-0,76	1,01E-02	-0,79	1,93E-02	regulator of G protein signaling 18
RHOT2	-0,76	3,59E-03	-0,22	4,05E-02	ras homolog family member T2
RNF216P1	-0,86	2,09E-03	-0,74	3,44E-03	ring finger protein 216 pseudogene 1
RP11-498C9.15	-0,89	2,02E-03	-1,18	6,39E-03	pre-mRNA processing factor 31
RPIA	-0,51	3,85E-02	-0,26	4,16E-02	ribose 5-phosphate isomerase A
RUNX2	-0,79	4,90E-01	-1,25	3,52E-06	Runt-Related Transcription Factor 2
SART1	-0,63	2,06E-02	-0,33	3,09E-02	spliceosome associated factor 1, recruiter of U4/U6.U5 tri-snRNP
SERPINB10	-1,14	4,76E-05	-1,66	7,97E-05	serpin family B member 10
SERPINB2	-1,01	5,09E-06	-2,51	1,51E-08	serpin family B member 2

SIRT7	-0,57	3,15E-02	-0,41	6,07E-03	sirtuin 7
SLC7A5	-1,29	1,94E-08	-0,52	2,63E-10	solute carrier family 7 member 5
SMPD4	-0,7	6,42E-03	-0,27	4,65E-02	sphingomyelin phosphodiesterase 4
TBC1D2B	-0,54	4,50E-02	-0,55	2,80E-05	TBC1 domain family member 2B
TCOF1	-0,73	2,43E-03	-0,25	1,58E-02	treacle ribosome biogenesis factor 1
TELO2	-1,07	1,56E-04	-0,4	8,69E-06	telomere maintenance 2
TMTC2	-0,73	3,98E-02	-0,75	1,26E-03	transmembrane O-mannosyltransferase targeting cadherins 2
TNKS1BP1	-0,75	2,89E-02	-0,68	2,67E-04	tankyrase 1 binding protein 1
TONSL	-0,93	1,79E-03	-0,4	4,01E-03	tonsoku like, DNA repair protein
TRIM28	-0,72	2,43E-03	-0,27	5,65E-03	tripartite motif containing 28
TUSC2	-0,54	4,74E-02	-0,46	1,17E-02	tumor suppressor 2, mitochondrial calcium regulator
UHRF1	-0,86	4,77E-03	-0,64	3,04E-12	ubiquitin like with PHD and ring finger domains 1
VIT	-0,65	3,32E-02	-0,77	2,80E-02	vitrin
WDR18	-1,08	4,11E-04	-0,29	1,57E-02	WD repeat domain 18
WDR4	-0,83	5,55E-03	-0,52	2,54E-02	WD repeat domain 4
ZFP37	-1,49	3,56E-02	-1,25	2,96E-02	ZFP37 zinc finger protein

Appendix Table S3. Probes for qPCR.

Gene	Assay ID
EPAS1	Hs01026142_m1
HIF1A	Hs00936371_m1
FLT3	Hs00174690_m1
CDK6	Hs01026371_m1
TRIM28	Hs00232212_m1
BCL11A	Hs01093197_m1
RUNX2	Hs01047973_m1
UHRF1	Hs01086727_m1
PGK1	Hs00943178_g1
PDK1	Hs01561847_m1
ITGAM	Hs00167304_m1
ITGB2	Hs00164957_m1
CD53	Hs00174065_m1
IFI16	Hs00986757_m1
18s	Hs03003631_g1

Appendix Table S4. Sequences of primers for ChIP experiments.

Primers	Sequence (5'-3')
BCL11A forward	AAAGGTGCGTGCTGTCTCAA
BCL11A reverse	AACCCTCATCCCATCTGGAAC
HIF2α forward	CCCCAACCCTTTCTGTGTAC
HIF2α reverse	GGACCCATGAGTTTCACAGG
RARβ forward	TCCTGGGAGTTGGTGATGTCAG
RARβ reverse	AAACCCTGCTCGGATCGCTC
RUNX2 forward	TCTTTATTGTAAAGGCCGCCA
RUNX2 reverse	CATGAAAGTGTTAGTCTGGGCA
SLC7A5 forward	TGGCATGACAGTAGGGTTGT
SLC7A5 reverse	GAATTTCCAGTACACGCTGCC
