# Supplementary figures and images for: The transcription factor HIF2α partakes in the differentiation block of acute myeloid leukemia
Source: EMBO Mol Med. 2023 Oct 9;15(11):e17810. doi: 10.15252/emmm.202317810 (PMC10630882; doi:10.15252/emmm.202317810)

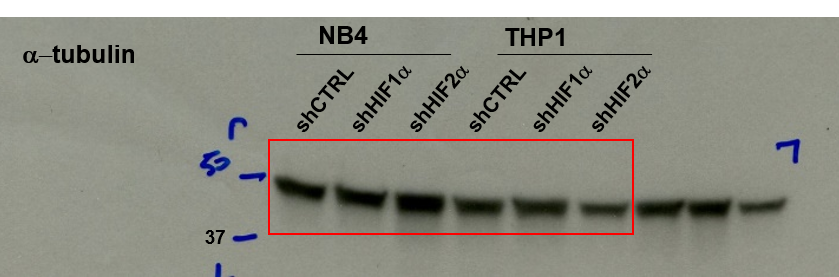

Supplement: Supplementary file 4 — Source Data for Figure 1 [file EMMM-15-e17810-s010.zip › Figure 1/1A/NB4_THP1_a-tubulin.tif]

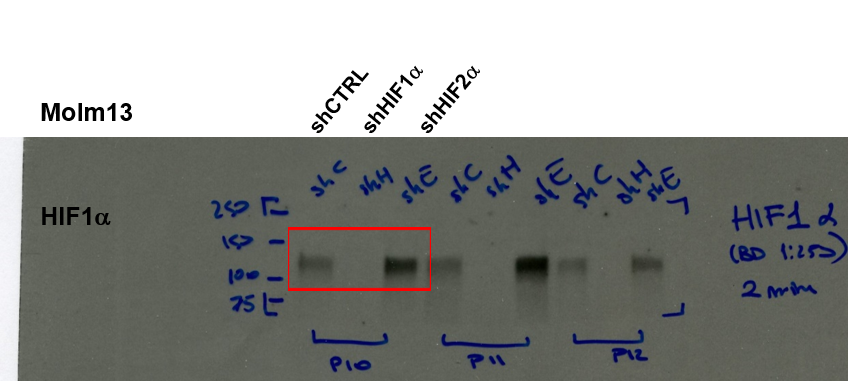

Supplement: Supplementary file 4 — Source Data for Figure 1 [file EMMM-15-e17810-s010.zip › Figure 1/1A/Molm13_HIF1a.tif]

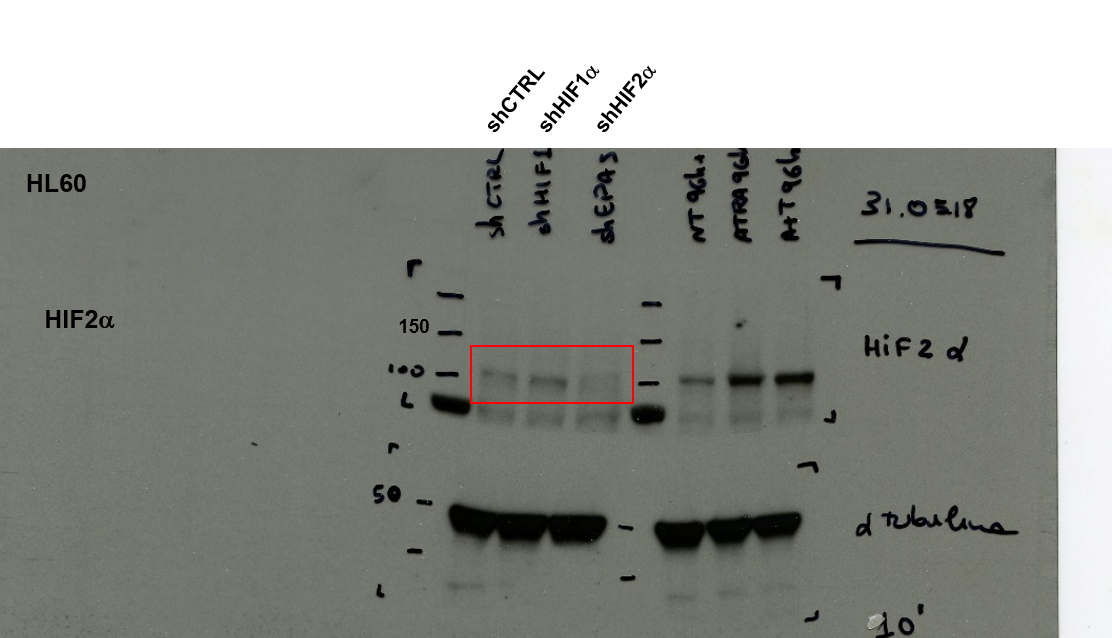

Supplement: Supplementary file 4 — Source Data for Figure 1 [file EMMM-15-e17810-s010.zip › Figure 1/1A/HL60_HIF2a.tif]

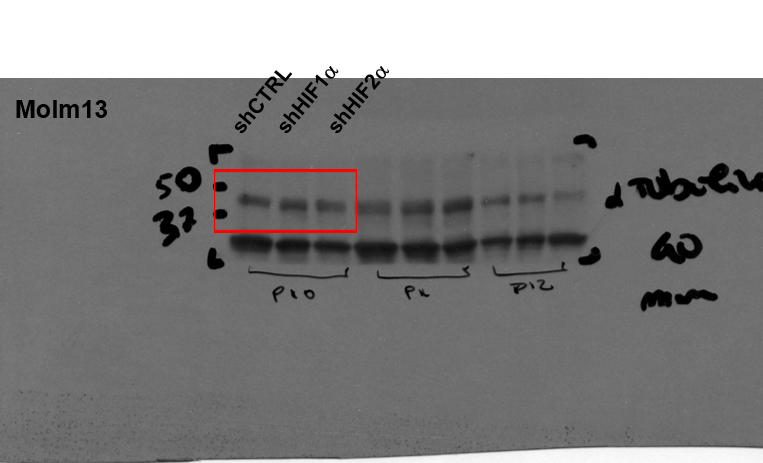

Supplement: Supplementary file 4 — Source Data for Figure 1 [file EMMM-15-e17810-s010.zip › Figure 1/1A/Molm13_a-tubulin.tif]

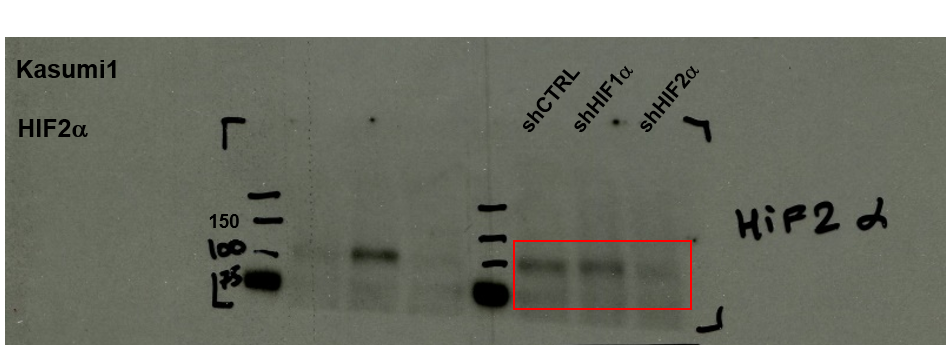

Supplement: Supplementary file 4 — Source Data for Figure 1 [file EMMM-15-e17810-s010.zip › Figure 1/1A/Kasumi1_HIF2a.tif]

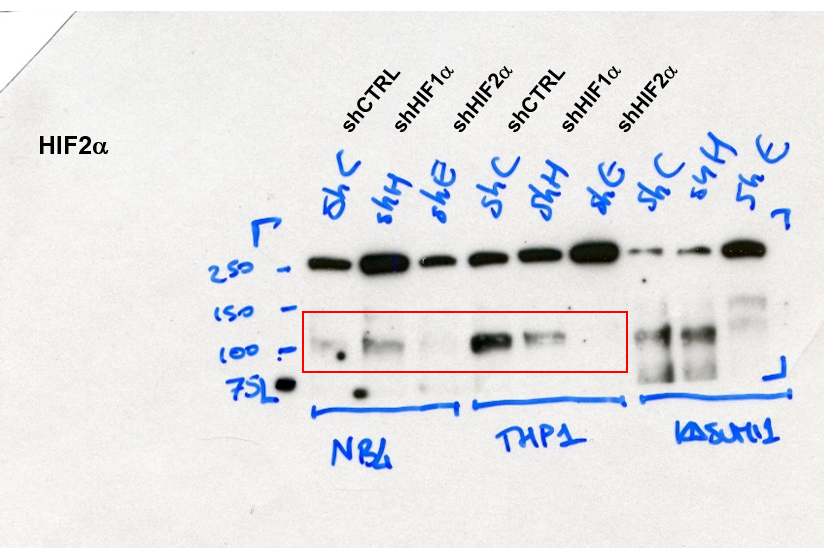

Supplement: Supplementary file 4 — Source Data for Figure 1 [file EMMM-15-e17810-s010.zip › Figure 1/1A/NB4_THP1_HIF2a.tif]

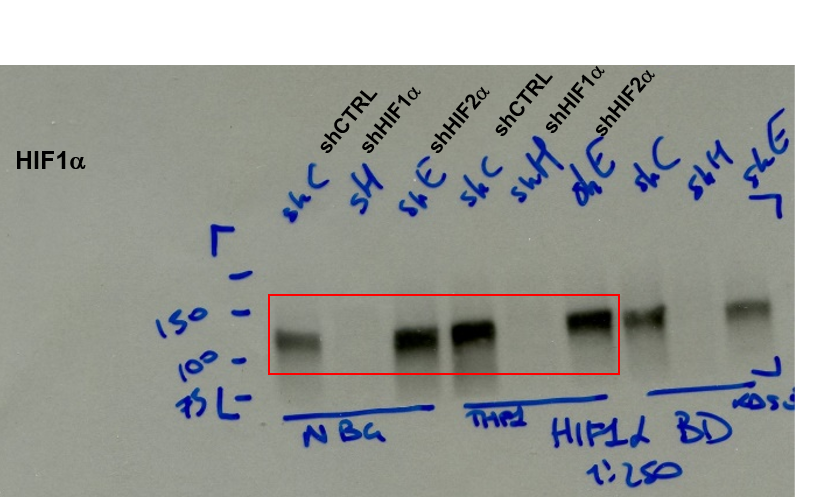

Supplement: Supplementary file 4 — Source Data for Figure 1 [file EMMM-15-e17810-s010.zip › Figure 1/1A/NB4_THP1_HIF1a.tif]

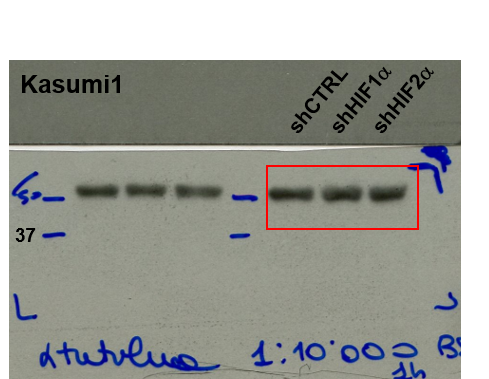

Supplement: Supplementary file 4 — Source Data for Figure 1 [file EMMM-15-e17810-s010.zip › Figure 1/1A/Kasumi1_a-tubulin.tif]

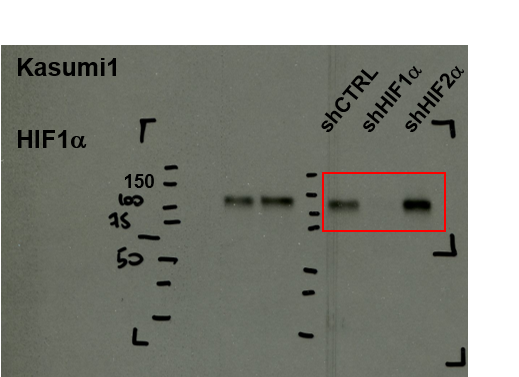

Supplement: Supplementary file 4 — Source Data for Figure 1 [file EMMM-15-e17810-s010.zip › Figure 1/1A/Kasumi1_HIF1a.tif]

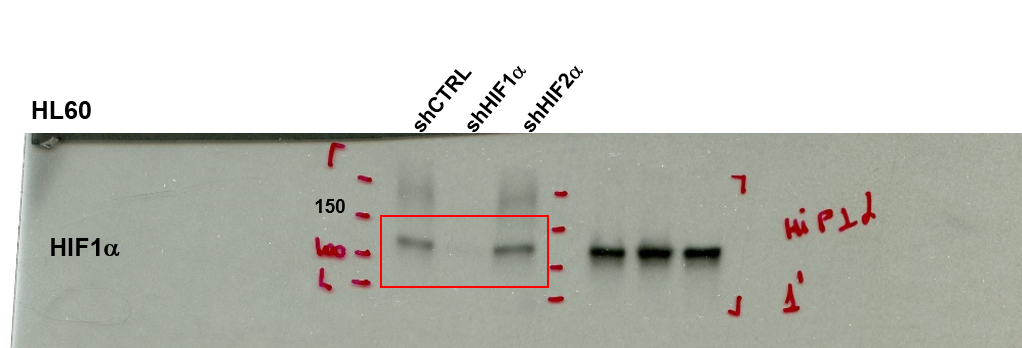

Supplement: Supplementary file 4 — Source Data for Figure 1 [file EMMM-15-e17810-s010.zip › Figure 1/1A/HL60_HIF1a.tif]

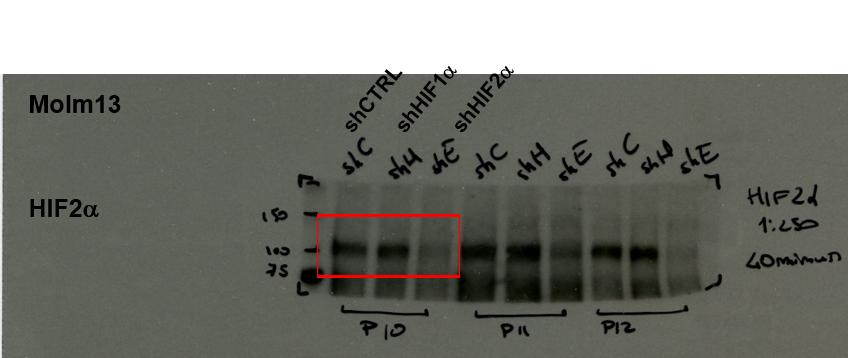

Supplement: Supplementary file 4 — Source Data for Figure 1 [file EMMM-15-e17810-s010.zip › Figure 1/1A/Molm13_HIF2a.tif]

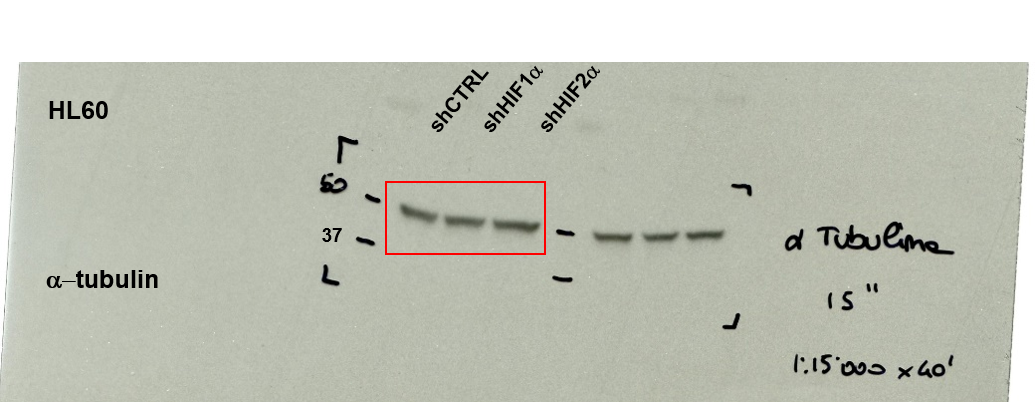

Supplement: Supplementary file 4 — Source Data for Figure 1 [file EMMM-15-e17810-s010.zip › Figure 1/1A/HL60_a-tubulin.tif]

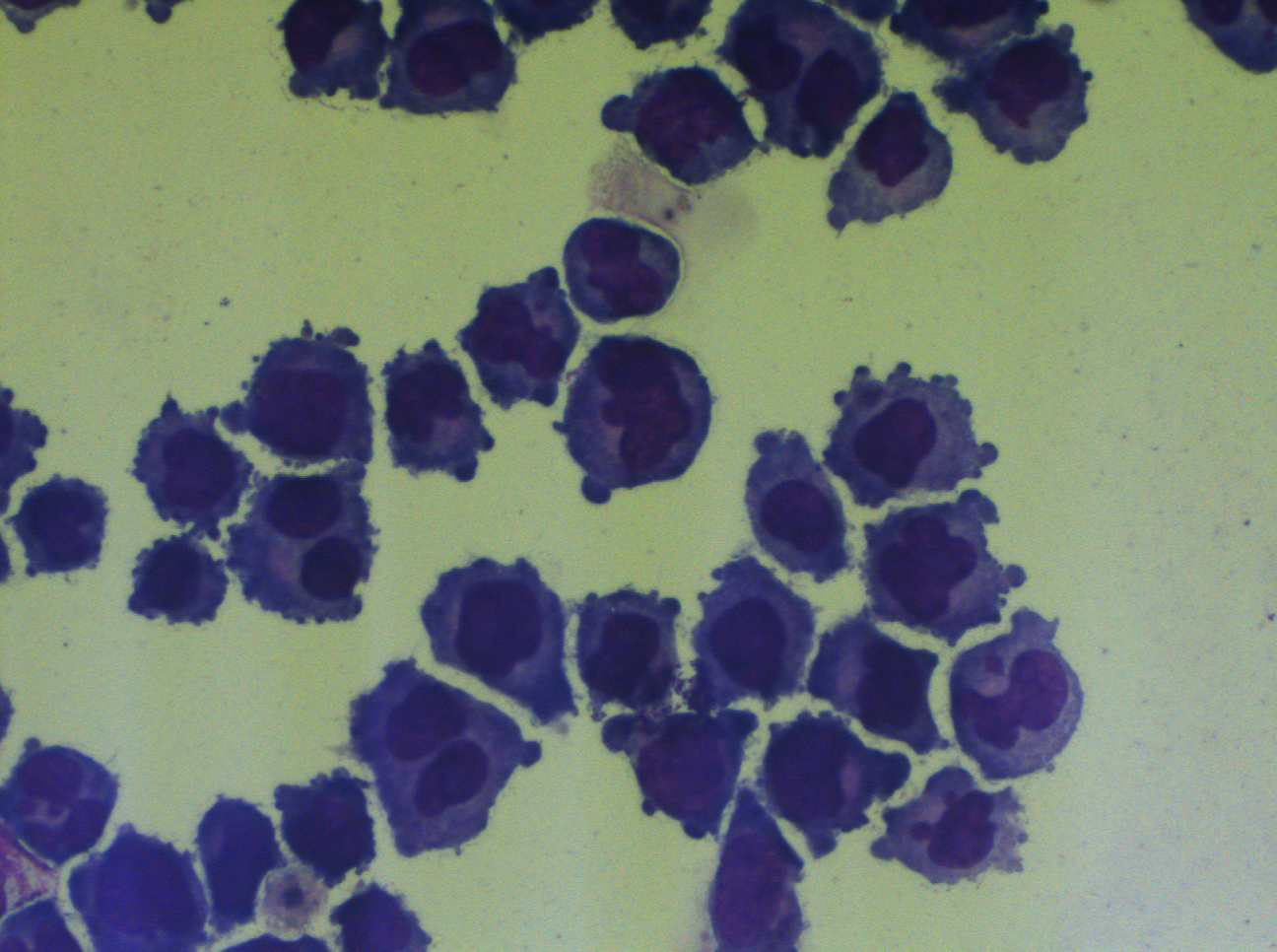

Supplement: Supplementary file 4 — Source Data for Figure 1 [file EMMM-15-e17810-s010.zip › Figure 1/1D/Microscopic Images/Kasumi1 shHIF2a.tif]

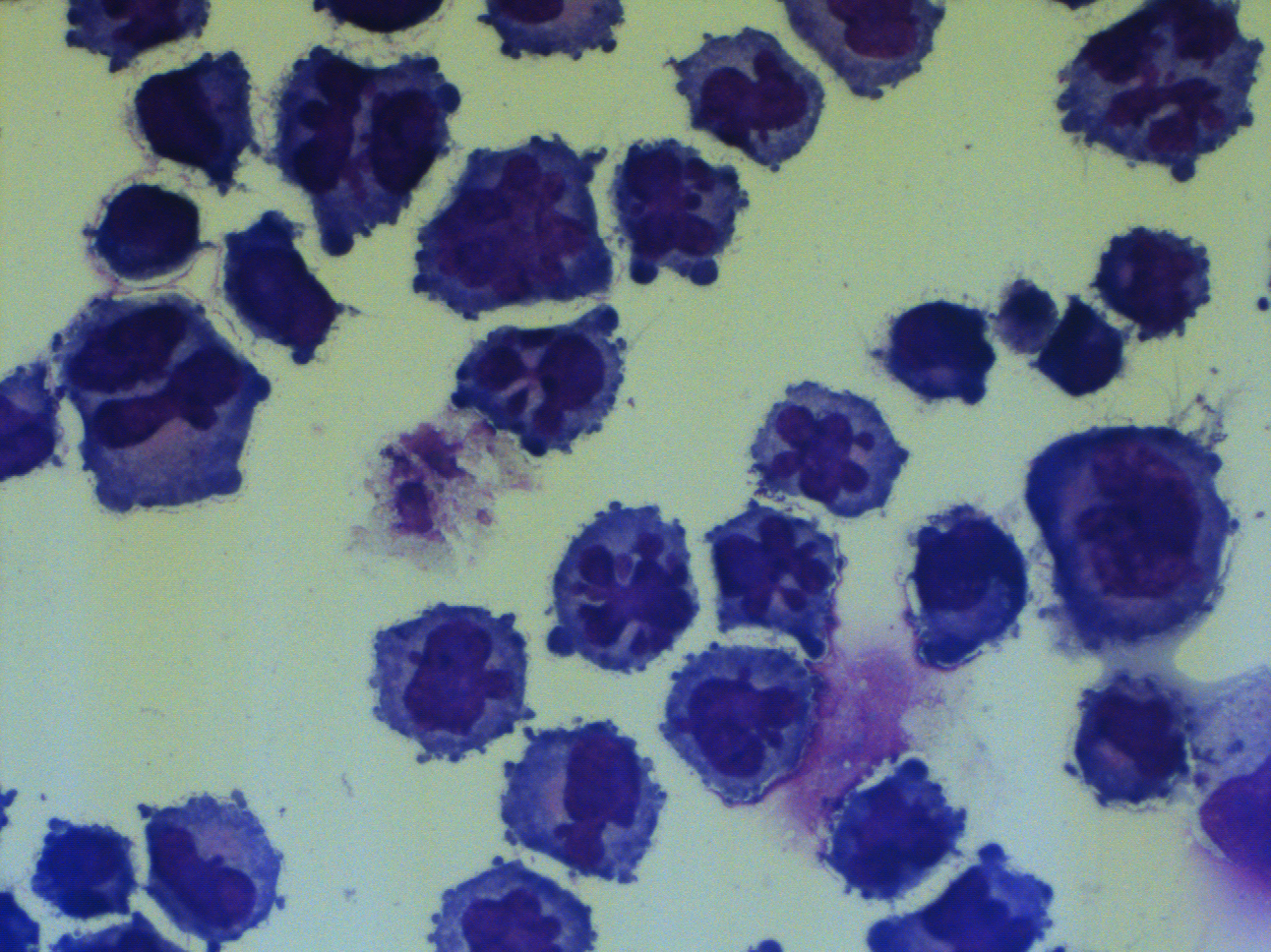

Supplement: Supplementary file 4 — Source Data for Figure 1 [file EMMM-15-e17810-s010.zip › Figure 1/1D/Microscopic Images/NB4 shHIF2a.tif]

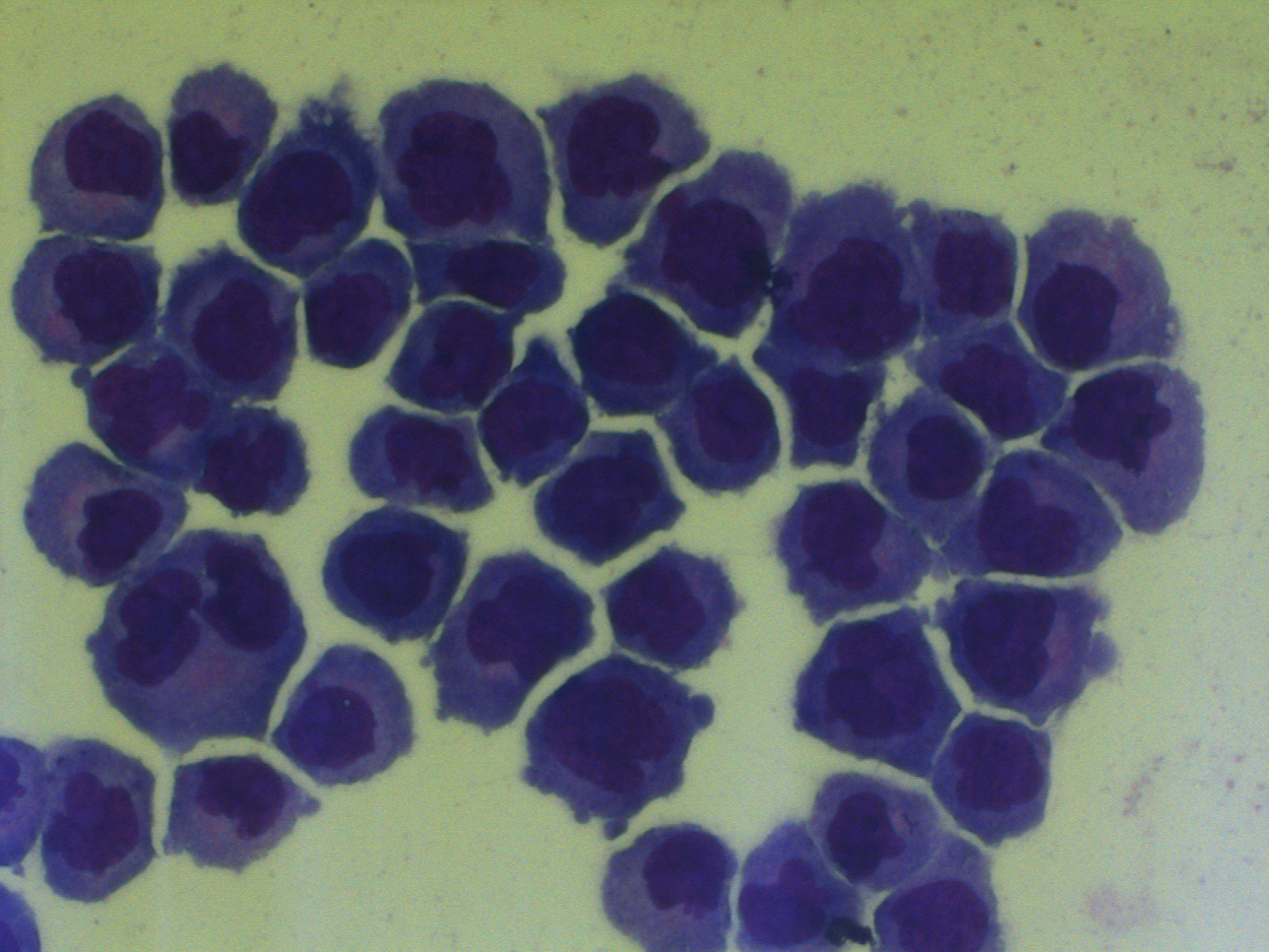

Supplement: Supplementary file 4 — Source Data for Figure 1 [file EMMM-15-e17810-s010.zip › Figure 1/1D/Microscopic Images/Kasumi1 shCtrl.tif]

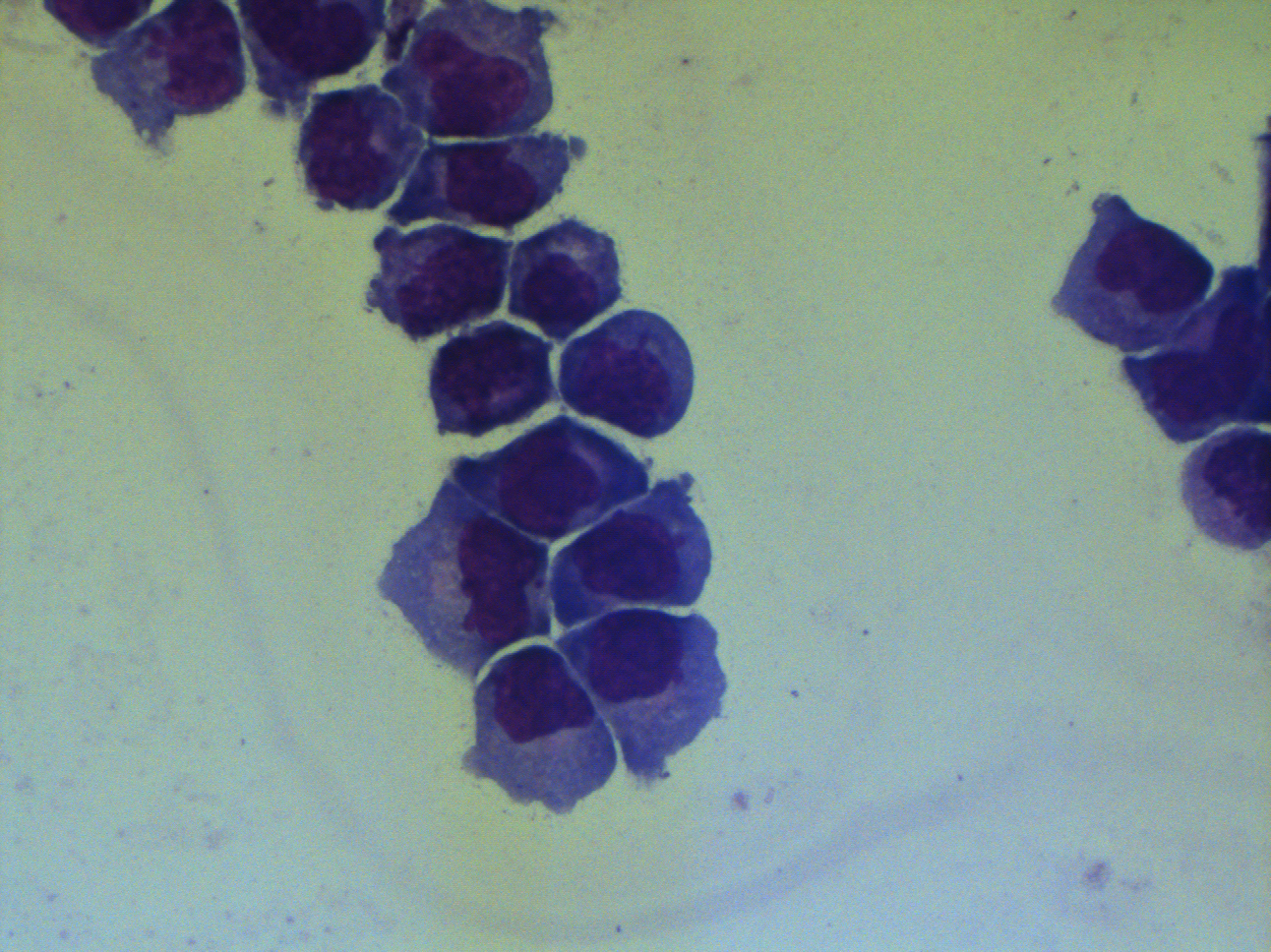

Supplement: Supplementary file 4 — Source Data for Figure 1 [file EMMM-15-e17810-s010.zip › Figure 1/1D/Microscopic Images/NB4 shCtrl.tif]

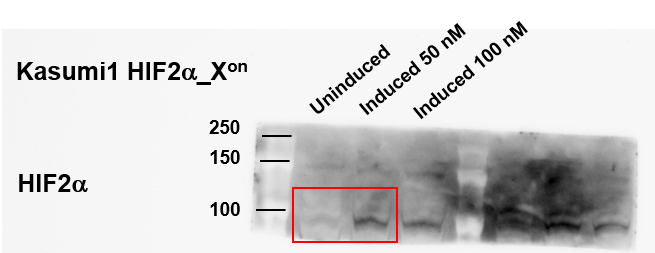

Supplement: Supplementary file 5 — Source Data for Figure 2 [file EMMM-15-e17810-s007.zip › Figure 2/2I/Kasumi1 HIF2a_Xon_HIF2a.tif]

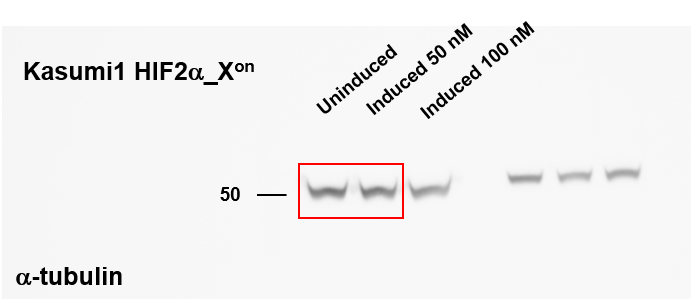

Supplement: Supplementary file 5 — Source Data for Figure 2 [file EMMM-15-e17810-s007.zip › Figure 2/2I/Kasumi1 HIF2a_Xon_a-tubulin.tif]

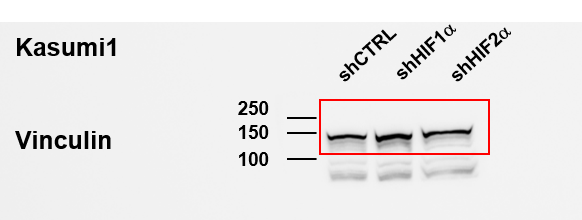

Supplement: Supplementary file 5 — Source Data for Figure 2 [file EMMM-15-e17810-s007.zip › Figure 2/2E/Kasumi1_Vinculin.tif]

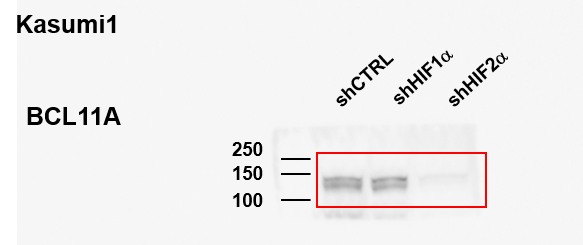

Supplement: Supplementary file 5 — Source Data for Figure 2 [file EMMM-15-e17810-s007.zip › Figure 2/2E/Kasumi1_BCL11A.tif]

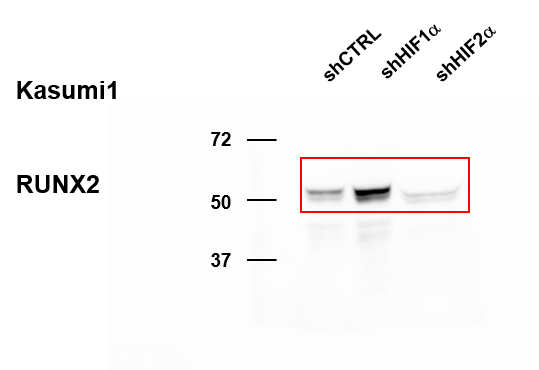

Supplement: Supplementary file 5 — Source Data for Figure 2 [file EMMM-15-e17810-s007.zip › Figure 2/2E/Kasumi1_RUNX2.tif]

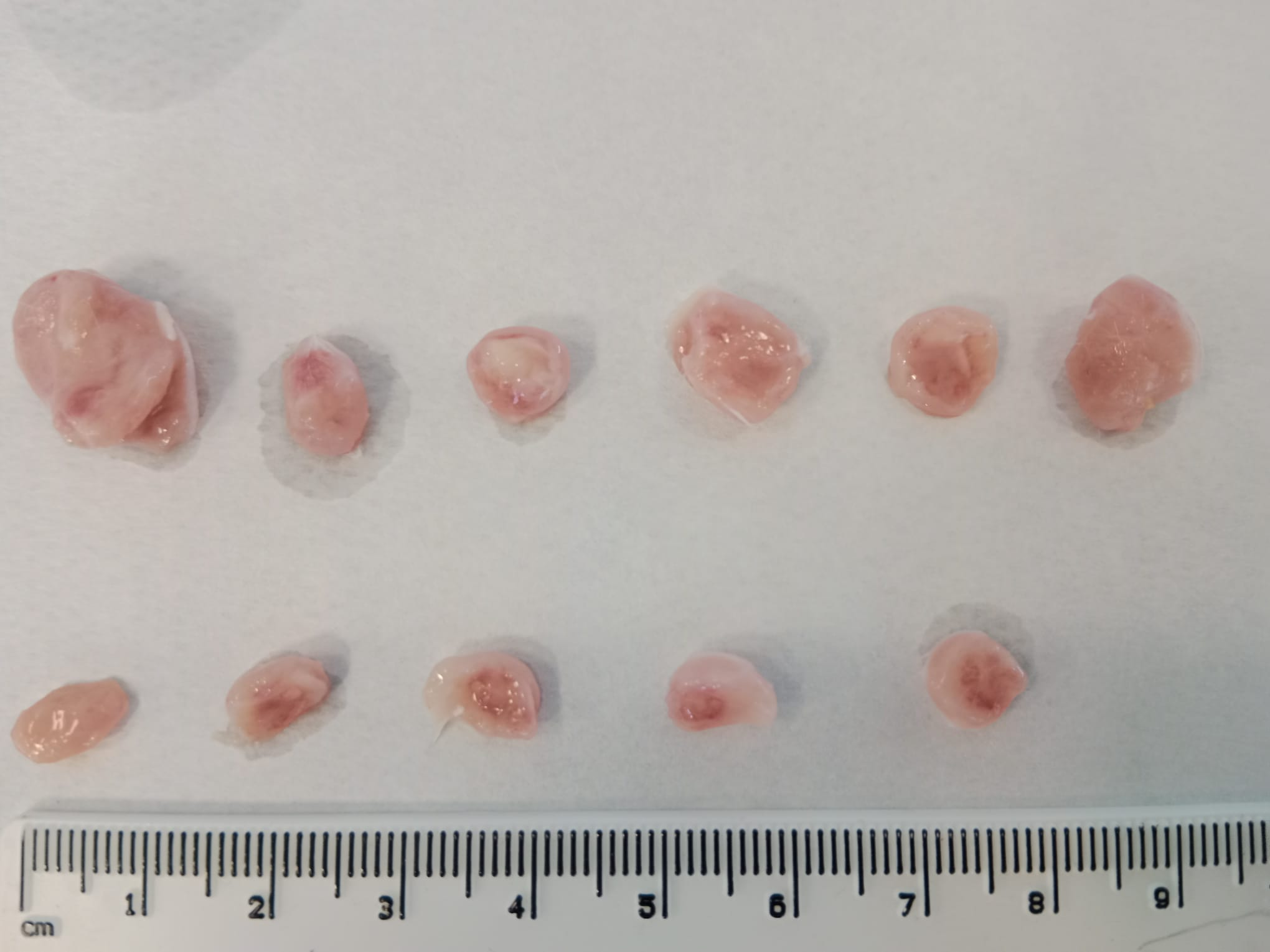

Supplement: Supplementary file 6 — Source Data for Figure 3 [file EMMM-15-e17810-s004.zip › Figure 3/3E/Kasumi1 Tumors day20.tiff]

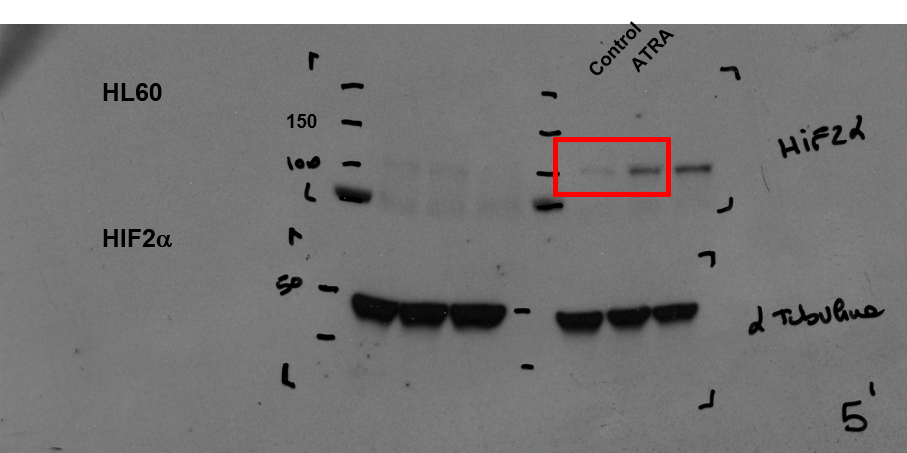

Supplement: Supplementary file 9 — Source Data for Figure 6 [file EMMM-15-e17810-s003.zip › Figure 6/6B/HL60_HIF2a.tif]

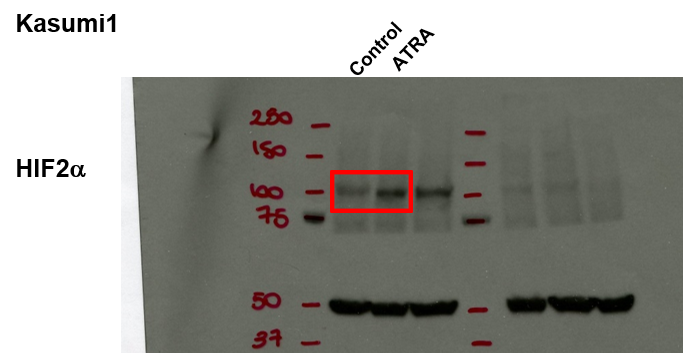

Supplement: Supplementary file 9 — Source Data for Figure 6 [file EMMM-15-e17810-s003.zip › Figure 6/6B/Kasumi1_HIF2a.tif]

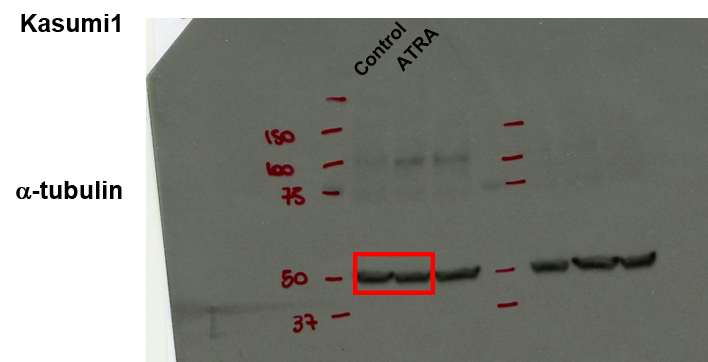

Supplement: Supplementary file 9 — Source Data for Figure 6 [file EMMM-15-e17810-s003.zip › Figure 6/6B/Kasumi1_a-tubulin.tif]

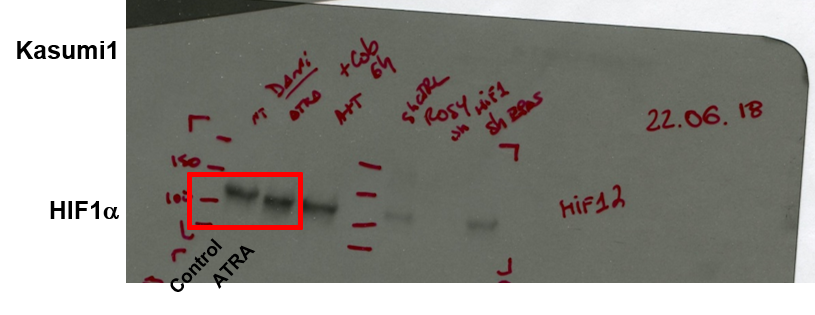

Supplement: Supplementary file 9 — Source Data for Figure 6 [file EMMM-15-e17810-s003.zip › Figure 6/6B/Kasumi1_HIF1a.tif]

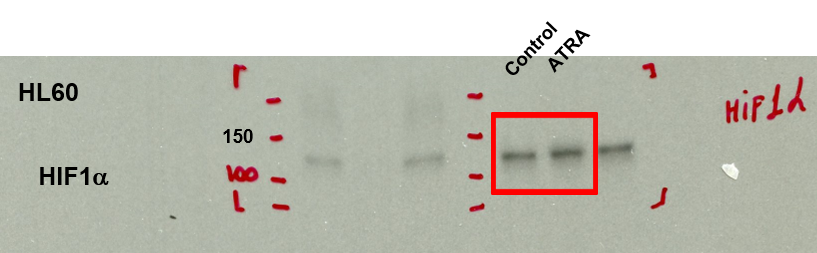

Supplement: Supplementary file 9 — Source Data for Figure 6 [file EMMM-15-e17810-s003.zip › Figure 6/6B/HL60_HIF1a.tif]

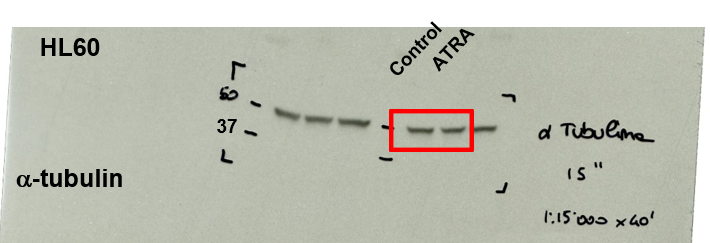

Supplement: Supplementary file 9 — Source Data for Figure 6 [file EMMM-15-e17810-s003.zip › Figure 6/6B/HL60_a-tubulin.tif]
