# Supplementary material for: The transcription factor HIF2α partakes in the differentiation block of acute myeloid leukemia
Source: EMBO Mol Med. 2023 Oct 9;15(11):e17810. doi: 10.15252/emmm.202317810 (PMC10630882; doi:10.15252/emmm.202317810)
Supplement: Supplementary file 10 [file EMMM-15-e17810-s002.pdf]

EMBO Molecular Medicine

The transcription factor HIF2a partakes in the differentiation block of acute myeloid leukemia

Daniela Magliulo, Matilde Simoni, Carolina Caserta, Cristina Fracassi, Serena Belluschi, Kety Giannetti, Raffaella Pini, Ettore Zapparoli, Stefano Beretta, Martina Ugge', Eleonora Draghi, Federico Rossari, Nadia Coltella, Cristina Tresoldi, Marco Morelli, Raffaella Di Micco, Bernhard Gentner, Luca Vago, and Rosa Bernardi
DOI: 10.15252/emmm.202317810

Corresponding author: Rosa Bernardi (bernardi.rosa@hsr.it)

Review Timeline:

Submission Date:	5th Apr 23
Editorial Decision:	8th May 23
Revision Received:	25th Aug 23
Editorial Decision:	11th Sep 23
Revision Received:	19th Sep 23
Accepted:	21st Sep 23

Editor: Lise Roth

Transaction Report:

(Note: With the exception of the correction of typographical or spelling errors that could be a source of ambiguity, letters and reports are not edited. Depending on transfer agreements, referee reports obtained elsewhere may or may not be included in this compilation. Referee reports are anonymous unless the Referee chooses to sign their reports.)

8th May 2023

Dear Dr. Bernardi,

Thank you for the submission of your manuscript to EMBO Molecular Medicine, and please accept my apologies for the delay in getting back to you, as we were expecting an additional report. Unfortunately reviewer #2 will not be able to provide a report in a timely fashion, and we decided to make a decision with the 2 reports at hand in order not to delay the process further.
As you will see below, while reviewer #3 is overall supportive of publication (pending validation via genetic approaches), reviewer #1 raises substantial concerns on your work, which unfortunately preclude its publication in EMBO Molecular Medicine in its current form.

If you feel you can satisfactorily address the points raised by the referees, you may wish to submit a revised version of your manuscript. Please attach a covering letter giving details of the way in which you have handled each of the points raised by the referees. A revised manuscript will once again be subject to review and we cannot guarantee at this stage that the eventual outcome will be favorable. EMBO Molecular Medicine encourages a single round of revision only and therefore, acceptance or rejection of the manuscript will depend on the completeness of your responses included in the next, final version of the manuscript. For this reason, and to save you from any frustrations in the end, I would strongly advise against returning an incomplete revision.

If you would like to discuss further the points raised by the referees, I am available to do so via email or video. Let me know if you are interested in this option.

Revised manuscripts should be submitted within three months of a request for revision; they will otherwise be treated as new submissions, except under exceptional circumstances in which a short extension is obtained from the editor.

When submitting your revised manuscript, please carefully review the instructions that follow below. We perform an initial quality control of all revised manuscripts before re-review; failure to include requested items will delay the evaluation of your revision.

We require:

1) A .docx formatted version of the manuscript text (including legends for main figures, EV figures and tables). Please make sure that the changes are highlighted to be clearly visible.

2) Individual production quality figure files as .eps, .tif, .jpg (one file per figure). For guidance, download the 'Figure Guide PDF' (https://www.embopress.org/page/journal/17574684/authorguide#figureformat).

3) At EMBO Press we ask authors to provide source data for the main figures. Our source data coordinator will contact you to discuss which figure panels we would need source data for and will also provide you with helpful tips on how to upload and organize the files.

4) A .docx formatted letter INCLUDING the reviewers' reports and your detailed point-by-point responses to their comments. As part of the EMBO Press transparent editorial process, the point-by-point response is part of the Review Process File (RPF), which will be published alongside your paper.

5) A complete author checklist, which you can download from our author guidelines (https://www.embopress.org/page/journal/17574684/authorguide#submissionofrevisions). Please insert information in the checklist that is also reflected in the manuscript. The completed author checklist will also be part of the RPF.

6) Please note that all corresponding authors are required to supply an ORCID ID for their name upon submission of a revised manuscript.

7) It is mandatory to include a 'Data Availability' section after the Materials and Methods. Before submitting your revision, primary datasets produced in this study need to be deposited in an appropriate public database, and the accession numbers and database listed under 'Data Availability'. Please remember to provide a reviewer password if the datasets are not yet public (see https://www.embopress.org/page/journal/17574684/authorguide#dataavailability).
In case you have no data that requires deposition in a public database, please state so in this section (This study includes no data deposited in external repositories). Note that the Data Availability Section is restricted to new primary data that are part of this study.

8) For data quantification: please specify the name of the statistical test used to generate error bars and P values, the number (n) of independent experiments (specify technical or biological replicates) underlying each data point and the test used to

calculate p-values in each figure legend. The figure legends should contain a basic description of n, P and the test applied. Graphs must include a description of the bars and the error bars (s.d., s.e.m.). Please provide exact p values.

9) Our journal encourages inclusion of *data citations in the reference list* to directly cite datasets that were re-used and obtained from public databases. Data citations in the article text are distinct from normal bibliographical citations and should directly link to the database records from which the data can be accessed. In the main text, data citations are formatted as follows: "Data ref: Smith et al, 2001" or "Data ref: NCBI Sequence Read Archive PRJNA342805, 2017". In the Reference list, data citations must be labeled with "[DATASET]". A data reference must provide the database name, accession number/identifiers and a resolvable link to the landing page from which the data can be accessed at the end of the reference. Further instructions are available at .

10) We replaced Supplementary Information with Expanded View (EV) Figures and Tables that are collapsible/expandable online. A maximum of 5 EV Figures can be typeset. EV Figures should be cited as 'Figure EV1, Figure EV2" etc... in the text and their respective legends should be included in the main text after the legends of regular figures.
- For the figures that you do NOT wish to display as Expanded View figures, they should be bundled together with their legends in a single PDF file called *Appendix*, which should start with a short Table of Content. Appendix figures should be referred to in the main text as: "Appendix Figure S1, Appendix Figure S2" etc.
- Additional Tables/Datasets should be labeled and referred to as Table EV1, Dataset EV1, etc. Legends have to be provided in a separate tab in case of .xls files. Alternatively, the legend can be supplied as a separate text file (README) and zipped together with the Table/Dataset file.
See detailed instructions here:

11) The paper explained: EMBO Molecular Medicine articles are accompanied by a summary of the articles to emphasize the major findings in the paper and their medical implications for the non-specialist reader. Please provide a draft summary of your article highlighting
- the medical issue you are addressing,
- the results obtained and
- their clinical impact.
This may be edited to ensure that readers understand the significance and context of the research. Please refer to any of our published articles for an example.

12) For more information: There is space at the end of each article to list relevant web links for further consultation by our readers. Could you identify some relevant ones and provide such information as well? Some examples are patient associations, relevant databases, OMIM/proteins/genes links, author's websites, etc...

13) Author contributions: CRediT has replaced the traditional author contributions section because it offers a systematic machine readable author contributions format that allows for more effective research assessment. Please remove the Authors Contributions from the manuscript and use the free text boxes beneath each contributing author's name in our system to add specific details on the author's contribution. More information is available in our guide to authors.

14) Disclosure statement and competing interests: We updated our journal's competing interests policy in January 2022 and request authors to consider both actual and perceived competing interests. Please review the policy https://www.embopress.org/competing-interests and update your competing interests if necessary.

15) Every published paper now includes a 'Synopsis' to further enhance discoverability. Synopses are displayed on the journal webpage and are freely accessible to all readers. They include a short stand first (maximum of 300 characters, including space) as well as 2-5 one-sentences bullet points that summarizes the paper. Please write the bullet points to summarize the key NEW findings. They should be designed to be complementary to the abstract - i.e. not repeat the same text. We encourage inclusion of key acronyms and quantitative information (maximum of 30 words / bullet point). Please use the passive voice. Please attach these in a separate file or send them by email, we will incorporate them accordingly.

Please also suggest a striking image or visual abstract to illustrate your article as a PNG file 550 px wide x 300-600 px high.

16) As part of the EMBO Publications transparent editorial process initiative (see our Editorial at http://embomolmed.embopress.org/content/2/9/329), EMBO Molecular Medicine will publish online a Review Process File (RPF) to accompany accepted manuscripts.
In the event of acceptance, this file will be published in conjunction with your paper and will include the anonymous referee reports, your point-by-point response and all pertinent correspondence relating to the manuscript. Let us know whether you agree with the publication of the RPF and as here, if you want to remove or not any figures from it prior to publication.
Please note that the Authors checklist will be published at the end of the RPF.

EMBO Molecular Medicine has a "scooping protection" policy, whereby similar findings that are published by others during review or revision are not a criterion for rejection. Should you decide to submit a revised version, I do ask that you get in touch

after three months if you have not completed it, to update us on the status.

I look forward to receiving your revised manuscript.

Yours sincerely,

Lise Roth

Lise Roth, PhD
Senior Editor
EMBO Molecular Medicine

***** Reviewer's comments *****

Referee #1 (Remarks for Author):

In this manuscript, Magliulo et al studied the role of the HIF2a transcription factor in AML differentiation. In particular, they provide evidence that decreased expression of HIF2A induces the differentiation and death of various leukemic cell lines. This correlates with the activation of genes linked to differentitation. They then use inhibitors of HIF2. One of them (EZN2208) is a topoisomerase inhibitor, which induces a decrease in HIF proteins expression. It has antileukemic action and increases differentiation in PDX models. The other, PT2385 is more specific and induces differentation in vitro. In addition, it favors ATRA-induced differentiation of non-APL AML cell lines.
Restoration of leukemic cells differentiation is a sound approach and many AML therapies induce their differentiation. This is of course the case of ATRA but also of Azacitidine, FLT3 or IDH inhibitors. The link between HIF2 and AML differentiation is therefore interesting. However, the conclusions are not well supported by the data and the manuscript lacks convincing evidence of the pro-differentiation/anti-leukemic potential of HIF2 targeting in relevant preclinical models.

Major:

Throughout the manuscript, the effect of HIF2a targeting (shRNA or inhibitors) on differentiation is monitored by the % of CD11b expressing cells. The cell lines used are supposed to be monoclonal and expected to behave homogeneously. It is thus expected that all cells acquire CD11b expression. The authors should also provide representative cytometry profiles as well as histograms representing the average results of Median Fluorescence Intensity for CD11b. This is all the more important that the % of differentiated cells upon HIF2 KD is modest in the non-APL cell lines.

In addition, the effect on differentiation was measured after 48hrs, which is very early as the differentiation process generally takes up to a week in these cell lines. Kinetics experiments should be performed. The authors should check other markers such as CD14 and CD15, at least on the cell lines.

ATAC-seq and H3K27me3 Chip seq were performed upon HIF2a KD. However, the results of these experiments are not well analyzed. The authors only present the analysis of the changes in chromatin accessibility and H3K27me3 at 3 loci: ITGB2, CD53 and IFI16. This is odd as they even didn't check if HIF2 is actually binding to these genes. What happens on the rest of the genome and at genes up- or down regulated by HIF2? The authors should have performed CHIP-Seq for HIF2 to identify genes, which are directly regulated by this transcription factor and analyze chromatin accessibility at these loci.

As the authors didn't see an effect of HIF KD on differentiation in PDX models, they resorted to EZN-2208. They write that it « acts as a potent HIFa inhibitor when used at non-cytotoxic concentration ». Then they write «this suggests that acute pharmacological inhibition of HIF2a... » This is clearly misleading as it suggests that this drug directly targets HIF protein. This is not the case. EZN-2208 is a toposisomerase inhibitor. Although it clearly decreases HIF expression, it has many other effects and the authors can clearly not conclude that inhibition of HIF2a exerts a pro-differentiation role.

Instead, the authors should perform in vivo experiments using AML cell lines knocked-down or knocked-out by CRIPSR for HIF2a and monitor tumor progression and differentiation. Similar to in vitro experiments, more differentiation markers should be used (at least CD14 and CD15). At least, they should use the HIF specific inhibitor PT2385 in the PDX model in vivo and not only ex vivo. The authors state that they only performed in vitro experiments with PT2385 because the molecule for in vivo studies was not available. However, in the paper they cite (Wallace et al, 2016), in vivo experiments were performed using PT2385. This compound is even administrated by oral gavage, suggesting highly favorable pharmacological properties. Therefore, it is not clear to me why they used EZN-2208 and not PT2385 for PDX experiments.

The authors should determine how the combination of ATRA+PT2385 (or shRNA +HIF2a) affects cell cycle progression as the cells should stop proliferating upon induction of differentiation. In addition, this combination should be tested in vivo, ideally on PDX models but at least on mice grafted with AML cell lines

Minor:

Fig3d: For the PDX AML2, the difference in the % of cells transduced seems not significant. However, the authors still conclude there is a selective disadvantage for HIF2a KD cells

Method: concentration of ATRA is indicated at 1µM/ml. Does this mean 1µM ?

Referee #3 (Remarks for Author):

EMBO Molecular Medicine

Manuscript Number: EMM-2023-17810

" The transcription factor HIF2 partakes in the differentiation block of acute myeloid leukemia" by Magliulo et al.

In this paper, authors have shown that HIF2 promotes the expression of pro-leukemogenic factors knowingly implicated in suppression of myeloid differentiation via mechanisms of transcription repression. In vivo, they reported that HIF2 plays a prominent role in AML progression, and its pharmacological inhibition (with PT2385 inhibitor) exerts a pro-differentiation and anti-leukemic function. Finally, authors demonstrated that HIF2 inhibition potentiates ATRA-induced differentiation, whilst reducing self-renewal.

Overall, the experiments were designed in rational and data presented support major conclusions.
I recommend to provide genetic invalidation approach (CRISPR-Cas9) in order to support all the data provided with pharmacological inhibition before to before consideration for publication in EMBO Molecular Medicine.

Manuscript EMM-2023-17810

Response to Reviewers

We kindly thank the reviewers for finding our work of interest and for offering very constructive criticisms. We agree with the points that were raised and have addressed them with additional experiments that were especially focused on providing more convincing evidence of the pro-differentiation and anti-leukemic potential of HIF2α targeting in relevant preclinical models. By performing additional *in vivo* experiments with both HIF2α silencing and specific pharmacological inhibition, we believe that we now provide convincing validation of the anti-leukemic potential of HIF2α targeting as well as its function in differentiation.
All new data is provided in the revised version of our manuscript or in this rebuttal letter and is explained in detail in the following pages (in black the reviewers' comments, in blue our responses).

Reviewer 1
In this manuscript, Magliulo et al studied the role of the HIF2a transcription factor in AML differentiation. In particular, they provide evidence that decreased expression of HIF2A induces the differentiation and death of various leukemic cell lines. This correlates with the activation of genes linked to differentitation. They then use inhibitors of HIF2. One of them (EZN2208) is a topoisomerase inhibitor, which induces a decrease in HIF proteins expression. It has antileukemic action and increases differentiation in PDX models. The other, PT2385 is more specific and induces differentation in vitro. In addition, it favors ATRA-induced differentiation of non-APL AML cell lines.
Restoration of leukemic cells differentiation is a sound approach and many AML therapies induce their differentiation. This is of course the case of ATRA but also of Azacitidine, FLT3 or IDH inhibitors. The link between HIF2 and AML differentiation is therefore interesting. However, the conclusions are not well supported by the data and the manuscript lacks convincing evidence of the pro-differentiation/anti-leukemic potential of HIF2 targeting in relevant preclinical models.

We thank this reviewer for finding our work of interest. However, she/he pointed out that our manuscript lacked convincing evidence of the pro-differentiation and anti-leukemic potential of HIF2α targeting in relevant preclinical models and identified 6 major points to be addressed.

Major points:
1. Throughout the manuscript, the effect of HIF2a targeting (shRNA or inhibitors) on differentiation is monitored by the % of CD11b expressing cells. The cell lines used are supposed to be monoclonal and expected to behave homogeneously. It is thus expected that all cells acquire CD11b expression. The authors should also provide representative cytometry profiles as well as histograms representing the average results of Median Fluorescence Intensity for CD11b. This is all the more important that the % of differentiated cells upon HIF2 KD is modest in the non-APL cell lines.
We thank the reviewer for her/his comment. As requested, we now provide representative CD11b cytometry profiles (Appendix Fig S1), show histograms representing the average results of MFI for CD11b (Fig 1C, lower panel), and state at pages 6-7 of the revised manuscript that HIF2α targeting induces a more modest increase in the % of CD11b$^+$ cells in non-APL cell lines than in NB4 cells.
With respect to the reviewer's comment on clonality and homogeneous acquisition of CD11b expression, as a matter of fact we do not expect that HIF2α inhibition will lead to homogeneous induction of CD11b expression and differentiation even in AML cell lines. This is because of the following considerations: i) response of individual cells to signals/stressors is generally not homogeneous even in genetically identical cells, owing to non-genetic transcriptional differences

that are being unveiled by single cell studies. As a recent example, please see Goyal et al., Nature, 2023; PMID: 37468627, where it is shown that intrinsic transcriptional diversity within clonal cancer cells determines differential responses to targeted therapy; ii) non-homogeneous CD11b expression of monoclonal AML cell lines has been similarly reported upon exposure to various pro-differentiative factors or genetic inactivation of specific genes (as examples, please see Sanchez et al., Leukemia, 2014; PMID: 23823656; Schenk et al., Nat Med, 2012; PMID: 22406747; Zhou et al., Haematologica, 2020; PMID: 33054053); iii) modest induction of differentiation in our experimental conditions may also depend on incomplete HIF2α blockade, both via silencing strategies or HIF2α inhibitors.
However, as a more general comment, we wish to clarify to the reviewer that we do not believe that inhibiting HIF2α is sufficient to unleash a complete differentiation program, as we now state at page 18 of the revised manuscript. Our work adds HIF2α to the manifold mechanisms that contribute to the AML differentiation block in the same cells, such that the contribution of HIF2α to AML cell behaviors may be intertwined with intrinsic epigenetic and transcriptional diversities.

2. In addition, the effect on differentiation was measured after 48hrs, which is very early as the differentiation process generally takes up to a week in these cell lines. Kinetics experiments should be performed. The authors should check other markers such as CD14 and CD15, at least on the cell lines.
We thank the reviewer for suggesting to test the kinetics of HIF2α inhibition, which we have now done by treating AML cell lines with the HIF2α inhibitor PT2385 (because HIF2α silencing is constitutive). We performed the requested experiments with a low concentration of PT2385 to avoid cell toxicity, which was observed after 6 days of treatment with higher drug concentrations (Fig EV3C). As anticipated by the reviewer, longer treatment with PT2385 induced increased $CD11b^+$ cells in all cell lines (now shown in Fig 4D, Fig EV3E).
In addition, we have measured CD14 and CD15 expression but have obtained perplexing results in cell lines, as shown in Figures 1 and 2 of this rebuttal letter and explained in detail. The percentage of $CD15^+$ cells is highly variable at baseline in the AML cell lines that we used (Figure 1A), with maximal expression in Kasumi1 and HL60 cells (consistent with expression profiles shown on the German Collection of Microorganisms and Cell Cultures website). Puzzlingly, CD15 expression declined upon *in vitro* culture in all cell lines (Figure 1A). Please, note that at our experimental conditions cells do not become overly crowded at the end of the experiment, fresh medium is added at 4 days to avoid overconsumption, and the decrease of CD15 expression was observed both in mock-treated as well as untreated cells. Thus, we hypothesize that these variations may be induced by cell contacts or metabolites/signaling molecules that accumulate in the culture medium over time.

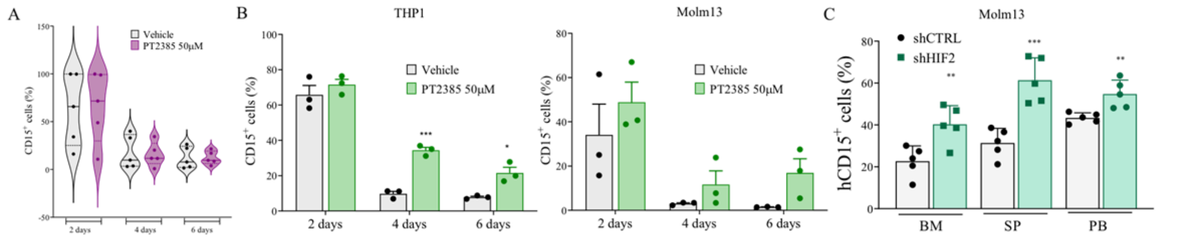

Figure 1. CD15 surface expression in AML cell lines. A. CD15 surface expression in a time course of mock/PT2385 treatment. Each dot represents a cell line. B. Representative experiments in THP1 and Molm13 cells. C. % of human $CD45^+CD15^+$ cells from bone marrow (BM), spleen (SP) and peripheral blood (PB) of Molm13-inoculated mice.

In THP1 and Molm13 cells, but not the other cell lines, we did observe higher CD15 levels upon PT2385 treatment at day 4 and 6 when compared to mock-treated cells (Figure 1B). Also, CD15

expression increased *in vivo* in an additional experiment that was suggested by this reviewer and is further discussed at point 5 (Molm13 cells knocked down for HIF2α; Figure 1C). However, because of baseline variability and *in vitro* modulation of CD15 surface expression in mock-treated cells, for which we lack an explanation, we prefer not to show these data unless required by the reviewer.

Regarding CD14, we also observed culture-induced variations in surface expression in untreated or mock-treated AML cell lines, yet of the opposite direction with respect to CD15 (Figure 2). This was more evident in HL60 and Kasumi1 cells, where $CD14^+$ cells reached 25% and 20% respectively at day 6 (Figure 2). Upon PT2385 treatment, CD14 surface expression increased overall in all cell lines, but with variable behavior. In consideration of this variability and of increased CD14 expression in control HL60 and Kasumi1 cells in culture, a phenomenon for which we do not have an explanation, we prefer not to show these data in the manuscript unless specifically required by the reviewer.

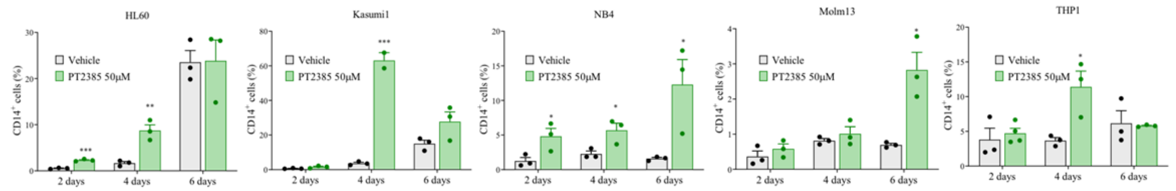

Figure 2. CD14 surface expression in AML cell lines. % of $CD14^+$ cells in a time course of mock/PT2385 treatment.

Nevertheless, we have also measured CD14 and CD15 in another experiment suggested by this reviewer. As discussed in more detail at Point 5, we have used PT2385 for *in vivo* treatment of an AML PDX model and found that increased CD11b expression is accompanied by CD14 and CD15, which we now show in Fig 4H-L. We believe that this will provide more convincing evidence of myeloid differentiation upon HIF2α inhibition, also in consideration of the more physiological *in vivo* context, and hope that the reviewer will agree with us.

3. ATAC-seq and H3K27me3 Chip seq were performed upon HIF2a KD. However, the results of these experiments are not well analyzed. The authors only present the analysis of the changes in chromatin accessibility and H3K27me3 at 3 loci: ITGB2, CD53 and IFI16. This is odd as they even didn't check if HIF2 is actually binding to these genes. What happens on the rest of the genome and at genes up- or down regulated by HIF2? The authors should have performed CHIP-Seq for HIF2 to identify genes, which are directly regulated by this transcription factor and analyze chromatin accessibility at these loci.

We thank this reviewer for her/his request and we now show whole genome analysis of H3K27me3 ChIP-seq and ATAC-seq in Figure EV2 and Appendix Figure S3.

By doing this analysis we found that HIF2α silencing causes a global decrease of H3K27me3 on chromatin (in term of peaks number, coverage and intensity), and decreased protein levels of H3K27me3 (as also observed by Li et al., Cell Reports, 2021; PMID: 34433035), while H3K9me3 expression remains unchanged (Appendix Figure S3A-D). As requested, genes with changes in H3K27me3 were overlapped with HIF2α-regulated genes (Appendix Figure S3E and F). Gene ontology analysis confirmed that genes up- or downregulated upon HIF2α silencing with coherent changes in H3K27me3 were enriched in myeloid differentiation or transcriptional/chromatin regulation respectively (Fig EV2D and E). In sum, these data reveal a general regulation of facultative heterochromatin by HIF2α, which corresponds at least in part to transcriptional regulation. Currently, we do not have mechanistic insights into the role of HIF2α in modulating H3K27me3 levels, because genes belonging to the polycomb repressive complex 2 or histone demethylases are not within the HIF2α-regulated transcriptome. In addition, because a recent

publication reported that HIF2α may play a direct function in H3K27me3 deposition by forming a complex with EZH2 and promoting its chromatin association in mouse macrophages (Li et al., Cell Reports, 2021; PMID: 34433035), we tested the hypothesis that a similar mechanism may be active also in AML cells. However, we could not detect HIF2α/EZH2 interaction via co-immunoprecipitation or proximity ligation assays. Thus, although we were able to confirm an important role of HIF2α in the regulation of H3K27me3, our data suggest that the role of HIF2α in promoting H3K27me3 modifications in AML may be indirect.

In light of this new analysis, we removed H3K27me3 profiles at *ITGB2*, *CD53* and *IFI16* promoters (previous Supplementary Figure 4A, now Fig EV2F), as they are not anymore representative of whole classes of myeloid differentiation genes. However, we underline to the reviewer that our new analysis confirmed lack of H3K27me3 peaks in the promoter/enhancer regions of *ITGB2* and *IFI16*, while a more distal enhancer region with a HIF2α-regulated H3K27me3 peak was linked to the *CD53* gene.

As requested by this reviewer, in the revised manuscript we also show the overlap between whole genome accessibility analysis (ATACseq) and HIF2α-regulated genes. This analysis confirmed that genes positively regulated by HIF2α with coherent changes in chromatin accessibility are enriched in families linked to transcriptional regulation (Appendix Fig S3H) and contain validated genes like *BCL11A*. Interestingly, myeloid differentiation genes negatively regulated by HIF2α did not show changes in chromatin accessibility (Appendix Fig S3G). Thus, by performing a complete analysis of chromatin accessibility, we confirmed what we had shown in previous Supplementary Figure 4 (now Fig EV2F), namely that at large genes linked to myeloid differentiation do not show changes in ATAC profiles upon HIF2α silencing because they are not in a close chromatin state in control cells.

Finally, this reviewer had also suggested to map direct HIF2α target genes via HIF2α ChIP-seq. Unfortunately, although we have attempted to do this experiment several times over the past few years, we have always been unsuccessful. First, we have been unable to isolate sizable amounts of DNA upon immunoprecipitation of endogenous HIF2α in AML cells because of low HIF2α expression or antibodies inefficacy. To stabilize HIF2α, we exposed AML cell lines to hypoxic conditions but observed profound phenotypic changes, such as proliferation arrest and cell death. While this is coherent with cell lines being adapted to 20% oxygen, it prevented us from using this condition to validate a phenotype that is linked to a proliferation arrest. Thus more recently, to answer to the request of this reviewer, we have transduced AML cells with an inducible construct expressing a stable form of HIF2α. However, even upon HIF2α induction, which correlated with increased association with its obliged transcriptional partner HIF1β and upregulation of HIF2α-regulated genes (now in Fig 2I-N), we were still unable to immunoprecipitate sizable amounts of DNA for sequencing, which we attribute to low immunoprecipitation efficiency by HIF2α antibodies. CUT&Tag experiments upon HIF2α induction were also unsuccessful because of low specificity of HIF2α antibodies in immunofluorescence.

We are really sorry that we have not been able to map HIF2α DNA binding. However, induction of exogenous HIF2α was sufficient to confirm HIF2α association to the regulatory regions of two important genes involved in suppressing AML differentiation (*BCL11A* and *RUNX2*, Fig 2N), thus confirming their direct regulation by HIF2α.

4. As the authors didn't see an effect of HIF KD on differentiation in PDX models, they resorted to EZN-2208. They write that it « acts as a potent HIFa inhibitor when used at non-cytotoxic concentration ». Then they write «this suggests that acute pharmacological inhibition of HIF2a...» This is clearly misleading as it suggests that this drug directly targets HIF protein. This is not the case. EZN-2208 is a toposisomerase inhibitor. Although it clearly decreases HIF expression, it has many other effects and the authors can clearly not conclude that inhibition of HIF2a exerts a pro-differentiation role.

We agree that EZN2208 is not a specific HIF inhibitor. It is a derivative of campothecin, which as the reviewer rightly points out is a topoisomerase inhibitor, as we now state at page 14. Although the literature that we quote and our previous work demonstrates that this compound also inhibits HIF factors at non-cytotoxic concentrations, we think that the reviewer's point is well taken and even if we observed HIF2α inhibition in the absence of cell death we cannot conclude that the anti-leukemic and differentiation effects of EZN2208 are solely due to HIF2α inhibition. For this reason, we have moved these experiments to Expanded View information as a confirmatory experiment. Also, in describing the data, we have toned down our conclusions and have clearly stated that EZN2208 may also exert additional effects on other molecular targets (page 14). We chose not to remove these experiments altogether because they provide useful information on EZN2208 anti-leukemic properties and identify an additional compound that triggers AML differentiation.

5. Instead, the authors should perform in vivo experiments using AML cell lines knocked-down or knocked-out by CRIPSR for HIF2a and monitor tumor progression and differentiation. Similar to in vitro experiments, more differentiation markers should be used (at least CD14 and CD15). At least, they should use the HIF specific inhibitor PT2385 in the PDX model in vivo and not only ex vivo. The authors state that they only performed in vitro experiments with PT2385 because the molecule for in vivo studies was not available. However, in the paper they cite (Wallace et al, 2016), in vivo experiments were performed using PT2385. This compound is even administrated by oral gavage, suggesting highly favorable pharmacological properties. Therefore, it is not clear to me why they used EZN-2208 and not PT2385 for PDX experiments.

We thank this reviewer for her/his suggestions to perform additional *in vivo* experiments with HIF2α knockdown in cell lines and PT2385 in a PDX model. We have performed these experiments and added substantial new data to our manuscript, which we hope the reviewer will find convincing.

For *in vivo* experiments with cell lines, we have used Kasumi1 and Molm13 cells because based on published literature and our own experience they engraft efficiently *in vivo*, with Kasumi1 being injected subcutaneously (see for example Li et al., EMBO Mol Med, 2017; PMID: 28539478; Neldeborg et al., Leukemia, 2023; PMID: 37464068), and Molm13 intravenously (Migliavacca et al., Oncotarget, 2016; PMID: 27447550). With both cell lines we found that HIF2α knockdown caused a significant reduction in leukemia progression (Fig 3D-M). Regarding induction of differentiation, in Kasumi1 cells we observed transcriptional upregulation of representative myeloid differentiation genes (Fig 3F) and a non-significant increase in $CD11b^+$ cells (Appendix Fig S4D), while in Molm13 cells transcriptional upregulation of myeloid differentiation genes (Fig 3L) was accompanied by increased % of CD11b expression in more physiologically relevant leukemia environments (bone marrow, spleen and peripheral blood, Fig 3I).

In addition, we have used the specific HIF2α inhibitor PT2385 in an *in vivo* PDX model. This experiment had not been performed earlier because PT2385 was either not available on the market (the paper by Wallace et al., 2016 was published by Peloton Therapeutics, the company that had developed PT2385) or had been extremely costly (when it became available). However, because the drug recently became more reasonably obtainable, we have performed the experiment as described by Wallace et al. and now provide further validation of the function of HIF2α in AML and the potential of its targetability. As we now show in Fig 4, treatment with PT2385 reduced AML burden in bone marrow, spleen and peripheral blood and caused increased expression of CD11b, CD15 and CD14 in all compartments (Fig 4E-L).

6. The authors should determine how the combination of ATRA+PT2385 (or shRNA +HIF2a) affects cell cycle progression as the cells should stop proliferating upon induction of differentiation. In addition, this combination should be tested in vivo, ideally on PDX models but at least on mice grafted with AML cell lines

We thank this reviewer for her/his suggestion to measure cell cycle distribution upon ATRA+PT2385. We have done this experiment in all AML cell lines and now show that combined treatment causes a significant accumulation in G0/G1 accompanied by decreased S phase (Fig 5C, Fig EV5C).
With respect to the *in vivo* experiment with ATRA+PT2385, we agree that this is a very important experiment. Unfortunately, this is the only experiment that we were unable to perform at present time because ATRA pellets are provided by a USA-based company that takes 3/4 months to deliver the product to our research center. Also, we believe that the combination of ATRA+PT2385 should be tested in multiple AML models and with various treatment schedules to provide strong preclinical data for a possible clinical translatability. Thus, we feel that these experiments should be part of a thorough follow-up work. We hope that our current manuscript will provide proof-of-concept evidence that will allow us to obtain further funding for these important experiments.

Minor points:
1. Fig3d: For the PDX AML2, the difference in the % of cells transduced seems not significant. However, the authors still conclude there is a selective disadvantage for HIF2a KD cells
The reviewer rightly points out that for PDX AML-02 the difference in the % of OFP+ cells is not significant (now Fig 3B). We have so stated at page 12 of the revised manuscript.

2. Method: concentration of ATRA is indicated at 1µM/ml. Does this mean 1µM?
We are sorry for this mistake and have corrected it to 1 µM.

Reviewer 2
In this paper, authors have shown that HIF2a promotes the expression of pro-leukemogenic factors knowingly implicated in suppression of myeloid differentiation via mechanisms of transcription repression. In vivo, they reported that HIF2a plays a prominent role in AML progression, and its pharmacological inhibition (with PT2385 inhibitor) exerts a pro-differentiation and anti-leukemic function. Finally, authors demonstrated that HIF2a inhibition potentiates ATRA-induced differentiation, whilst reducing self-renewal.

Overall, the experiments were designed in rational and data presented support major conclusions.
I recommend to provide genetic invalidation approach (CRISPR-Cas9) in order to support all the data provided with pharmacological inhibition before to before consideration for publication in EMBO Molecular Medicine.

We thank this reviewer for her/his appreciation of our work. The reviewer recommended that we supported the data provided with pharmacological inhibition by performing genetic invalidation of HIF2α via CRISPR-Cas9 targeting.
As requested by the reviewer, we have performed this experiment in the two cell lines that showed higher recovery rates upon electroporation, Kasumi1 and Molm13 cells. We have used 3 guide RNAs (gRNA) against *EPAS1* (HIF2α gene) that were selected from predesigned Alt-R CRISPR-Cas9 guides (IDT, Integrated DNA Technologies). Two of these guides overlapped with gRNAs previously utilized to knock-out *EPAS1* in other cell types (Wierenga et al., Cancer Metab, 2019; PMID: 31890203; Stransky et al., PNAS, 2022; PMID: 35357972). Upon electroporation of preformed Cas9/gRNA complexes, we recovered 60 single cell-derived clones of Kasumi1 cells and 30 clones of Molm13 cells. All recovered clones were screened by PCR analysis with primers

surrounding the region of Cas9-induced DNA cutting. This screening identified only heterozygous *EPAS1* targeting in Kasumi1 cells (a total of 14 heterozygous clones, representative examples in Figure 3), suggesting that complete *EPAS1* knock-out is not tolerated in these cell lines. This hypothesis is consistent with previous literature: *EPAS1* knock-out has been described in a model of chronic myelogenous leukemia (K562 cells, Wierenga et al., Cancer Metab, 2019; PMID: 31890203), but not in AML cells, where HIF2α expression was downregulated via silencing strategies (Roualt-Pierre et al., Cell Stem Cell, 2013; PMID: 24095676).

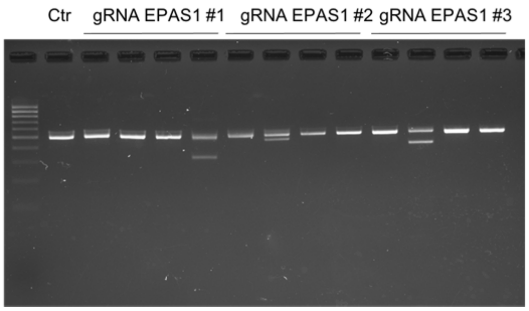

Figure 3. PCR analysis of the EPAS1 gene. Shown is a representative example of Kasumi1 clones screening. Ctr indicates Kasumi1 cells transduced with a control gRNA that does not target any human sequence. A band of 500 bps corresponds to wt *EPAS1*. Lower bands of different sizes represent *EPAS1* targeting in heterozygosity.

This new experiment also adds clearer context to previous observations that we have obtained upon HIF2α knock-down. We have observed that HIF2α silencing is counter selected *in vitro* in AML cell lines, as we now show and discuss in the revised manuscript (Fig EV1C; page 6). Specifically, while HIF2α silencing efficacy is high at early passages upon shRNA lentiviral transduction, *in vitro* passaging caused diminished HIF2α silencing, which was consistently observed at every new round of lentiviral infection and coincided with a gradual loss of phenotype starting approximately at passage 10. Because HIF2α silencing impairs proliferation (Fig 1B, Fig EV1B), we believe that cells with higher shRNA expression are being gradually counter selected and lost in the cell population. These *in vitro* observations are also supported by *in vivo* data, where we confirmed loss of HIF2α silencing in multiple experiments now described in the revised manuscript (Fig 3C, G and M).

In conclusion, we again thank both reviewers for their support and constructive criticisms. In performing the experiments that they have suggested we hope that we are now providing convincing evidence of the role of HIF2α in AML progression and differentiation arrest.

11th Sep 2023

Dear Dr. Bernardi,

Thank you for the submission of your revised manuscript to EMBO Molecular Medicine. We have now received the reports from the 2 referees who re-reviewed your manuscript. As you will see, they are supportive of publication, and we will therefore be able to accept your manuscript once the following editorial points will be addressed:

1/ Main manuscript file:
- Please address the queries from our data editors in the related Data Edited manuscript file. Remove the red text and only keep in track changes mode any new modification.
- Please remove "not shown" (p.7 and p.10). As per our guidelines, on "Unpublished Data" the journal does not permit citation of "Data not shown". All data referred to in the paper should be displayed in the main or Expanded View figures.
- Materials and methods:
o Mice: please indicate the gender of the mice used for the experiments.
o Antibodies: please provide the dilutions/concentrations used for all experiments
- Thank you for providing a Data Availability section. Please note that this section is restricted to new primary data that are part of this study.
- Acknowledgements: All funding sources should be added to the submission system, and match the information provided in the manuscript (currently missing in the submission system: the Giovanni Armenise-Harvard Foundation with a Career Development Award, Donazione Fronzaroli (postdoctoral fellowship), MUR (Ministry of University and Research) and Vita-Salute San Raffaele University (PhD fellowship)).
- Please rename the "conflict of interest" section to "Disclosure statement and competing interests". We updated our journal's competing interests policy in January 2022 and request authors to consider both actual and perceived competing interests. Please review the policy https://www.embopress.org/competing-interests and update your competing interests if necessary.

2/ Data citation:
Please check the comment from our data editor in the Data edited manuscript file and adjust the formatting accordingly.

3/ Figures and Appendix:
- Please provide exact p values, not a range, in the figures or in their legends (including for EV figures).
- Please upload EV figures as Figure files (instead of Expanded View)

4/ At EMBO Press we ask authors to provide source data for the main figures. Our source data coordinator has contacted you and listed which figure panels we would need source data for and provided indications on how to upload and organize the files. Please find the checklist attached to this email again for your convenience.

5/ Synopsis: Thank you for providing a nice synopsis text and image. Please upload the synopsis image individually in jpeg, TIFF or png format and sized 550 pixels wide x 200-400 pixels high.

6/ As part of the EMBO Publications transparent editorial process initiative (see our Editorial at http://embomolmed.embopress.org/content/2/9/329), EMBO Molecular Medicine will publish online a Review Process File (RPF) to accompany accepted manuscripts.
This file will be published in conjunction with your paper and will include the anonymous referee reports, your point-by-point response and all pertinent correspondence relating to the manuscript. Let us know whether you agree with the publication of the RPF and as here, if you want to remove or not any figures from it prior to publication.
Please note that the Authors checklist will be published at the end of the RPF.

I look forward to receiving your revised manuscript.

Yours sincerely,

Lise Roth

Lise Roth, PhD
Senior Editor
EMBO Molecular Medicine

***** Reviewer's comments *****

Referee #1 (Remarks for Author):

In this revised manuscript, Magliulo et al have addressed all my concerns. In particular, they added new in vivo data, using both cell lines (MOLM13 and Kasumi) with KD for HIF2a and PDX with the PT2385 inhibitor. These results, as well as the other addition experiments performed strongly reinforce the conclusions of this manuscript.

Referee #3 (Remarks for Author):

The authors have sufficiently addressed my comments. The manuscript is significantly improved compared to the original submission.

The authors addressed the remaining editorial issues.

21st Sep 2023

Dear Dr. Bernardi,

Thank you for submitting your revised files. I am pleased to inform you that your manuscript is accepted for publication and is now being sent to our publisher to be included in the next available issue of EMBO Molecular Medicine!

We would like to remind you that as part of the EMBO Publications transparent editorial process initiative, EMBO Molecular Medicine will publish a Review Process File online to accompany accepted manuscripts. If you do NOT want the file to be published or would like to exclude figures, please immediately inform the editorial office via e-mail.

Please read below for additional IMPORTANT information regarding your article, its publication and the production process.

Congratulations on your interesting work,

With kind regards,

Lise Roth

Lise Roth, Ph.D
Senior Editor
EMBO Molecular Medicine

Follow us on Twitter @EmboMolMed
Sign up for eTOCs at embopress.org/alertsfeeds

*** *** *** IMPORTANT INFORMATION *** *** ***

SPEED OF PUBLICATION
The journal aims for rapid publication of papers, using using the advance online publication "Early View" to expedite the process: A properly copy-edited and formatted version will be published as "Early View" after the proofs have been corrected. Please help the Editors and publisher avoid delays by providing e-mail address(es), telephone and fax numbers at which author(s) can be contacted.

Should you be planning a Press Release on your article, please get in contact with embomolmed@wiley.com as early as possible, in order to coordinate publication and release dates.

LICENSE AND PAYMENT:

All articles published in EMBO Molecular Medicine are fully open access: immediately and freely available to read, download and share.

EMBO Molecular Medicine charges an article processing charge (APC) to cover the publication costs. You, as the corresponding author for this manuscript, should have already received a quote with the article processing fee separately. Please let us know in case this quote has not been received.

Once your article is at Wiley for editorial production you will receive an email from Wiley's Author Services system, which will ask you to log in and will present you with the publication license form for completion. Within the same system the publication fee can be paid by credit card, an invoice, pro forma invoice or purchase order can be requested.

Payment of the publication charge and the signed Open Access Agreement form must be received before the article can be published online.

PROOFS

You will receive the proofs by e-mail approximately 2 weeks after all relevant files have been sent o our Production Office.

Please return them within 48 hours and if there should be any problems, please contact the production office at embopressproduction@wiley.com.

Please inform us if there is likely to be any difficulty in reaching you at the above address at that time. Failure to meet our deadlines may result in a delay of publication.

All further communications concerning your paper proofs should quote reference number EMM-2023-17810-V3 and be directed to the production office at embopressproduction@wiley.com.

EMBO Press Author Checklist

Corresponding Author Name: Bernardi Rosa	
Journal Submitted to: EMBO Molecular Medicine	
Manuscript Number: EMM-2023-17810	

USEFUL LINKS FOR COMPLETING THIS FORM
The EMBO Journal - Author Guidelines
EMBO Reports - Author Guidelines
Molecular Systems Biology - Author Guidelines
EMBO Molecular Medicine - Author Guidelines

Reporting Checklist for Life Science Articles (updated January
This checklist is adapted from Materials Design Analysis Reporting (MDAR) Checklist for Authors. MDAR establishes a minimum set of requirements in transparent reporting in the life sciences (see Statement of Task: 10.31222/osf.io/9sm4x). Please follow the journal's guidelines in preparing your
Please note that a copy of this checklist will be published alongside your article.

Abridged guidelines for figures
1. Data
The data shown in figures should satisfy the following conditions:

- the data were obtained and processed according to the field's best practice and are presented to reflect the results of the experiments in an accurate and unbiased manner.
- ideally, figure panels should include only measurements that are directly comparable to each other and obtained with the same assay.
- plots include clearly labeled error bars for independent experiments and sample sizes. Unless justified, error bars should not be shown for technical
- if n<5, the individual data points from each experiment should be plotted. Any statistical test employed should be justified.
- Source Data should be included to report the data underlying figures according to the guidelines set out in the authorship guidelines on Data

2. Captions
Each figure caption should contain the following information, for each panel where they are relevant:

- a specification of the experimental system investigated (eg cell line, species name).
- the assay(s) and method(s) used to carry out the reported observations and measurements.
- an explicit mention of the biological and chemical entity(ies) that are being measured.
- an explicit mention of the biological and chemical entity(ies) that are altered/varied/perturbed in a controlled manner.
- the exact sample size (n) for each experimental group/condition, given as a number, not a range;
- a description of the sample collection allowing the reader to understand whether the samples represent technical or biological replicates (including how many animals, litters, cultures, etc.).
- a statement of how many times the experiment shown was independently replicated in the laboratory.
- definitions of statistical methods and measures:
  - common tests, such as t-test (please specify whether paired vs. unpaired), simple χ2 tests, Wilcoxon and Mann-Whitney tests, can be unambiguously identified by name only, but more complex techniques should be described in the methods section;
  - are tests one-sided or two-sided?
  - are there adjustments for multiple comparisons?
  - exact statistical test results, e.g., P values = x but not P values < x;
  - definition of 'center values' as median or average;
  - definition of error bars as s.d. or s.e.m.

Please complete ALL of the questions below.
Select "Not Applicable" only when the requested information is not relevant for your study.

Materials

Newly Created Materials	Information included in the manuscript?	In which section is the information available? (Reagents and Tools Table, Materials and Methods, Figures, Data Availability Section)
New materials and reagents need to be available; do any restrictions apply?	Yes	Materials and Methods

Antibodies	Information included in the manuscript?	In which section is the information available? (Reagents and Tools Table, Materials and Methods, Figures, Data Availability Section)
For **antibodies** provide the following information:<br>- Commercial antibodies: RRID (if possible) or supplier name, catalogue number and or/clone number<br>- Non-commercial: RRID or citation	Yes	Materials and Methods

DNA and RNA sequences	Information included in the manuscript?	In which section is the information available? (Reagents and Tools Table, Materials and Methods, Figures, Data Availability Section)
**Short novel DNA or RNA including primers, probes**: provide the sequences.	Yes	Supplementary Tables

Cell materials	Information included in the manuscript?	In which section is the information available? (Reagents and Tools Table, Materials and Methods, Figures, Data Availability Section)
**Cell lines:** Provide species information, strain. Provide accession number in repository **OR** supplier name, catalog number, clone number, and/**OR** RRID.	Yes	Materials and Methods
**Primary cultures:** Provide species, strain, sex of origin, genetic modification status.	Yes	Materials and Methods
Report if the cell lines were recently **authenticated** (e.g., by STR profiling) and tested for mycoplasma contamination.	Yes	Materials and Methods

Experimental animals	Information included in the manuscript?	In which section is the information available? (Reagents and Tools Table, Materials and Methods, Figures, Data Availability Section)
**Laboratory animals or Model organisms:** Provide species, strain, sex, age, genetic modification status. Provide accession number in repository **OR** supplier name, catalog number, clone number, **OR** RRID.	Yes	Materials and Methods
**Animal observed in or captured from the field:** Provide species, sex, and age where possible**.**	Not Applicable	
Please detail **housing and husbandry conditions**.	Yes	Materials and Methods

Plants and microbes	Information included in the manuscript?	In which section is the information available? (Reagents and Tools Table, Materials and Methods, Figures, Data Availability Section)
**Plants:** provide species and strain, ecotype and cultivar where relevant, unique accession number if available, and source (including location for collected wild specimens).	Not Applicable	
**Microbes:** provide species and strain, unique accession number if available, and source.	Not Applicable	

Human research participants	Information included in the manuscript?	In which section is the information available? (Reagents and Tools Table, Materials and Methods, Figures, Data Availability Section)
If collected and within the bounds of privacy constraints report on age, sex and gender or ethnicity for all study participants.	Not Applicable	

Core facilities	Information included in the manuscript?	In which section is the information available? (Reagents and Tools Table, Materials and Methods, Figures, Data Availability Section)
If your work benefited from core facilities, was their service mentioned in the acknowledgments section?	Yes	Acknowledgments section

Design

Study protocol	Information included in the manuscript?	In which section is the information available? (Reagents and Tools Table, Materials and Methods, Figures, Data Availability Section)
If study protocol has been **pre-registered, provide DOI in the manuscript**. For clinical trials, provide the trial registration number **OR** cite DOI.	Not Applicable	
Report the **clinical trial registration number** (at ClinicalTrials.gov or equivalent), where applicable.	Not Applicable	

Laboratory protocol	Information included in the manuscript?	In which section is the information available? (Reagents and Tools Table, Materials and Methods, Figures, Data Availability Section)
Provide DOI OR other citation details if **external detailed step-by-step protocols** are available.	Yes	Materials and Methods

Experimental study design and statistics	Information included in the manuscript?	In which section is the information available? (Reagents and Tools Table, Materials and Methods, Figures, Data Availability Section)
Include a statement about **sample size** estimate even if no statistical methods were used.	Yes	Materials and Methods
Were any steps taken to minimize the effects of subjective bias when allocating animals/samples to treatment (e.g. **randomization procedure**)? If yes, have they been described?	Yes	Materials and Methods
Include a statement about **blinding** even if no blinding was done.	Yes	Materials and Methods
Describe **inclusion/exclusion criteria** if samples or animals were excluded from the analysis. Were the criteria pre-established?<br><br>If sample or data points were omitted from analysis, report if this was due to attrition or intentional exclusion and provide justification.	Not Applicable	
For every figure, are **statistical tests** justified as appropriate? Do the data meet the assumptions of the tests (e.g., normal distribution)? Describe any methods used to assess it. Is there an estimate of variation within each group of data? Is the variance similar between the groups that are being statistically compared?	Yes	Materials and Methods

Sample definition and in-laboratory replication	Information included in the manuscript?	In which section is the information available? (Reagents and Tools Table, Materials and Methods, Figures, Data Availability Section)
In the figure legends: state number of times the experiment was **replicated** in laboratory.	Yes	Figure Legends
In the figure legends: define whether data describe **technical or biological replicates**.	Yes	Figure Legends

Ethics

Ethics	Information included in the manuscript?	In which section is the information available? (Reagents and Tools Table, Materials and Methods, Figures, Data Availability Section)
Studies involving **human participants**: State details of **authority granting ethics approval** (IRB or equivalent committee(s), provide reference number for approval.	Not Applicable	
Studies involving **human participants**: Include a statement confirming that **informed consent** was obtained from all subjects and that the experiments conformed to the principles set out in the WMA Declaration of Helsinki and the Department of Health and Human Services Belmont Report.	Not Applicable	
Studies involving **human participants: For publication of patient photos**, include a statement confirming that consent to publish was obtained.	Not Applicable	
Studies involving experimental **animals**: State details of **authority granting ethics approval** (IRB or equivalent committee(s), provide reference number for approval. Include a statement of compliance with ethical regulations.	Yes	Materials and Methods
Studies involving **specimen and field samples:** State if relevant **permits** obtained, provide details of authority approving study; if none were required, explain why.	Not Applicable	

Dual Use Research of Concern (DURC)	Information included in the manuscript?	In which section is the information available? (Reagents and Tools Table, Materials and Methods, Figures, Data Availability Section)
Could your study fall under dual use research restrictions? Please check biosecurity documents and list of **select agents and toxins** (CDC): https://www.selectagents.gov/sat/list.htm	Not Applicable	
If you used a select agent, is the security level of the lab appropriate and reported in the manuscript?	Not Applicable	
If a study is subject to dual use research of concern regulations, is the name of the **authority granting approval and reference number** for the regulatory approval provided in the manuscript?	Not Applicable	

Reporting

The MDAR framework recommends adoption of discipline-specific guidelines, established and endorsed through community initiatives. Journals have their own policy about requiring specific guidelines and recommendations to complement MDAR.

Adherence to community standards	Information included in the manuscript?	In which section is the information available? (Reagents and Tools Table, Materials and Methods, Figures, Data Availability Section)
State if relevant guidelines or checklists (e.g., **ICMJE, MIBBI, ARRIVE, PRISMA**) have been followed or provided.	Not Applicable	
For **tumor marker prognostic studies,** we recommend that you follow the **REMARK** reporting guidelines (see link list at top right). See author guidelines, under 'Reporting Guidelines'. Please confirm you have followed these guidelines.	Not Applicable	
For **phase II and III randomized controlled trials**, please refer to the **CONSORT** flow diagram (see link list at top right) and submit the CONSORT checklist (see link list at top right) with your submission. See author guidelines, under 'Reporting Guidelines'. Please confirm you have submitted this list.	Not Applicable	

Data Availability

Data availability	Information included in the manuscript?	In which section is the information available? (Reagents and Tools Table, Materials and Methods, Figures, Data Availability Section)
Have **primary datasets** been deposited according to the journal's guidelines (see 'Data Deposition' section) and the respective accession numbers provided in the Data Availability Section?	Yes	Data Availability
Were **human clinical and genomic datasets** deposited in a public access-controlled repository in accordance to ethical obligations to the patients and to the applicable consent agreement?	Not Applicable	
Are **computational models** that are central and integral to a study available without restrictions in a machine-readable form? Were the relevant accession numbers or links provided?	Not Applicable	
If publicly available data were reused, provide the respective **data citations in the reference list**.	Yes	Reference list
